# Axenic Long-Term Cultivation of *Pneumocystis jirovecii*

**DOI:** 10.3390/jof9090903

**Published:** 2023-09-01

**Authors:** Diana Riebold, Marie Mahnkopf, Kristina Wicht, Cristina Zubiria-Barrera, Jan Heise, Marcus Frank, Daniel Misch, Torsten Bauer, Hartmut Stocker, Hortense Slevogt

**Affiliations:** 1 Research Centre of Medical Technology and Biotechnology (FZMB), 99947 Bad Langensalza, Germany; mmahnkopf@fzmb.de (M.M.); jheise@fzmb.de (J.H.); 2 Separation Science Group, Department of Organic and Macromolecular Chemistry, Ghent University, B-9000 Gent, Belgium; wicht_kristina@yahoo.de; 3 Respiratory Infection Dynamics Group, Helmholtz Centre for Infection Research, 38124 Braunschweig, Germany; cristina.zubiriabarrera@helmholtz-hzi.de (C.Z.-B.); slevogt.hortense@mh-hannover.de (H.S.); 4 Department of Respiratory Medicine and Infectious Diseases, Hannover Medical School, German Center for Lung Research (DZL), BREATH, 30625 Hannover, Germany; 5 Medical Biology and Electron Microscopy Centre (EMZ), University Medicine Rostock, 18057 Rostock, Germany; marcus.frank@med.uni-rostock.de; 6 Lungenklinik Heckeshorn, Helios Klinikum Emil-von-Behring, 14165 Berlin, Germany; daniel.misch@helios-gesundheit.de (D.M.); torsten.bauer@helios-gesundheit.de (T.B.); 7 Clinic for Infectiology, St. Joseph’s Hospital Berlin, 12101 Berlin, Germany; hartmut.stocker@sjk.de

**Keywords:** *Pneumocystis*, *Pneumocystis jirovecii*, culture, axenic, human lung carcinoma cells, A549, DMEM

## Abstract

*Pneumocystis jirovecii*, a fungus causing severe *Pneumocystis* pneumonia (PCP) in humans, has long been described as non-culturable. Only isolated short-term experiments with *P. jirovecii* and a small number of experiments involving animal-derived *Pneumocystis* species have been published to date. However, *P. jirovecii* culture conditions may differ significantly from those of animal-derived *Pneumocystis*, as there are major genotypic and phenotypic differences between them. Establishing a well-performing *P. jirovecii* cultivation is crucial to understanding PCP and its pathophysiological processes. The aim of this study, therefore, was to develop an axenic culture for *Pneumocystis jirovecii*. To identify promising approaches for cultivation, a literature survey encompassing animal-derived *Pneumocystis* cultures was carried out. The variables identified, such as incubation time, pH value, vitamins, amino acids, and other components, were trialed and adjusted to find the optimum conditions for *P. jirovecii* culture. This allowed us to develop a medium that produced a 42.6-fold increase in *P. jirovecii* qPCR copy numbers after a 48-day culture. Growth was confirmed microscopically by the increasing number and size of actively growing *Pneumocystis* clusters in the final medium, DMEM-O3. *P. jirovecii* doubling time was 8.9 days (range 6.9 to 13.6 days). In conclusion, we successfully cultivated *P. jirovecii* under optimized cell-free conditions in a 70-day long-term culture for the first time. However, further optimization of the culture conditions for this slow grower is indispensable.

## 1. Introduction

*Pneumocystis jirovecii*, a human-specific pathogenic fungus, causes life-threateningly severe *Pneumocystis* pneumonia (PCP) in immunocompromised patients, with 400,000 estimated cases per year worldwide [1,2].

*Pneumocystis* species have an unusual life cycle that has not yet been fully elucidated due to the lack of long-term cultures [3]. Obtaining nutrition from living host cells seems to be essential for *Pneumocystis* replication and explains the high tropism to the host lung [4,5]. Molecular studies have shown markedly genotypical variations between the species having reduced genomes, such as absent genes for *α-glucan* and myo-inositol pathways in *P. jirovecii*, *P. murina*, and *P. carinii* [6,7,8,9,10,11]. Rat-derived *P. carinii* has been described as having underrepresented genes for amino acid metabolism pathways [3,5,6,11,12], suggesting that it may be unable to survive independently from its host [5].

As far as is known, *Pneumocystis* spp. replicate in an asexual phase via thin-walled haploid trophic forms, previously known as trophozoites, which are 2 to 4 μm in size. During the sexual phase, trophic forms fuse into a 2 to 8 μm large diploid trophic form, which develops further into an ascus (previously known as a cyst, 10 μm). These ascii contain eight spores (formerly intracystic bodies or sporozoites) when mature. Sporozoites are then released and begin their lives as trophic forms, which often cluster and are the predominant form in the lungs [3,6].

Establishing a stable *P. jirovecii* culture is crucial for the development and standardization of diagnostic methods. It is also necessary for a better understanding of the disease PCP, for a better understanding of the life cycle and metabolism of the pathogen, for a better understanding of in vitro resistance, and for drug testing.

Only four attempts to culture *P. jirovecii* have been published, and all four were insufficiently successful or generated results that could not be reproduced by other research groups [13,14,15,16,17,18]. Cushion et al. set up a single successful *P. jirovecii* culture on A549 cells with a tenfold increase after 14 days but did not mention conditions or procedures [13]. *P. jirovecii* was grown on CuFi-8 airway cells for two to five days [15], but those results were not reproducible by a well-experienced working group either [16,19]. To date, there are no published reports of successful long-term *P. jirovecii* cultures with or without human or animal-derived feeder cells (axenic culture) [3].

Methods successfully used for rat-derived *Pneumocystis* strains have worked poorly for the human pathogenic *P. jirovecii* so far [14,20]. Nevertheless, combined with culture methods for other fungi and bacteria, they might provide a starting point for finding appropriate methods for *P. jirovecii* culture. Some research groups have been able to maintain animal-derived *Pneumocystis* spp. in vitro (mainly *P. carinii* from rats and *P. murina* from mice) using feeder cells such as A549, Vero, or WI-38, but only cultured them for five to 14 days [21]. As the fungus has a long generation time, varying between two days (*P. oryctolagi*) and eleven days (*P. murina*) [22,23], this might not be sufficient to allow it to reproduce.

The first in vitro culture experiments in the late 1950s were based on solid standard media for parasites and fungi such as Sabouraud and NNN agar [24,25], but they were not successful. In vitro culture of animal-derived *Pneumocystis* spp. then focused on cultivation with liquid cell culture media (RPMI-1640, MEM, Eagle’s medium, and F-12) on top of feeder cells such as CEL [18], Vero cells [26], L2 cells [27,28], WI-38 [29], and, predominantly, A549 cells [14,29]. Cultures were maintained in coated and uncoated culture plates [14,15,29,30,31] or transwell plates to facilitate the separation of *Pneumocystis* spp. from feeder cells [15,30,31]. Some authors described positive effects from roller- or spinner flasks or shaking [32,33], but others reported movement having a negative effect [29,34]. Most supplements for animal-derived *Pneumocystis* cultures were used empirically without testing concentration series [29]. In 1990, Cushion et al. described the first axenic culture for rat- and mouse-derived *P. carinii* [35,36,37,38]. In most cultures, the incubation time under optimal conditions of 5% CO_2_ and 35 to 37 °C varied between 2 and 7 days, with some cultures maintained for up to 96 days [30,39,40,41].

In summary, *P. carinii* and *P. murina* have been shown to be cultivable under certain conditions [20,30,37,42,43], but there is only limited evidence that *P. jirovecii* culture might be possible in vitro [13,14,15,17,18].

Our goal was to establish a culture system for the cultivation of *P. jirovecii*. After intensive literature analysis and a long optimization process, we succeeded in culturing *P. jirovecii* using A549 cells as feeder cells. Upon conversion to an axenic culture system, *P. jirovecii* continued to replicate. The minimum 48-day culture duration was sufficient for multiple replications of this slow-growing organism.

## 2. Materials and Methods

### 2.1. Pneumocystis Culture Literature Review

To identify potential media, conditions, and supplements for *P. jirovecii* culture, a literature review of all *Pneumocystis* cultures was carried out. A distinction was made between papers concerned with the culture of human *P. jirovecii* strains and those concerned with animal-derived *Pneumocystis*. Between 2015 and 2022, repeated literature research was undertaken. Search terms were ‘*Pneumocystis*’ combined with ‘culture’, ‘cultivation’, ‘axenic’, and ‘in vitro’. To find out more about the potential effects of individual supplements, we extended our search to other fungi and organisms using the search terms “culture” and “in vitro culture” in combination with “*Candida*”, “*Aspergillus*”, “*Histoplasma*”, “*Coccidioides*”, “*Paracoccidioides*”, and “*Blastomyces*”.

### 2.2. Sample Collection, Transport, and Freezing Conditions

From March 2016 to January 2019, broncho-alveolar fluid (BALF) samples from patients with suspected *Pneumocystis* pneumonia (PCP) were collected in the Center for Infectious Diseases and HIV, Vivantes Auguste-Victoria-Hospital, Berlin, the “Lungenklinik Heckeshorn”, HELIOS Klinikum Emil-von-Behring Hospital, Berlin, the Department for Infectious Diseases and Immunodeficiency, University Hospital Erlangen, and the University Hospital Jena. Broncho-alveolar lavage was performed using 20 mL of 0.9% sterile saline solution for routine diagnostic purposes. An aliquot was used for the *Pneumocystis* study. The study was approved by the Ethics Committee of the University Hospital Jena (registry number 4511-07/15) and the ethics committees of all collaborating study centers.

BALF samples were fraction-frozen in aliquots either natively or with DMEM-medium and 10–30% FCS, and with or without 5 to 10% DMSO, in a cryopreservation container containing isopropyl alcohol (cooling rate: 1 °C/min) at −80 °C.

### 2.3. Pneumocystis Detection in BALF Samples Using Staining Methods

After shipment of the samples to the research facility, 0.5 mL of BALF was stained with Grocott’s methenamine silver stain (GMS) and DiffQuick stain (DQ) or with MeriFluor *P. jirovecii* IVD direct fluorescence kit (Meridian Diagnostics, Cincinnati, OH, USA, DFT) as previously described [44] to confirm the presence of *P. jirovecii*. Bright field microscopy was used to examine the slides at 400 to 1000-fold magnification (AxioSkop.A1, Zeiss, Jena, Germany) specifically for *P. jirovecii* ascii (GMS) and trophic forms (DQ).

### 2.4. Pneumocystis Detection Using Electron Microscopy (EM)

Pellets made from BALF samples taken from PCP patients and, for comparison, *P. jirovecii* cultures that had been grown on A549 cells were prepared for scanning and transmission EM (protocol in the Supplementary Materials) and viewed with a field-emission scanning EM (Zeiss Merlin VP compact) or a Zeiss EM 902 or a Zeiss Libra120 transmission EM (Zeiss, Germany).

### 2.5. Absolute Quantification of P. jirovecii by mtLSU rRNA Gene Real-Time qPCR

DNA extraction was performed from 0.5 mL of untreated BALF samples using the QIAamp^®^ Blood Mini Kit (Qiagen, Hilden, Germany) according to the manufacturer’s instructions. DNA was stored at −80 °C until further use or analysis with qPCR.

The most important *P. jirovecii* genes known for PCR detection and the primers and probes described for them were aligned and tested for specificity, conservation, and primer binding sites using CLC main software V22.0.2 (Qiagen, Germany) (for detailed procedures, see Supplementary Materials). The qPCRs for the *mtLSU rRNA* gene and the *major surface glycoprotein* gene (*MSG*) were compared in terms of sensitivity.

We then used the Pathogen FAST qPCR kit (Qiagen, Germany) for the detection of a 188 bp fragment of the *Pneumocystis*-specific *mtLSU rRNA* gene by dualplex qPCR (*mtLSU* qPCR). The *mtLSU* qPCR was performed using 5 µL of DNA from BALF samples or cultures in duplicate and four *P. jirovecii* standards developed for our study (Supplementary Materials) and derived from plasmid isolates. This allowed us to calculate the *P. jirovecii* copies/mL BALF based on the standard curve (Supplementary Materials). This method was used for *P. jirovecii* quantification in both BALF samples and *P. jirovecii* cultures.

Furthermore, in order to avoid the propagation of animal-derived *Pneumocystis* spp., which can be present in BALF as contaminants [45], we analyzed all BALF samples using our Pan-*Pneumocystis mtLSU rRNA* gene PCR as described in previous studies [46]. The gene target of this PCR is identical, but it detects a different DNA fragment, permitting the differentiation of all known human and animal-derived *Pneumocystis* species on the basis of specific SNPs.

### 2.6. P. jirovecii Culture from Human BALF Samples

#### 2.6.1. Preparation of *P. jirovecii* Inocula for All Culture Attempts

For the five initial culture studies, we used a single patient’s BALF, while all following experiments were performed using mixtures of 2–4 patients’ BALF samples.

In all initial BALF samples with and without transport medium, *P. jirovecii* copy numbers/mL were analyzed by *P. jirovecii mtLSU* qPCR. After thawing and HIV inactivation, the BALF samples were resuspended thoroughly in the corresponding culture medium by pipetting the BALF-medium mixture through a 21G gauge and adjusting the *P. jirovecii* to the desired concentration; in the main experiments, it was 1–2 × 10^6^ copies/mL medium.

#### 2.6.2. Initial *P. jirovecii* Culture with Isolates from a Single Patient in DMEM-C Medium

The basis for our *P. jirovecii* cultures was the medium for the culture of rat-derived *P. carinii* published by Merali et al. [30] combined with that published by Huang et al. [35]. It contained MEM with Earle’s salts, 20% horse serum, 500 µg/µL SAM, and 80 µg/mL each of PABA, putrescine, ferric pyrophosphate, L-cysteine, L-glutamine, and N-acetyl-D-glucosamine. To prevent bacterial growth, it also contained 500 µg/mL streptomycin and 500 U/mL penicillin.

The five initial *P. jirovecii* cultures were performed as axenic cultures but also in co-culture with A549 cells. For inoculation, *P. jirovecii* isolates from single patients were mixed with DMEM-culture medium (DMEM-C). This DMEM-C contained the supplements described above, but 10% fetal calf serum instead of 20% horse serum and 5 µg/µL amphotericin B for preventing the growth of fungi other than *P. jirovecii*. Cultures were incubated at 37 °C and 5% CO_2_ for 14 days. Sampling was performed on day 0 for estimation of the exact start inoculum and then with additional medium exchanges in the remaining wells every second day.

#### 2.6.3. DMEM-C Medium Optimisation of Axenic Cultures with Mixed *P. jirovecii* Strains

The setup of 29 axenic culture optimization experiments in 24-well plates was identical to the first attempt, but BALF samples from three to four PCP patients were merged in specific volumes to obtain a comparable starting number of *P. jirovecii* organisms in each experiment. Every other day, one well was scraped and 400 µL of medium was analyzed by *mtLSU* qPCR, with the remaining 600 µL used for the detection of microbial contamination and frozen as a backup.

The medium in all remaining wells was removed from each well separately and centrifuged at 4800× *g* for 10 min. The pellet was resuspended in 1 mL of fresh medium in the well from which it had been taken to prevent *Pneumocystis* loss. The experiments were repeated twice for each supplement if *P. jirovecii* growth was visible.

To optimize the axenic *P. jirovecii* culture, different pH values, temperatures, and plate coatings (uncoated and coated with poly-L-lysin or collagen) were tested, as was culture in transwell plates. For comparison, in every experiment, the identical *P. jirovecii* strain was grown in medium without changing the supplements or conditions.

The rate and volume of medium exchange were altered, and varying concentrations of FCS, GlutaMax, carbon sources, and different sugars were tested. These experiments resulted in DMEM-optimized medium 1 (DMEM-O1). During culture, the pH of the medium was measured every 2nd day to evaluate pH level changes due to *P. jirovecii* growth. Due to *P. jirovecii*’s inability to produce some amino acids, we tested various concentrations of essential and non-essential amino acid mixes as well as single amino acids (PABA, SAM, putrescine, copper, and iron sources) to complete a new optimized medium, DMEM-O2.

#### 2.6.4. Long-Term Axenic *P. jirovecii* Flask Cultures with DMEM-O2 Medium

To gain large amounts of pure *P. jirovecii*, 22 axenic cultures were set up in 25 cm^2^ culture flasks using mixed *P. jirovecii* strains and a start inoculum of 1–2 million copies/mL in 10 mL DMEM-O2. The incubation period was 22 to 57 days, and for some cultures, up to 105 days. The only conditions that were altered were the patient’s samples for inoculum and the duration of culture, which in turn increased the final culture volume. The medium was supplemented in a fed-batch manner every 6th day. At these time points, 400 µL of medium were extracted for analysis by *mtLSU* qPCR, and the remaining 600 µL were used for the detection of microbial contamination and frozen as a backup. The culture was then supplemented with 3 mL of fresh DMEM-O2 medium.

#### 2.6.5. DMEM-O2 Medium Optimization of Axenic Cultures with Mixed *P. jirovecii* Strains

To further optimize DMEM-O2 medium, we compared the results of four culture experiments in 24-well plates regarding the supplementation of various combinations and concentrations of non-essential amino acids and metal compounds in 24-well plates. Adding 15 mg/L alanine and cystine and 100 mg/L copper(II)-sulphate to the DMEM-O2 medium resulted in the final medium, DMEM-O3.

#### 2.6.6. Long-Term Axenic *P. jirovecii* Flask Culture with DMEM-O3 Medium and Cluster Size Measurement

Our final DMEM-O3 medium was then used to culture *P. jirovecii* for 70 days (cultures V40 and V41). Fed-batch medium supplementation was carried out every 7th day instead of every 6th day for better handling.

To measure *P. jirovecii* cluster growth, 7.5 mL of the flask culture V40 was centrifuged and dissolved in 4.5 mL of DMEM-O3 medium. A volume of 0.5 mL per well was pipetted into four wells of a LabTek chamber slide (Nunc), while the leftover was quantified by *mtLSU* qPCR. On days 0, 5, 10, and 15 of this experiment, well 4 was thoroughly examined by meandering through it with the CellObserver.Z1 microscope (Zeiss) at 100 to 400-fold magnification. All *P. jirovecii* clusters were photographed and counted, and the visible surface of each cluster was measured in mm^2^. Well 4 was used for each microscopic examination to potentially permit the recognition of specific clusters. Additionally, on every examination day, one well was split into 200 μL aliquots and analyzed (1) by *mtLSU* qPCR for quantification, (2) by LiveDead staining, and (3) by *P. jirovecii* multichannel fluorescence microscopy (Merifluor DFT plus stage-specific marker anti-β-1,3-glucan) with a confocal laser scanning microscope (LSM) as described below.

The experiment was performed as a single replicate and started on day 35. For confirmation of the results, the experiment was repeated in triplicate on day 55 with the same *P. jirovecii* culture.

### 2.7. Quantification of P. jirovecii Growth in Cultures

#### 2.7.1. Microscopic Examination of *P. jirovecii* Clusters in Cultures

The growth of *P. jirovecii* clusters was monitored on every sampling day in all cultures using DIC microscopy (AxioScope A1 or CellObserver.Z1 microscope, Zeiss, Jena, Germany, set to bright field DIC, 200–400-fold magnification). Samples were taken from the same well each time or from the same flask.

To count *Pneumocystis* clusters and measure cluster surfaces in mm^2^ from the chamber slide cultures, a CellObserver.Z1 microscope was used on every 5th day to observe the culture well in its entirety at 200-fold magnification with DIC.

To detect *P. jirovecii* specifically, the MeriFluor *Pneumocystis* IVD fluorescence test kit (Meridian Bioscience, Inc., Cincinnati, OH, USA) was used according to the manufacturer’s instructions. Microscopy was performed at 200-fold to 1000-fold magnification with DIC and fluorescence filters for FITC (ex: 495 nm, em: 517 nm) and DAPI (ex: 358 nm, em: 463 nm) using either a Cellobserver or LSM microscope.

LiveDead staining with calcein AM and ethidium homodimer I (order no. L3224, Thermo Scientific, Bremen, Germany) as carried out on animal-derived *P. carinii* by other research groups [47,48] was performed in order to estimate ascus and spore activity within clusters and to differentiate vital from dead or distorted *P. jirovecii*.

DIC microscopy was combined with *P. jirovecii* DFT, HOECHST 33,342 dye, and the stage-specific marker mouse-anti-1,3-β-glucan monoclonal antibody (Biosupplies Australia, Sydney, Australia), which was stained with an AlexaFluor 546–goat-anti-mouse secondary antibody. *P. jirovecii* organisms were identified using DIC and FITC-labeled *P. jirovecii* antibodies from the commercial IVD kit. The organism nuclei (HOECHST 33342) and *P. jirovecii* ascus cell wall components (mouse-anti-1,3-β-glucan monoclonal antibody and AlexaFluor 546–goat-anti-mouse antibody) were stained and visualized. Analysis was performed with a confocal laser scanning microscope LSM 700 (Zeiss) and argon and HeNe lasers with a 40× objective.

#### 2.7.2. Absolute Quantification of *P. jirovecii* in Cultures Using mtLSU rRNA Gene Real-Time qPCR

The *mtLSU* qPCR for the culture isolates was carried out as for the BALF samples with some modifications:

For *P. jirovecii* quantification, 400 µL of medium from culture plates or flasks was used for DNA extraction and *mtLSU* qPCR. In well plates, the whole well was scraped prior to sampling to loosen adhered *P. jirovecii* clusters. For *P. jirovecii* culture attempts in flasks, it would not have been appropriate to scrape the bottles and thus disturb the formation of *P. jirovecii* clusters. Therefore, the medium in the culture flasks was mixed thoroughly using a 10 mL pipette, and 400 µL of medium was used for qPCR analysis. Due to the limited culture volume in chamber slides, the whole chamber slide well was scraped, and 200 µL of the medium was analyzed by qPCR.

To ensure the comparability of the qPCR results, all copy numbers were given in copies/mL BALF or medium, and the thresholds of the qPCRs of all culture attempts were set to 0.002. The quantification of *P. jirovecii* was calculated by linear extrapolation of the standard curve values.

## 3. Results

### 3.1. Pneumocystis Culture Literature Review

To identify conditions that might be important to the successful culture of *P. jirovecii*, we reviewed 327 papers featuring *Pneumocystis* spp. in vitro cultures with and without feeder cells, including eight papers featuring “*P. carinii* from humans” or “*P. jirovecii*” (Table 1). However, none of the experimental approaches achieved reproducible and stable *P. jirovecii* cultures over a prolonged period.

In early studies, it was assumed that one *Pneumocystis* species could infect different hosts. As a consequence, prior to 2002, it was not clearly stated whether “*P. carinii*” referred to the human or animal *Pneumocystis* species. In 2002, the human-pathogenic form was renamed “*P. jiroveci*”, now “*P. jirovecii*”, and only rat-derived *Pneumocystis* species are now referred to as “*P. carinii*”, while other animal-derived species are named after the host species (Supplementary Materials, Section S1, Table S1). Therefore, we reviewed all the papers available on *Pneumocystis* spp. culture. Even though *P. jirovecii* is very distinct from other fungi and mammal cells, the cell culture conditions and supplements mentioned in the papers for other fungi, as well as methods for growth measurement and characterization that might be applicable to our *Pneumocystis* culture attempts, were nonetheless included in our experiments [49,50].

We identified the following variables as being important in the optimization of our culture: (i) culture with or without feeder cells, (ii) temperature, O_2_ and CO_2_ saturation, (iii) the culture medium used, (iv) the pH of the medium, (v) culture with or without flask agitation, (vi) medium exchange and subculture timing, (vii) *Pneumocystis* start inoculum, (viii) the culture vessels used and their surfaces, (ix) extension of culture time, and (x) supplement addition and optimization.

### 3.2. Initial P. jirovecii Culture with Isolates from a Single Patient in DMEM-C Medium

**Figure 1:**
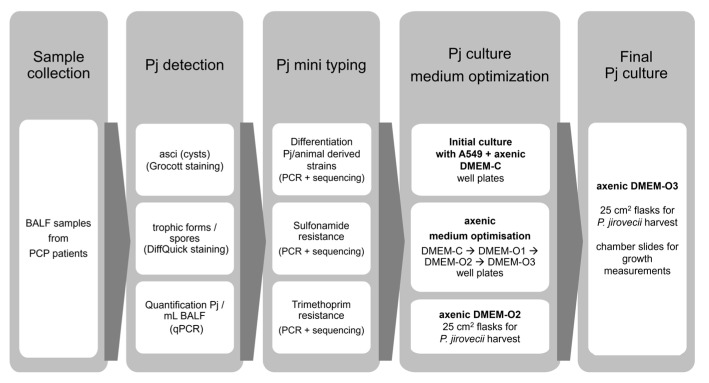
Processing of broncho-alveolar lavage fluid (BALF) samples from patients with *Pneumocystis* pneumonia (PCP). The results of the optimization experiments with co-culture on A549 cells are presented in the Supplementary Materials. Pj—*P. jirovecii*.

**Figure 2:**
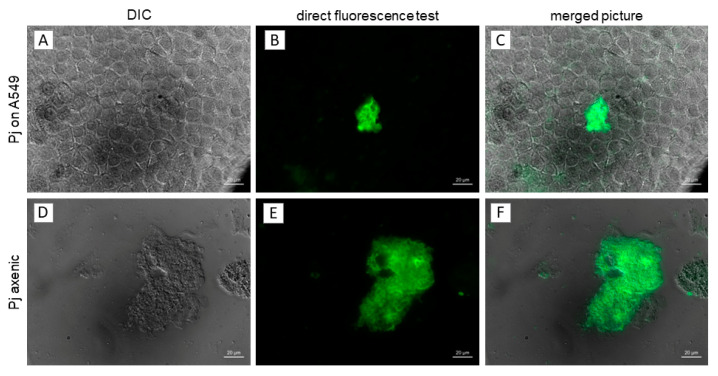
*P. jirovecii* clusters in cultures using A549 feeder cells and in axenic *P. jirovecii* cultures (patient 5, 14 days after start of culture). (**A**,**D**)—DIC, (**B**,**E**)—FITC channel after staining with commercial direct fluorescence test; (**C**,**F**)—merged photos. Clusters of *P. jirovecii* can be seen clearly in the axenic culture (**D**–**F**), while small *P. jirovecii* clusters tend to disappear behind the layer of A549 cells (**A**) and are only visible when stained with DFT (**B**,**C**). The larger clusters that can be seen in later stages of *P. jirovecii* culture are also clearly visible in the co-cultures with A549 cells. bar = 50 µm.

Our first experiments for cultivating *P. jirovecii* were based on the published animal-*Pneumocystis* cultures, and we used A549 lung carcinoma cells as feeder cells to supplement essential nutrients. In parallel, we cultured the identical *P. jirovecii* strains in an axenic attempt (Figure 1 and Figure 2).

DMEM has been shown to support the growth of rat-derived *P. carinii* on A549 cell monolayers [13,29,51]. Therefore, we started our cultural attempt with a medium based on the *P. carinii* media published by Merali et al. and Cushion et al. [30,38], but with some modifications: Our initial culture medium, which we called “DMEM-culture” (DMEM-C), contained 10% fetal calf serum (FCS) instead of horse serum, 3.7 g/L sodium bicarbonate, 500 µg/mL SAM, 0.4 g/L glucose, 80 µg/mL each of L-glutamine, L-cysteine, and PABA, 200 mg/L streptomycin, 200,000 U/L penicillin, and we added 5 mg/L amphotericin B to prevent the growth of fungi other than *Pneumocystis* (Table 2).

**Figure 3:**
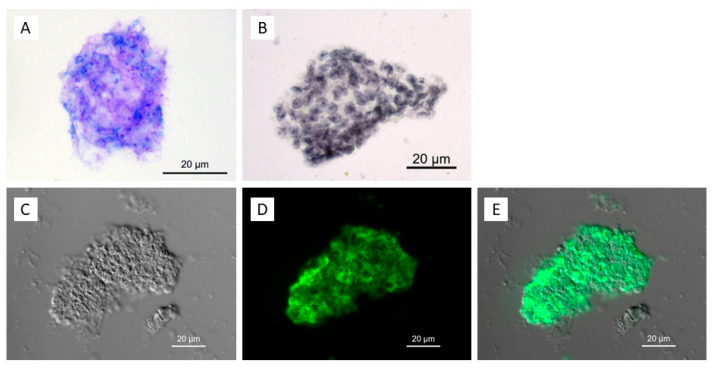
*P. jirovecii* trophic forms and asci (cysts) in initial BALF samples (**A**): DiffQuick stain showing trophic forms, some with visible nuclei (BALF from patient 1); (**B**): Grocott methenamine silver stain (GMS) with asci (BALF from patient 1). GMS stains the walls of intermediate and mature asci in gray, making it easy to detect asci even at low magnifications. However, no trophic forms or spores can be detected using this method. DiffQuick or Giemsa stain permit the detection of trophic forms and unripe asci. Therefore, the methods should be used in combination. C-E: large cluster of trophic forms and developing asci examined with DIC microscopy (**C**) and stained with DFT (**D**). Photos (**C**,**D**) are merged in (**E**) (BALF from patient 5).

Prior to the culture attempt, the patient’s BALF was analyzed using *P. jirovecii*-specific methods (Figure 1), such as staining to detect asci and trophic forms (Figure 3). This was followed by quantification of *P. jirovecii* in the BALF via *mtLSU rRNA* gene qPCR (Supplementary Materials, Figure S4) to adjust the inoculum of the cultures. The concentration of the *P. jirovecii* culture inoculum was 1.85 × 10^6^ ± 1.56 × 10^6^ copies/mL in the axenic cultures.

**Figure 4:**
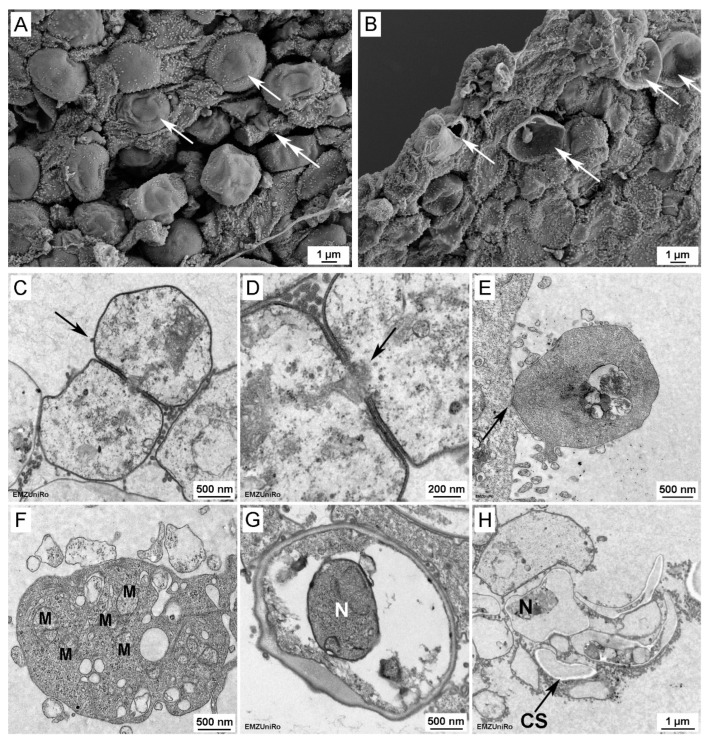
(**A**,**B**): REM pictures of *P. jirovecii* (**A**) asci with microdots (arrows) and trophic form (double arrow) and (**B**) empty and ruptured asci (arrow), partially with spores (double arrow). In both REM pictures (**A**,**B**), filopodia (rough structures visible on the surface of most asci) are clearly visible. (**C**) Filopodia are also visible in TEM pictures (black arrow). (**C**,**D**) Direct interaction between trophic forms with membrane blurring (large black arrow, TEM, (**D**)) = detail of (**C**); (**E**) interaction between trophic forms and host cells (A549) (arrow). (**F**) and mitochondria (M) are visible in the trophic forms, but the number of mitochondria per cell cannot be counted with TEM. Structures in *P. jirovecii* asci (**G**) and trophic forms (**H**): nuclei (N) and some other organelle residues are visible, but some structures were destroyed by the fixation process, as indicated by white structures associated with shrinking of the cytoplasm (CS).

On the first culture days, only small *P. jirovecii* clusters or single cells were visible with DIC microscopy. Between day 6 and day 8, the first clusters consisting of up to 50 *P. jirovecii* trophic forms could be observed (Figure 2). In cultures older than 14 days, larger clusters with hundreds to thousands of trophic forms per cluster were present, while ascii were visible only in isolated cases. In cultures with A549 feeder cells, a few *P. jirovecii* cells were loosely attached to the A549 cells (Figure 4) and to each other, partially with membrane blurring between the spores (Figure 4C,D), as visible by electron microscopy. In axenic cultures, clusters with trophic forms floated without significant attachment to the culture well surface. This initial axenic culture attempt showed a maximum 9.1-fold increase in *P. jirovecii*.

**Table 1:** Successful *Pneumocystis* culture systems. (a)—*P. jirovecii* culture attempts. (b)—cultures with animal-derived *Pneumocystis* spp. L2—Lung epithelial-like cells, CEL—embryonic chicken epithelial lung cells, WI-38 human lung fibroblast cell line, MRC-5—human fetal lung cell line, fibroblast cell lines, LLC-MK-2 Rhesus monkey kidney epithelial cells, FL—human amnion cells shown to be a HeLa derivative, Vero—kidney epithelial cells from African green monkey.

Year Pub-lished	*Pneumocystis* Strain and Host Species ^1^	Feeder Cells	Medium	Supplements and Concentration	Culture Conditions	Duration	Start Inoculum and *Pneumocystis* Growth	Non-Successful Experiments /Cell Lines	References
**(a) *P. jirovecii* culture attempts**									
1977	*P. carinii* (human = *P. jirovecii*)	CEL	Medium 199	FBS, human serum or no serum	37 °C	12 days with passages every 3rd day (Pc) or 2 passages every 7th day (*P. jirovecii*)	Inoculum: 9.1 × 10^4^ cysts or unquantified number of trophic forms Growth: Murine Pc: 100-fold (2.7 × 10^5^ to 2.3 × 10^7^) *P. jirovecii*: 10-fold (5 × 10^3^ to 7.6 × 10^4^)	Less than 1 × 10^3^ cysts/ 12.4 × 10^7^ cells resulted in culture failure, No growth in any cell-free media	[18]
1984	*P. carinii* from human lung and BALF samples (=*P. jirovecii*)	A549	DMEM	10% FCS	37 °C 25 cm^2^ and 75 cm^2^ flasks	7 days, 14 days	Inoculum: 1 mL pellet in PBS from mined human lungs or BALF to 9 mL medium Growth: 8-fold increase of trophic forms after one week, 10-fold increase after 14 days	Growth only in one of ten human specimens (lung biopsy)	[13]
1989 1990	*P. carinii* of human origin (=*P. jirovecii*)	A549 Vero	RPMI 1640 DMEM	0 or 10% FCS Penicillin 200 U/mL Streptomycin 200 µg/mL Miconazole 0.5 µg/mL L-glutamine 200 mM HEPES 25 mM	37 °C 5 or 10% CO_2_ Some cultures: irradiation of A549 prior to infection 25 cm^2^ flasks chamber slides	2–6 days	Inoculum: 0.1 to 0.5 mL BALF sediment to 5 mL medium Growth: optimal with A549 incubated at 37 °C and 10% FCS plus all additives; cyst density larger in irradiated A549 cultures	-	[17]
1997	*P. carinii* (human = *P. jirovecii*)	A549	F12	10% FCS Penicillin 200 U/mL Streptomycin 200 mg/mL Miconazole 0.5 mg/mL	37 °C	4 days	Inoculum: from human lung, *P. jirovecii* number not mentioned Growth: only decline of *P. jirovecii* within 48–72 h, after 96 h no *P. jirovecii* detectable	No success	[14]
1999	*P. carinii* (human = *P. jirovecii*)	Axenic	MEM with Earle’s salt	20% horse serum 500 mg/mL *S*-adenosyl-L-methionine sulphate 80 mg/mL each of the following *p*-aminobenzoic acid, putrescine, ferric pyrophosphate, L-cysteine, L-glutamine, and *N*-acetyl-D-glucosamine 500 U/mL penicillin 500 µg/mL streptomycin	31 °C pH 8.8–9 Transwell inserts	50 days with 6 sub-cultures (weekly splitting)	Inoculum: 2 to 3 × 10^7^ organisms/mL Pc doubling time 19–44 h Cultures with small inoculum (2 × 10^6^) had faster growth 920-fold increase in 7 days (doubling time: 19 h) Growth of *P. jirovecii* in preliminary experiments!	No growth after dilutions to only 5–10 clusters/well	[30]
2018	*P. jirovecii*	CuFi-8 cells EpiAirway cells	Hams F-12	No further supplements	37 °C	2–5 days	Inoculum: 10–150 µL *P. jirovecii*-positive BALF Growth: *P. jirovecii* copy number max. increase from 1 × 10^2^ to 1 × 10^7^ (measured by a *mtLSU* gene qPCR)	Cytopathic effect of *P. jirovecii* destroys CuFi-8 and EpiAirway cells quickly Commercially non-available CuFi-8 cells *P. jirovecii* culture was NOT reproducible by others	[15] reply: [16] reply: [19]
2018	*P. jirovecii*	CuFi-8 cells	Hams F-12	No further supplements	37 °C	min. 5 days	Inoculum: not mentioned Growth: no growth observed	*P. jirovecii*-positive BALF of 10 patients did not reveal any *P. jirovecii* growth (8 of 10 had no *P. jirovecii* copies after day 5 of culture), 2 of 10 rapid decline	[16] ^2^ reply: [19]
**(b) Important animal-derived *Pneumocystis* spp. culture attempts (shortened, see Supplementary Materials** **).**									
1977	*P. carinii* (rat)	CEL	Medium 199	FBS human serum or no serum	37 °C	12 days with 4 passages every 3rd day (Pc) or 2 passages every 7th day (*P. jirovecii*)	Inoculum: 9.1 × 10^4^ cysts or unquantified number of trophozoites Growth: Murine Pc: 100-fold (2.7 × 10^5^ to 2.3 × 10^7^) Human *P. jirovecii*: 10-fold (5 × 10^3^ to 7.6 × 10^4^)	Less than 1 × 10^3^ *P. carinii* cysts/12.4 × l0^7^ cells resulted in culture failure, No growth in any cell-free media	[18]
1990	*P. carinii* (rat)	Axenic	DMEM and others ^3^	DMEM medium: Penicillin-streptomycin Amphotericin B NPG medium (variable supplements and conditions): FCS: 10 or 20% 1% neopeptone 0.2% N-acetylglucosamine Cysteine, palmitate, oleate, linoleate, ergosterol or cholesterol 0.025 to 0.1 mg/L, Hemin 10.0 mg/mL	Solid agars: pH 4.0 and 7.0 NPG medium: pH 4.0 to 8.0 (optimal: pH 4.0) 4-25-31-35-37-41 °C (optimal: 35-37 °C)	6–7 days	Inoculum: 2.5 × 10^6^ to 1 × 10^8^/mL, optimal inoculum: 2.5 × 10^6^ to 1 × 10^7^ nuclei/mL Optimal growth: 8 to 10-fold increase in NPG medium containing 1% neopeptone (wt/vol) and 0.2% (wt/vol) N-acetyl-D-glucosamine (GlcNAc) at a pH of 4.0 over 1 week Subculture: - without dilution of organisms: 2-fold increase after 7 days - with dilution to 1 × 10^7^ organisms/mL: 7-fold increase after 7 days	No growth enhancement: - NPG medium with sugars other than GlcNAc, including glucose, another amino sugar, N-acetyl galactosamine, - NPG medium with fatty acids or sterols - Neopeptone alone - reduced oxygen tension	[38]
1995 1999	*P. carinii* (rat)	L2 Axenic	DMEM	10% FCS antibiotics (penicillin/streptomycin)	37 °C 5% CO_2_	4 days 7 days	Inoculum (4-day culture): ~2 × 10^6^ (/mL?) Inoculum (7-day culture): 1 × 10^7^/mL	Rat-derived Pc in vitro culture: no growth after day 4 in cultures with feeder cells, after day 2 in axenic cultures	[34,52]
1999	*P. carinii* (rat)	Axenic	MEM with Earle’s salt	20% horse serum 500 mg/mL *S*-adenosyl- L-methionine sulphate 80 mg/mL each of the following *p*-aminobenzoic acid, putrescine, ferric pyrophosphate, L-cysteine, L-glutamine, and *N*-acetyl-D-glucosamine, 500 U/mL penicillin, 500 µg/mL streptomycin	31 °C pH 8.8–9 Transwell inserts	50 days with 6 sub-cultures (weekly splitting)	Inoculum: 2 to 30 × 10^6^ organisms/mL Pc doubling time 19–44 h Cultures with small inoculum (2 × 10^6^) had faster growth 920-fold increase in 7 days with a doubling time of 19 h	no growth was observed after dilutions to only 5–10 clusters/well	[30]
2000	*P. carinii* (rat)	Axenic	MEM with Earle’s salts	20% horse serum Putrescine 80 µg/mL Ferric pyrophosphate 80 µg/mL L-cysteine 80 µg/mL Glutamine 80 µg/mL s-adenosyl-L-methionine (SAM) 500 µM	31 °C Transwell, collagen-coated	21 (27?) days	Inoculum: 3 × 10^6^ cells/mL Growth: 24 × 10^7^ nuclei per Giemsa-stained slide (culture volume/slide not mentioned) on day 21 of culture	Decline of Pc in medium without SAM after 15 days	[40]
2001	*P. carinii* (rat)	Axenic? No cells Mentioned	RPMI-1640	20% FCS 1× MEM vitamins 1× NEAA, L-glutamine (conc. not mentioned) 100 IU penicillin 100 µg/mL streptomycin	Temp. + CO_2_/O_2_ not mentioned Every 24 h agitation of samples, 50% medium exchange	7 (9?) days	Inoculum: 1 × 10^8^ to 2 × 10^8^ nuclei/mL Growth: Slightly increase in media without pentamidine until day 2 (9.36 × 10^7^ to 9.93 × 10^7^ nuclei)	Decrease of Pc nuclei in media with and without pentamidine over the whole culture period of 7 days	[53]
1978	*P. carinii* (rat)	VERO	MEM Medium 199	2% FBS	37 °C	7 days Passage every 24 h	Inoculum: 1.3 × 10^5^ to 8.5 × 10^5^ cysts/culture of 1–2 mL Growth: Max. 11-fold increase in cysts 72h post inoculation	Little growth (3-fold increase) in owl monkey kidney, baby hamster kidney, and AV-3 cell cultures, and no growth in WI-38 cells and secondary chicken fibroblast cultures Maximum 3–4 passages, then decline	[54]
1979	*P. carinii* (rat)	WI-38 MRC-5	Eagle medium	2–10% FCS 50 µg/mL streptomycin 100 U/mL penicillin G potassium 10 U/mL nystatin	35 °C No CO_2_	10 days Subcultures from 4–5 days old cultures	Inoculum: 1 mL supernatant derived from 1 cm^3^ ground rat lung; number of *Pneumocystis* in inoculum not measured Growth: 117.9 × 10^6^	High variances between growth of rat isolates even if the rat lung used for culture had high organism loads	[55]
1985	*P. carinii* (rat)	A549 WI-38	A549: DMEM WI-38: HMEM	25 mM HEPES 20% HyClone FBS/serum (rat, chicken, swine, human, horse) from several companies 1000 U penicillin 1000 µg streptomycin 0.5 µg amphotericin B NCTC vitamin mixture 107 formula no. 78-0776 or no vitamins	Room temp: 30-35-37-41 °C (optimal: 30-37 °C for Pc on A549 cells); Stationary or rocking 6 rpm; With/without 5%CO_2_	20 days	Inoculum: Optimal: 1 × 10^6^ organisms/mL Maximal: 1 × 10^8^ organisms/mL Minimum: 1 × 10^5^ organisms/mL Growth: in both cell lines Subculture/passaging: 3 successful passages 7 days after start with 12-fold organism increase, or 14 days after start resulting in a steady-state or 21 days after start with 4-fold organism increase	Inhibitory effect (in both cell lines): - temperature 41 °C - 0.05% saponin - rocking/moving the cultures Source of serum (rat, swine, human) or temperature led to a decrease in growth in VA13 cells	[29]
1985	*P. carinii* (rat)	WI-38 MRC-5	Eagle medium	2–10% FCS 50 µg/mL streptomycin 100 U/mL penicillin G potassium 10 U/mL nystatin	5% O_2_ 5–10% CO_2_ 35 °C	10 days	Inoculum: 1 mL supernatant derived from 1 cm^3^ ground rat lung; number of *Pneumocystis* inoculum not measured Growth: peak at day 6 max organism number (peak day) 1.8 to 17.5 × 10^6^ in *P. carinii* on WI-38 cells and 0.7 to 16.1 × 10^6^ in *P. carinii* on MRC-5 cells more constant growth on WI-38 cells	2 to 3 passages/harvests possible Growth decreased in subcultures	[56]
1993	*P. carinii* (rat)	HEL (A549 and L-132 gave no good results)	Eagle MEM (DMEM, RPMI-1640, medium 199 gave no good results)	10% FCS Antibiotics (penicillin, streptomycin, amphotericin B, no concentration mentioned) Medium exchange every 3rd day Passaging every 3–5 days	5% CO_2_ 37 °C	42 days	Inoculum: 1 to 3 × 10^7^ *P. carinii*/mL in 24-well tissue culture plates Growth: peak at day 6 to 9 or 10, but after that decline	Cell-bound and supernatant *P. carinii* were counted separately (30% were adherent to cells) Infection of rats after 41 days of in vitro culture was successful	[39]
2009 2011	*P. carinii* (rat)	axenic	RPMI-1640	20% calf serum penicillin (200 U/mL) streptomycin (200 µg/mL) amphotericin B (0.5 µg/mL) vancomycin (5 µg/mL) S-adenosyl-L-methionine farnesol vitamins and amino acids as described before PET track-etched membrane cell culture inserts Transwell inserts Millicell-CM hydrophilic PTFE membranes Millicell-HA insert	36 °C 5% CO_2_	7–14 (21) days	Inoculum: 1 × 10^6^ to 1 × 10^8^ Growth: Less extensive biofilm formation than *P. murina* from mice (thickness of <15 µm)	S-adenosyl-L-methionine led to a dramatic decrease of viability within 24 h in *P. carinii* and *P. murina* Farnesol led to decreased viability in *P. carinii*	[31,43]
2006	*P. carinii* (rat)	axenic	RPMI-1640	20% calf serum 1× MEM vitamins non-essential amino acids (conc. not mentioned), L-glutamine (conc. not mentioned), 100 IU penicillin, and 100 mg/mL streptomycin Start inoculum: 5 × 10^7^ organisms/mL	Standard: 21% O_2_ and 5% CO_2_ microaerophilic:10–15% O_2_, 7–15% CO_2_ anaerobic: <1% O_2_, 10% CO_2_	7 days	Inoculum: 5 × 10^7^ *P. carinii*/mL Growth: ATP measurement only: 15,000 RLU under microaerophilic conditions vs 3000 RLU under standard conditions after 7 days	Anaerobic conditions: decline after the first day	[57]
2013	*P. carinii* (rat)	axenic	DMEM	10% FBS 100 U/mL penicillin 100 μg/mL streptomycin	37 °C 5% mio	2–4 days	Inoculum: 0.75 × 10^4^ *P. carinii*/mL or 2.5 × 10^5^ *P. carinii*/mL Growth: from 6 × 10^5^ to 10 × 10^5^ organisms within 40 h, then decline	Decline of *Pneumocystis* life stages after 2 days in all cultures	[42]

^1^ as stated in the article, ^2^ was not used for design of our culture attempts, ^3^ other media used: YEPD broth + agar, YM broth, wort broth + agar, BHI broth + agar, Sabouraud dextrose broth + agar, modified Sabouraud dextrose broth + agar, Vogel & Johnson agar, NPG medium.

**Table 2:** Composition of the initial *P. jirovecii* DMEM culture medium (DMEM-C) and conditions for the culture of *P. jirovecii* strains from one patient with and without A549 cells.

**Culture Conditions:**	
37 °C 5% CO_2_ 1 mL/well in 6-well plates Duration: 14 days Medium exchange and sampling every day	
**Medium Ingredients:**	
DMEM low glucose	
Sodium bicarbonate	3.7 g/L
Amphotericin B	5 µg/mL
Penicillin G	200 U/mL
Streptomycin	200 µg/mL
FCS	10%
S-adenosyl-L methionine	500 µg/mL
Para-aminobenzoic acid	80 µg/mL
L-glutamine	80 µg/mL

**Figure 5:**
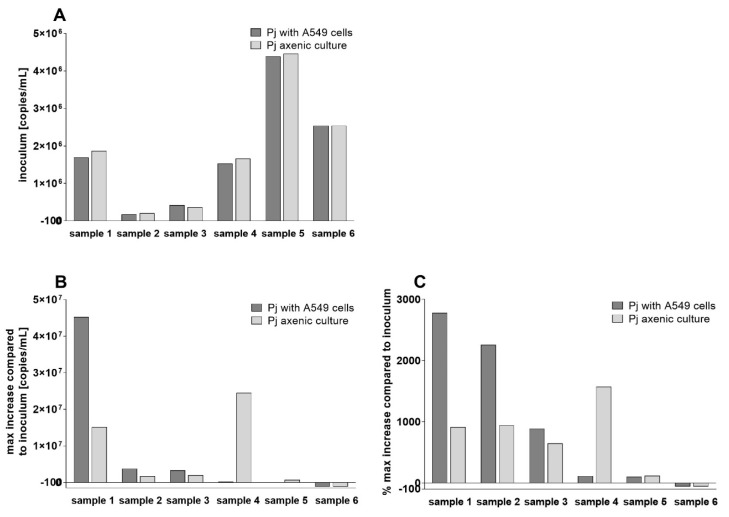
*P. jirovecii* culture with A549 cells and axenic culture without feeder cells using *P. jirovecii* from a single patient’s BALF samples. The inoculum number (*mtLSU* qPCR copies/mL BALF) was equal for cultures with and without A549 cells but differed between the patient samples (**A**). The maximal increase in *P. jirovecii mtLSU* copy numbers during culture was 4.5 × 10^7^ copies/mL culture, or 2770% of the *P. jirovecii* inoculum in sample 1, cultured on A549 cells (**B**,**C**). All cultures with a *P. jirovecii* inoculum below 1 × 10^6^ copies/mL or above 4 × 10^6^ copies/mL showed slow growth (isolates of patients 2 and 3), steady state (isolate of patient 5), or a decline (isolate of patient 6) in *P. jirovecii* copy numbers. This was observed independently in cultures using A549 cells and in axenic cultures.

*P. jirovecii* growth in DMEM-C did not follow a linear growth curve but instead exhibited peaks associated with intermittent growth (Supplementary Materials, Figure S5). Three of five axenic cultures showed a microscopic increase in *P. jirovecii* clusters and *P. jirovecii* copies/mL after 14 days (Figure 5). A steady state or decline in *P. jirovecii* was seen in two other axenic cultures, but these did not grow on A549 cells either. All further culture optimization experiments with A549 cells can be found in the Supplementary Materials.

**Conclusions:** A *P. jirovecii* culture in DMEM-C medium developed from culture media for animal-derived *Pneumocystis* strains was successful. *P. jirovecii* strains from individual patients grew in cultures with and without feeder cells. Culture on feeder cells resulted in faster and more stable growth than without feeder cells, but the A549 feeder cells hampered all further analyses of the organism, including next-generation sequencing. A culture that produced a higher harvest without feeder cells was required for the next step.

### 3.3. DMEM-C Medium Optimization of Axenic Cultures with Mixed P. jirovecii Strains

In order to improve the *P. jirovecii* yield in axenic cultures, in the next step of our experiment, we optimized the following parameters: (i) physical parameters: strains, temperature, culture vessel surfaces and systems, medium pH; (ii) medium supplements in concentration series: FCS, mono- and disaccharides (glucose, maltose, galactose, sucrose), essential and non-essential amino acid mixtures, vitamins, para-aminobenzoic acid (PABA), and S-adenosyl-L-methionine (SAM). PABA and SAM had been described as essentials for rat-derived *P. carinii* cultures [30], and physical parameters such as protein, sugar, and amino acid concentrations clearly needed optimizing for organisms that had previously been considered uncultivable. We oriented the experiments towards culture supplements used for animal-pathogenic *Pneumocystis* (Table 1, Supplementary Materials Table S3) in this first optimisation step.

As in the first culture experiments, only isolated *P. jirovecii* cells, if any, were visible with DIC microscopy on the first few days of culture.

Combining *P. jirovecii* strains from up to four different BALFs resulted in more stable growth than that of a single patient. In our experiments, a pH of 8.0 was optimal for all cultures, and no pH alteration during *P. jirovecii* growth was seen (Table 3). A medium pH above 8.0, as described in the axenic rat *Pneumocystis* cultures [30], was not optimal for *P. jirovecii*.

In the literature, temperatures between 31 and 40 °C were used for animal *Pneumocystis* cultures [29,56]. In our experiments, temperatures ranging from 31 to 37 °C were tested, corresponding to normal lung temperatures and the growth conditions of other fungi. Optimal *P. jirovecii* growth was observed at 37 °C (Table 3).

#### 3.3.1. The Addition of Mono- and Disaccharides to the DMEM-C Medium Increased the Growth of *P. jirovecii* under Cell-Free Conditions

Most cultures for rat-derived *P. carinii* used media with low sugar concentrations or stated that glucose did not enhance *P. carinii* growth [38]. As a result, we started our initial cultures with DMEM-C, a low-glucose medium. Other fungi needed high sugar concentrations; therefore, we tested mono- and disaccharides in various concentrations as carbon sources.

**Figure 6:**
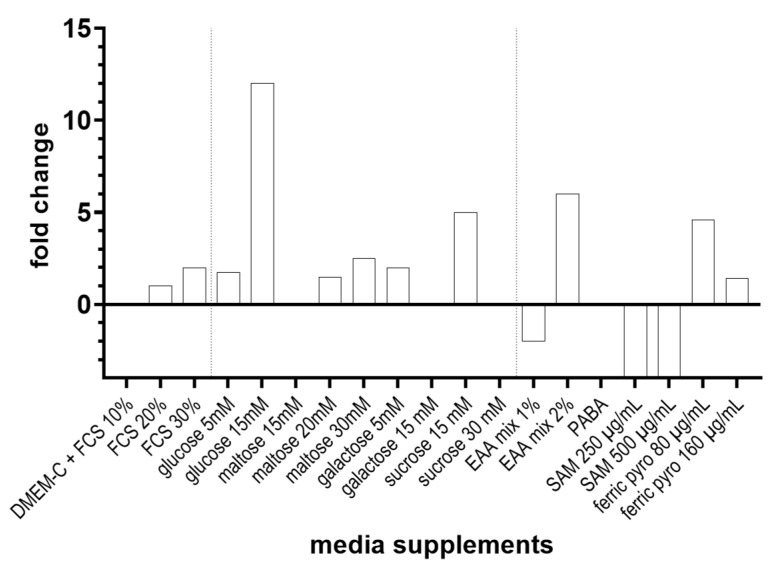
Culture supplements and concentrations tested for improving the growth of *P. jirovecii* with DMEM-C medium in axenic culture (for culture optimization on A549 cells, see Supplementary Materials). *P. jirovecii* was grown in 24-well plates for 10–14 days and quantified by qPCR. Growth was examined microscopically every 2nd day. EAA—essential amino acid mix 50×, NEAA—non-essential amino acid mix; SAM—S-adenosyl-L-methionine, PABA—para aminobenzoic acid; ferric pyro—ferric pyrophosphate.

Glucose 15 mM was most effective, leading to a 12-fold increase in *P. jirovecii* after six days (Figure 6). A less pronounced and more variable 2 to 5-fold increase in *P. jirovecii* growth was also seen during the first eight days of culture after supplementation of either 15 mM sucrose, 5 mM galactose, or 30 mM maltose (Figure 6). The number of *P. jirovecii* organisms observed on day two of culture was increased by all sugars compared to the number observed without the addition of these sugars to the media.

#### 3.3.2. Addition of Micronutrients to the Culture Medium Promoted the Growth of *P. jirovecii*

In the next step, we investigated the growth rate of *P. jirovecii* in different concentrations of FCS and other compounds that had been mentioned as being essential for animal *Pneumocystis* cultures. Axenic *P. jirovecii* culture with 30% FCS showed a 2-fold increase in *P. jirovecii* by day 10, while 10% FCS showed a decline in *P. jirovecii* to 25% of the inoculated amount. Culture at pH 9, or 31 °C, brought about a strong decline in *P. jirovecii* organisms. Ferric pyrophosphate (80 µg/mL), which had no effect on *P. jirovecii* cultures on A549 cells, caused a 4-fold increase in *P. jirovecii* after 12 days of culture. GlutaMax and 25 mM HEPES only showed stabilizing effects. Para-aminobenzoic acid (PABA), S-adenosyl-L-methionine (SAM), and putrescine were used in the initial culture media for *P. carinii*, which was assumed to be unable to synthesize these substances [30,40,58,59,60,61], but SAM and PABA did not increase *P. jirovecii* growth in either culture system (Figure 6). All other supplements and concentrations tested only had a weak or no effect on *P. jirovecii* cultures.

In a further step, we investigated whether the addition of the micronutrients copper(II) sulphate pentahydrate, ferrous sulphate heptahydrate, or ferric pyrophosphate, the latter two iron derivatives, had an effect on the growth of *P. jirovecii*.

**Figure 7:**
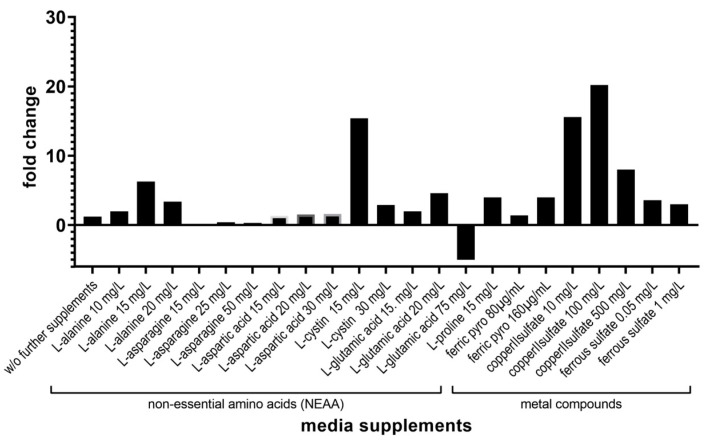
Culture supplements and concentrations were tested for their effect on the growth of *P. jirovecii* in DMEM-O2 medium (with 30% FCS, 25 mM HEPES, and 4 mM GlutaMax instead of glutamine) in axenic culture only. *P. jirovecii* was grown in 24-well plates for 10–14 days. *P. jirovecii* growth was quantified by qPCR. Growth was examined microscopically every 2nd day. ferric pyro—ferric pyrophosphate.

The addition of iron compounds such as ferrous sulphate heptahydrate and ferric pyrophosphate increased the *P. jirovecii* copy number in the cell-free culture 3.1- to 4-fold by day 10 (Figure 7). Based on these results, we changed the formulation of the DMEM-C medium by adding 20% FCS, 20 mL/L Glutamax (L-alanyl-L-glutamine), 3.4 mg/mL glucose, 80 µg/mL ferric pyrophosphate, 100 µg/mL 2-mercaptoethanol, and HEPES 12.6 µg/mL and called it DMEM-O1 medium (Table 4). We used this DMEM-O1 medium for our next cultivation experiments.

#### 3.3.3. Supplementing the DMEM-O1 Culture Medium with Different Amino Acids and Varying Concentrations of FCS Showed Differential Effects on the Growth of *P. jirovecii*

Adding 100 mg/L copper(II) sulphate pentahydrate to the DMEM-O1 medium resulted in a 20.2-fold increase in *P. jirovecii* copy number and microscopically visible *P. jirovecii* growth ten days after the start of cultivation (Figure 7).

**Figure 8:**
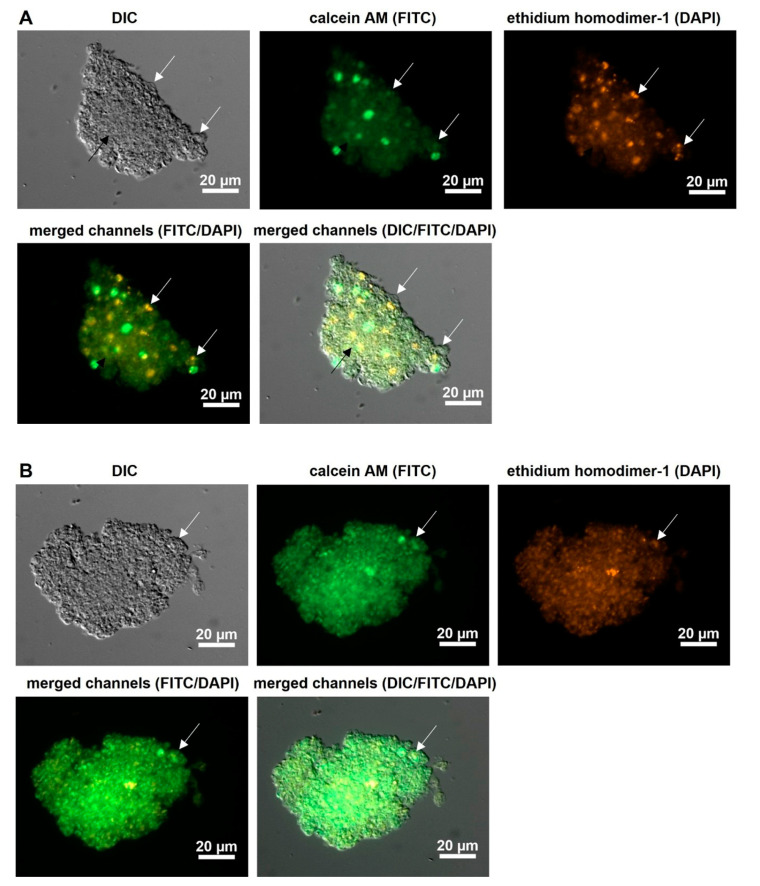
*P. jirovecii* cluster from in vitro culture in DMEM-O2 medium, axenic culture day 10, stained with *LiveDead* staining. (**A**): *P. jirovecii* clusters without L-alanine showed vital (bright green) and potentially inactive/dead *P. jirovecii* (orange). The trophic forms were located mainly in the inner parts of the huge clusters (black arrow), while some developing asci were located in the outer parts (white arrows). (**B**): *P. jirovecii* clusters with 20 mg/L L-alanine showed many vital (bright green) and only a few potentially inactive/dead *P. jirovecii* (orange). Compared to A, the cluster was the same size, but the *P. jirovecii* organisms inside the cluster seemed to be better nourished than those without additional L-alanine and only exhibited a few ascii (arrow).

Previous authors have reported that the addition of various amino acids has a positive effect on *Pneumocystis* growth [29]. Therefore, we added the amino acids 15 mg/L proline, 20 mg/L glutamic acid, 15 mg/L alanine, and 15 mg/L cystine to the DMEM-O1 medium. After inoculation with *P. jirovecii*, a 4-fold, 4.6-fold, 6.3-fold, and 12.4-fold increase in *P. jirovecii* was seen after 14 days. With the addition of alanine, *P. jirovecii* was observed to be mainly vital, with only a few potentially inactive organisms inside the clusters (Figure 8). Supplementation of asparagine had an inhibitory effect, bringing about a strong decline in *P. jirovecii* to 10–26% of the inoculum.

These medium optimization steps resulted in us adding a mixture of sugars (glucose, galactose, sucrose, and maltose), amino acids, and ferric pyrophosphate to the next medium, DMEM-O2 (Table 5).

**Conclusions:** Copper, iron compounds, sugars, and higher doses of amino acids improved *P. jirovecii* growth in axenic cultures. All supplements increasing or stabilizing *P. jirovecii* growth in the DMEM-O1 medium were combined in the new medium, DMEM-O2.

### 3.4. Long-Term Axenic P. jirovecii Flask Cultures with DMEM-O2 Medium

Using DMEM-O2 medium, we analyzed the optimal *P. jirovecii* organism inoculum, culture length, and medium supplementation time points. For this, we cultured *P. jirovecii* in 24 axenic flask cultures (V1 to V24) to produce larger organism numbers.

*P. jirovecii* inoculum in these 24 culture attempts ranged from 146,948 to 12.05 × 10^6^ copies/mL, with a median of 2.0 × 10^6^ copies/mL (2.84 × 10^6^ ± 2.46 × 10^6^ copies/mL, mean ± SD). The largest increase in medium volume was 52% over 52 days. Cell-free culture upscale carried out by increasing culture volume and increasing the duration of cultivation led to a maximum increase in *P. jirovecii* to 1.18 × 10^9^ *P. jirovecii* copies/culture at a minimum doubling time of 6.9 days.

**Figure 9:**
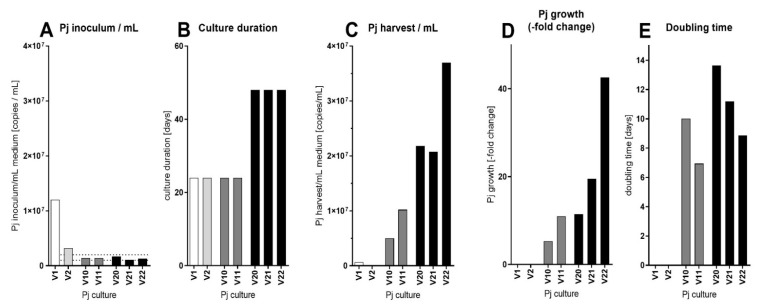
Growth of *P. jirovecii* in five of 24 axenic long-term flask cultures in DMEM-O2 medium (for complete graphs of all flask cultures, see Figure S8). The starting volume was 10 mL/flask, and all cultures were incubated at 37 °C and 5% CO_2_ for 22 to 57 days. Culture conditions, except the three factors (1) patient samples for inoculum, (2) duration of culture, and (3) final culture volume, were not altered. Samples for qPCR analysis were taken, and volumes of fresh medium were added on top of the old medium to dilute the growing *P. jirovecii* to appropriate densities in the flask (fed-batch culture). As seen after the first five cultures, *P. jirovecii* growth ceased when the starting inoculum was too dense (A). A start inoculum of 1 to 2 × 10^6^ copies/mL culture medium volume was optimal, while all cultures with a higher or lower inoculum showed decreasing *P. jirovecii* copy numbers/mL medium. The culture duration was increased from 24 days to up to 57 days with an optimum of 48 days (B). These alterations increased the *P. jirovecii* harvest from 0.6 × 10^6^ *P. jirovecii* copies/mL medium in V1 to 3.7 × 10^7^ *P. jirovecii* copies/mL medium in V22 (C), which was a 42.6-fold increase in this culture (D). The doubling time ranged from 6.94 days in V11 to 13.6 days in V20, with a mean doubling time of 10.1 days in the optimal cultures (E). A medium increase of up to 60% during culture and a duration of 48 days resulted in the largest increase of *P. jirovecii*, with an optimum doubling time of 6.9 to 8.8 days and a 42.6-fold *P. jirovecii* increase.

The fastest *P. jirovecii* replication was seen in culture V11 at 6.9 days. Nevertheless, the highest *P. jirovecii* yield was achieved in axenic flask culture V22 with a doubling time of 8.8 days (most important flask culture experiments: Figure 9, all 24 flask cultures: Supplementary Materials Section S11, Figure S8) under the following conditions: DMEM-O2 medium for 48 days at 37 °C and 5% CO_2_ with a *P. jirovecii* start inoculum of 1.3 × 10^6^ copies/mL culture medium, growth-adjusted fed-batch of 2 mL medium every 8th day, splitting into two flasks when necessary. The number of harvested *P. jirovecii* in culture V22 was 8.7 × 10^6^ ± 12.0 × 10^6^ copies/mL and 282.5 ± 339.5 × 10^6^ copies/complete final culture volume.

### 3.5. Long-Term Axenic P. jirovecii Flask Culture with DMEM-O3 Medium and Cluster Size Measurement

**Figure 10:**
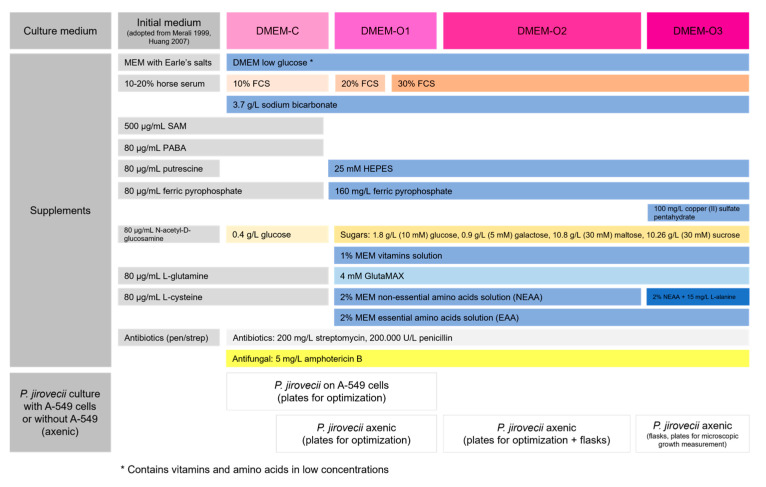
Schematic overview of the *P. jirovecii* culture optimization setup from our initial DMEM-C to DMEM-O1 and DMEM-O2 mediums and ongoing optimization with DMEM-O3 medium. FCS—fetal calf serum; SAM—S-adenosyl-L-meth3ionine sulphate; PABA—para aminobenzoic acid; pen/strep—penicillin + streptomycin; EAA—essential amino acids; NEAA—non-essential amino acids. Light grey—initial supplements and concentrations derived from the literature for (axenic) culture of rat-derived *P. carinii*; changing colors—substitution of supplements; gradients from light to dark—concentration increase in the supplement.

Our DMEM-O2 optimization experiments were completed with the addition of 15 µg/mL L-alanine, 15 µg/mL L-cystine, and 100 µg/mL copper(II)-sulphate to create our final medium, DMEM-O3 (Table 6, Figure 10).

**Figure 11:**
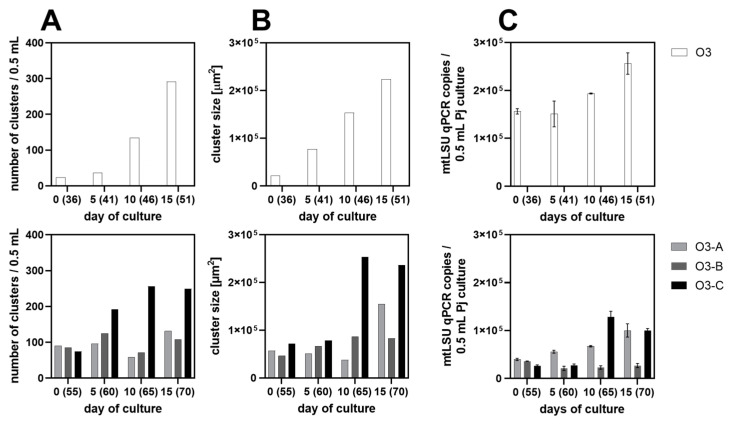
Growth of *P. jirovecii* clusters isolated on days 35 (single replicate: O3, upper line) and 55 (biological triplicates: O3-A, -B, and -C, lower line), which were cultured in chamber slides with DMEM-O3 medium for 15 days. *P. jirovecii* clusters were counted on days 0, 5, 10, and 15 (**A**), and the cluster surface was measured (**B**) from the same well on each experiment day (well of day 15) to confirm *P. jirovecii* growth microscopically (see Supplementary Materials Section S12, Figures S9 and S10). To analyze *P. jirovecii* copy numbers/well (**C**), *mtLSU* qPCR was performed in technical replicates on consecutive wells. Cluster numbers, cluster surfaces, and qPCR copy numbers increased in all four culture attempts over time. Nevertheless, qPCR showed a slower increase in copy numbers in all three replicates of experiment 2, starting at day 55 of the initial *P. jirovecii* culture.

**Figure 12:**
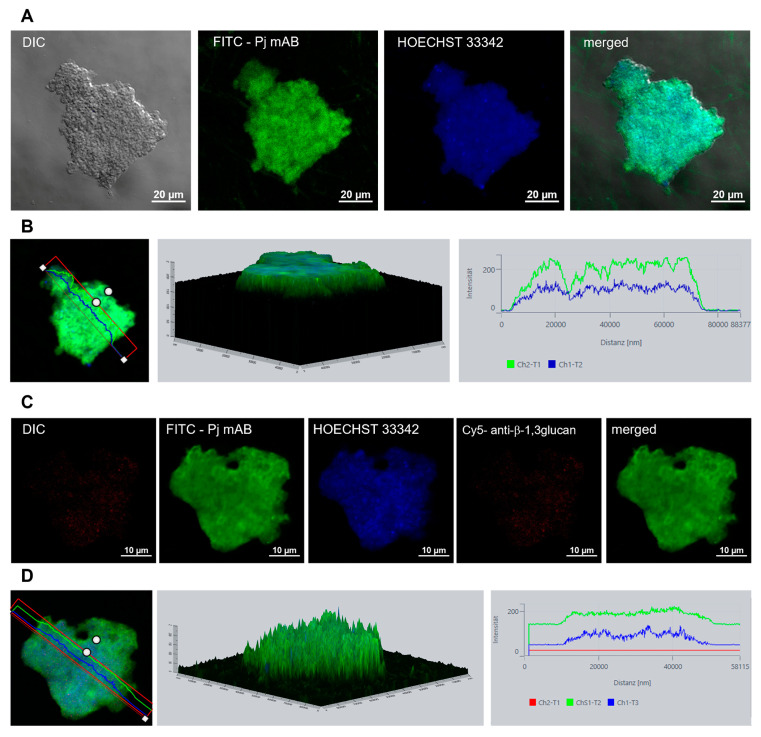
*P. jirovecii* clusters from the experiment with DMEM-O3 medium, stained with a *P. jirovecii*-specific antibody (Merifluor IVD kit, FITC-Pj mAb), HOECHST 33,342 to visualize trophozoite nuclei (**A**), and additionally with an anti-β-1,3-glucan antibody to stain the ascii (**C**). In this experiment, all visible stained clusters consisted of trophic forms but did not contain ascii, as indicated by size and morphological features, and grew relatively flat with a maximum height of 200 μm (**B**,**D**).

Two axenic *P. jirovecii* cultures in DMEM-O3 medium, V40 and V41, were used to compare *P. jirovecii* cluster numbers and cluster sizes to *P. jirovecii mtLSU* qPCR copy numbers in the medium. Growth analysis from days 36 to 41 of culture V40 showed a constant increase in *mtLSU* qPCR copy numbers (Figure 11). The number and surface area of *P. jirovecii* clusters as observed microscopically also increased during the 15 day-experiment (Figure 11 and Figure 12, Supplementary Materials Figures S9 and S10). Repeating the experiment with three replicates on days 55 to 70, the initial cluster concentration in the same medium volume was higher in all three replicates, with the result that the cluster size and *mtLSU* qPCR copy numbers/well increased more slowly but continuously. In O3-C, the number of clusters was slightly lower with 74 clusters/0.5 mL DMEM-O3 medium than in O3-A and O3-B (90 and 85 clusters), but cluster size at 71,533 mm^2^ was greater than in the other replicates (57,029 and 46,446 mm^2^) and led to increased replication of *P. jirovecii*, resulting in a 3.3-fold increase in cluster size (start of the experiment: 71,533 mm^2^, day 15: 236,691 mm^2^, Figure 11).

All *P. jirovecii* clusters had a maximum height of 200 μm and consisted of trophic forms. Isolated ascii were present in a few clusters when examined by DIC microscopy but could not be identified again after the consecutive washing steps necessary for antibody staining (Figure 12).

**Conclusions**: After an extensive literature search to evaluate promising compounds and testing them for their effects as growth enhancers for *P. jirovecii* culture in a cell-free approach, we formulated a medium including 30% FCS, amino acids, sugars, ferric pyrophosphate, copper(II) sulphate pentahydrate, and HEPES that permits the cultivation of *P. jirovecii* in an axenic approach for the first time.

## 4. Discussion

The aim of our work was to establish a culture system for the human-pathogenic fungus *P. jirovecii*. In our first culture attempt, we were able to grow *P. jirovecii* using DMEM-C medium, a new combination of supplements previously used for animal-derived *Pneumocystis* cultures on A549 feeder cells. However, the A549 cells hampered all further analysis steps such as next-generation sequencing and mass spectrometry, so we established a feeder cell-free axenic culture. Further optimization resulted in a first medium adapted to axenic *P. jirovecii* culture (DMEM-O1) and then a second medium containing new supplements for axenic culture (DMEM-O2). The final medium (DMEM-O3) successfully enhanced *P. jirovecii* growth in a long-term axenic culture. The most growth-promoting supplements for *P. jirovecii* axenic culture were glucose, sucrose, the amino acids L-cystine, L-alanine, L-glutamic acid, L-proline, ferric pyrophosphate, and copper(II) sulphate.

Due to the lack of successful culture protocols prior to our experiments, the doubling time of *P. jirovecii* was not known [15,22,62]. Nearly all *Pneumocystis* cultures published prior to our study were short-term cultures, from a five-day culture using human *P. jirovecii* to a 14-day culture using animal-derived *Pneumocystis* spp. [15,34]. Our experiments confirmed that previous *P. jirovecii* culture attempts were far too short to allow the fungus to replicate. In our cultures, the minimum doubling time was 7.3 days, resulting in a 42.6-fold increase in *P. jirovecii* after 48 days. Growth kinetics differed markedly between cultures with and without feeder cells, as also seen in *P. carinii* cultures [34]. The final, cell-free culture in DMEM-O3 medium made it possible to create *P. jirovecii* sub-cultures by splitting the medium into two bottles, followed by weekly fed-batch supplementation of fresh medium.

Only a few attempts to culture *P. jirovecii* in vitro had been published [13,15,18,29,55,63], four of which were potentially successful, but on feeder cells [13,15,29]. Of these, the results obtained by Schildgen et al. [15] had been found by the highly experienced animal-*Pneumocystis* working group headed by Kovacs to be irreproducible [16,19]. There was no published record of a successful axenic culture of *P. jirovecii*, and the systematic testing of supplements had only been carried out in a few *P. carinii* experiments [38]. In our experiments, we therefore analyzed 16 supplements in various concentrations and under various culture conditions to finally develop the DMEM-O3 medium. Animal-based *Pneumocystis* culture experiments were used as the basis for our initial media ingredients, SAM [40], ferric pyrophosphate cystine [30,64], and glutamine [30,64], which were recombined and adapted. Components such as amino acids and copper and iron compounds were taken over from mammalian cell culture media [50,65].

Resulting from optimization experiments with and without feeder cells, our final axenic flask cultures used mixtures of *P. jirovecii* strains from various BALF samples, had an inoculum of 1−2 Mio. Copies/mL medium and were cultured for 48 days. A longer culture of up to 200 days was possible, but the risk of contamination was higher.

Cultures using *P. jirovecii* from a single patient were successful but limited due to the large volume of BALF needed for inoculation of the cultures. However, single-strand cultures were not the goal of this study—our aim was simply to establish a stable culture at all. A single patient often harbors more than one strain of *P. jirovecii* at the same time [6,66,67,68], so the idea of a “single-strain” culture is specious anyway. Therefore, we used mixed *P. jirovecii* strains from 3 to 4 BALF samples, which stabilized the system and ensured growth in this first attempt, which resulted in 41 successful flask cultures. Biofilm formation, as seen in some experiments with *P. carinii* and *P. murina* [3,31], was not present in any of our cultures.

In axenic cultures, most *P. jirovecii* were attached only loosely to the surface of the culture plate, which made separate sampling of floating and sessile *P. jirovecii*, as in the first experiments, unnecessary. Coating the wells or using transwell inserts did not enhance the fungus’ attachment to surfaces.

Most *P. carinii* cultures used media with low sugar concentrations or stated that glucose did not enhance *P. carinii* growth [38]. As a result, we started our initial cultures with DMEM-C, a low-glucose medium. However, mono- and disaccharides have been described as important carbon sources in other pathogenic fungi such as *Candida* and *Aspergillus* spp. [69,70,71], and in most media for saprophytic fungi, sugars are a major component, for example, 120 g/L glucose in MY10-12 medium (DSMZ medium 982), 400 g/L sucrose in MY40 agar, and 20 g/L maltose in MY medium [72]. Thus, starting from 0.4 g/L glucose as used in the DMEM low glucose medium for *P. carinii* cultures [38], we tested ascending concentrations of glucose, the monosaccharide galactose, and the disaccharides sucrose and maltose for their growth-enhancing properties as carbon sources for *P. jirovecii*. Adding sugars as an essential carbon source, especially glucose and sucrose, markedly increased *P. jirovecii* growth in our axenic cultures. The decline in *P. jirovecii* counts on day 2 seen in all our cultures with DMEM-C medium and in *P. carinii* cultures from the literature [30,39] was probably induced by a combination of freezing damage and a lack of nutrients and was able to be reversed by adding sugars.

*Pneumocystis* spp. was already known to have impaired sulphur and nitrogen assimilation and amino acid biosynthesis. As seen in our experiments, cystine, alanine, glutamic acid, and proline increased *P. jirovecii* growth markedly.

*Pneumocystis* possesses genes for scavenging iron from the host’s haemoglobin but no genes for reductive iron uptake [6], which might explain the growth effect of ferric pyrophosphate in axenic *P. jirovecii* culture. *P. jirovecii* growth was also improved in the presence of 100 mg/L copper(II) sulphate, but higher concentrations were toxic. Similar effects have been described for *Candida* spp. and *Schizosaccharomyces pombe* [73,74], for which low copper concentrations were essential but higher concentrations inhibited growth [75].

Expensive supplements such as PABA, SAM, and putrescine were thought to be essential for *P. carinii* culture due to the presumed inability of the fungus to synthesize these substances [30,40,58,59,60,61]. However, SAM dramatically decreased organism viability in some experiments with *P. carinii* and *P. murina* [31], and as these expensive supplements had no positive effects on our cultures, we removed them from the initial DMEM-C medium. All other supplements and concentrations tested only had a weak effect and had no effect on *P. jirovecii* culture.

Before focusing on axenic culture, we grew *P. jirovecii* on A549 feeder cells in DMEM-C and DMEM-O1 medium. A549 is a well-described human lung carcinoma cell line postulated as an appropriate model for primary alveolar type II cells [76]. It contains the surfactant-related lipids and glycoproteins proposed to be essential for *Pneumocystis* nutrition [77,78] and which seem to propagate *P. carinii* and *P. murina* growth [29,79]. Attachment to feeder cells has been described in animal-*Pneumocystis* culture attempts [29,31,79,80], but was not known to affect *P. jirovecii*. In our cultures, the lower part of the *P. jirovecii* clusters was connected to the A549 layer, and the upper part floated in the medium. Large clusters tended to detach from the feeder cell layer.

When *P. jirovecii* was cultured on A549, however, the massive presence of human DNA and proteins was a major obstacle to all subsequent analysis methods. Therefore, our final goal was to establish a stable axenic culture that did not involve other cells. Nevertheless, the culture of *P. jirovecii* is an important tool for analyzing the interactions of the fungus with its host cells; therefore, this culture model might be important as well.

Due to the fact that *Pneumocystis* spp. grow in clusters, adjusting *P. jirovecii* numbers in the inoculum was difficult but an essential requirement for all experiments. An inoculum below one million *P. jirovecii* copies/mL medium and above four to five million copies/mL led to starvation of the cultures. We hypothesize that this might be due to a lack of cell contact in less dense cultures or the rapid consumption of culture nutrients followed by malnutrition in high-density cultures. LiveDead staining revealed that large clusters of *P. jirovecii* organisms seemed to be malnourished; possibly the medium supplements could not permeate the enclosed *P. jirovecii* organisms. This would explain the decrease in *P. jirovecii* at some time points during culture. After the degradation of dead *P. jirovecii*, the clusters can grow again, reaching the next growth peak. It is also possible that the ethidium homodimer-1 (eth-hd1) signal might have been induced by constantly permeable membranes due to membrane blurring and not due to the starvation of *P. jirovecii*. After supplementing the medium with nutrients such as L-alanine, as in the DMEM-O3 medium, LiveDead staining detected less eth-hd1 signal and thus fewer starving *P. jirovecii* organisms, and growth was also more constant over time.

*P. jirovecii* ascii with microdots and spores and empty and ruptured ascii with spores inside were clearly seen in our REM and TEM samples. Filopodia, rough structures visible on the surface of most ascii and spores, were also seen. These are known from rat, mouse, and rabbit *Pneumocystis* spp. and may serve to attach the individual organisms to each other and enable them to interact closely with other cells [18,81]. Interestingly, we were also able to detect *P. jirovecii* membrane blurring with TEM microscopy, as previously seen in rat *Pneumocystis* [33]. This might indicate that the *P. jirovecii* organisms were closely attached to and interacting with each other. In some TEM studies of *P. oryctolagi*, the presence of one single, large mitochondrium has been discussed [82]. We saw some fractions of mitochondria in the *P. jirovecii* organisms, mainly in the spores. Assessment of a few thin sections did not allow us to count the mitochondria present in one *P. jirovecii* cell, and the different life stages may have distinct numbers of mitochondria.

For direct microscopic examination of *P. jirovecii* growth, DIC microscopy was the most suitable method. All staining methods involved washing steps leading to organism loss or fixation, making direct observation of growing *P. jirovecii* impossible. Therefore, we decided to examine the clusters from one well using DIC microscopy and to use the other wells for parallel qPCR analysis, LiveDead staining, and antibody staining. The microscopic counting of single organisms in the clusters was hampered by the large numbers of trophic forms per cluster. Staining with LiveDead and HOECHST 33,346 showed that nearly all trophic forms within the cluster were possibly very tightly bound or nearly fused together, as seen by the distribution of the dyes within the clusters. This confirmed the observed membrane blurring seen in the REM microscopy, explaining why the *P. jirovecii* clusters could not be dissolved easily. This led to variation in inoculum and also in sampling replicates, which in turn explains the fluctuating growth curves we saw in *P. jirovecii*, which were also present in cultures involving *P. carinii* [39]. In our *P. jirovecii* cultures, up to 95% of organisms were trophic forms and were clearly distinguishable from ascii due to their size, shape, and the number of stained nuclei per cell. Ascus formation was rarely observed after a minimum of 14 culture days. Nevertheless, only a few stage-specific markers for *P. murina*, such as a monoclonal antibody against Endo-β-1,3-glucanase (Eng), p57, or MSG, were published [83,84]. However, all antibodies for these potentially stage-specific markers were either developed using *P. murina* life stages, are not commercially available, or were present on both the cystic and the trophic forms, as was the case with the highly variable MSG proteins [85,86,87]. Labeling cysts with β-1,3-glucan was not successful in our experiments, but β-1,3 glucan in cysts could be masked by surface proteins [84,88].

All cultures were quantified by *P. jirovecii*-specific *mtLSU* qPCR. Other genes, such as *MSG*, *CytB*, *DHPS*, and *DHFR* genes, and ITS 1 and 2, were examined for sensitive and specific detection of *P. jirovecii* by qPCR, but the *mtLSU rRNA* gene was the most conserved gene with high copy numbers per cell, thus permitting the most sensitive and specific detection. Increasing *P. jirovecii mtLSU* qPCR copy numbers correlated with an increase in the number and size of *P. jirovecii* clusters seen by DIC and *Pneumocystis*-specific immunofluorescence microscopy in all experiments. It would have been inappropriate to estimate *P. jirovecii* organism numbers by microscopy because the clustering of thousands of trophic forms in one single cluster and up to hundreds of clusters of varying sizes per mL of culture medium made it impossible to count the individual organisms. Therefore, we measured *P. jirovecii* cluster numbers and surface areas and compared them with the qPCR results, and we saw an increase in all three.

## 5. Conclusions

*In summary*, the aim of this work was to develop an axenic *Pneumocystis jirovecii* culture. An initial *P. jirovecii* culture was created in DMEM-C medium, both with and without feeder cells. Afterwards, an optimized culture medium, DMEM-O1, permitted the successful establishment of the first long-term cell-free culture of *P. jirovecii*. Then, the cell-free culture was scaled up for long-term flask culture using a further optimized DMEM-O2 medium, which evidenced great growth enhancement. The final optimized medium, DMEM-O3, exhibited beneficial characteristics for successful *P. jirovecii* culture. Using this culture method, we are now able to study this important and previously uncultivable fungus. 

## Figures and Tables

**Table 3:** *Optimization* of basic axenic *P. jirovecii* culture conditions in 6-well culture in DMEM-C medium. − decline/starvation of *P. jirovecii*, (+) steady state (*P. jirovecii* count stable over the whole culture duration); + slow growth, +++ fast growth with large *P. jirovecii* clusters.

Conditions and Additives	Conditions Tested	*P. jirovecii* Growth in Axenic Culture
**pH**	7.0	−
	7.5	+
	8.0	+++
	8.5	+
	9.0	−
**Temperature**	31 °C	−
	35 °C	(+)
	37 °C	+++
**Coated plates**	Gelatine	−
	Poly-L-lysin	−
**Transwell plates**		−
**Medium exchange**	every 2 days	+++
	every 6 days	+
	None	−

**Table 4:** Composition of the *P. jirovecii* DMEM-optimized medium 1 (DMEM-O1) and culture conditions for axenic culture of mixed *P. jirovecii* strains in optimization experiments.

**Culture Conditions:**	
37 °C 5% CO_2_ 1 mL/well in 6-well plates Duration: 14 days Medium exchange and sampling every 2nd day	
**Medium Ingredients:**	
DMEM low glucose	
Sodium bicarbonate	3.7 g/L
Amphotericin B	5 µg/mL
Penicillin G	200 U/mL
Streptomycin	200 µg/mL
FCS	20%
S-adenosyl-L methionine	500 µg/mL
Glutamax	20 mL/L
Glucose	3.4 mg/mL
Ferric pyrophosphate	80 µg/mL
Putrescine	8 µg/mL
2-mercaptoethanol	100 µg/mL
HEPES	12.6 µg/mL

**Table 5:** Composition of the *P. jirovecii* DMEM-optimized medium 2 (DMEM-O2) and culture conditions for axenic culture of mixed *P. jirovecii* strains.

**Culture Conditions:**	
37 °C 5% CO_2_ 1 mL/well in 6-well plates 10 mL in 25 cm^2^ flasks Duration: up to 75 days Medium fed-batch addition and sampling every 6 days	
**Medium Ingredients:**	
DMEM low glucose	
Sodium bicarbonate	3.7 g/L
Amphotericin B	5 µg/mL
Penicillin G	200 U/mL
Streptomycin	200 µg/mL
FCS	30%
Glutamax	20 mL/L
Glucose	3.4 mg/mL
Galactose	0.9 mg/mL
Maltose	10.8 mg/mL
Sucrose	1.3 mg/mL
Ferric pyrophosphate	160 µg/mL
HEPES	12.6 µg/mL
MEM essential amino acids solution (50×)	2%
MEM non-essential amino acids solution (100×)	2%

**Table 6:** Composition of the optimized *P. jirovecii* DMEM medium 3 (DMEM-O3) and culture conditions for axenic culture of mixed *P. jirovecii* strains.

**Culture Conditions:**	
37 °C 5% CO_2_ 1 mL/well in 6-well plates 10 mL in 25 cm^2^ flasks Duration: up to 75 days Medium fed-batch addition and sampling every 6 days	
**Medium Ingredients:**	
DMEM low glucose	
Sodium bicarbonate	3.7 g/L
Amphotericin B	5 µg/mL
Penicillin G	200 U/mL
Streptomycin	200 µg/mL
FCS	30%
Glutamax	20 mL/L
Glucose	3.4 mg/mL
Galactose	0.9 mg/mL
Maltose	10.8 mg/mL
Sucrose	1.3 mg/mL
Ferric pyrophosphate	160 µg/mL
HEPES	12.6 µg/mL
MEM essential amino acids solution (50×)	2%
MEM non-essential amino acids solution (100×)	2%
L-alanine	15 µg/mL
L-cystine	15 µg/mL
Copper(II) sulphate pentahydrate	100 µg/mL

## Data Availability

The data presented in this study are available on request from the corresponding author.

## Supplementary Materials

The following supporting information can be downloaded at: https://www.mdpi.com/article/10.3390/jof9090903/s1. Figure S1. *P. jirovecii MSG* gene sequences (a short excerpt of 14 DNA fragments with primer binding sites out of 295 *P. jirovecii MSG* gene sequences). Figure S2. Comparison of the sensitivity of the *mtLSU rRNA* gene qPCR with two qPCRs with primers JQ8 and ML40 as well as PCPfor and PCPrev for the detection of the *P. jirovecii MSG* gene by qPCR. Figure S3. Standards (10^5^–10^8^) plot the *bla* gene and the *P. jirovecii mtLSU* gene from the Ct value of standards. (A) R^2^ values of 0.993 and 0.984 for the bla gene and the *P. jirovecii mtLSU* gene. Theoretical PCR efficiency was 85.0% and 105.0% for the bla gene and the *P. jirovecii mtLSU* gene. (B and C) Variances between qPCR cyclers were analyzed with a dilution series of *P. jirovecii mtLSU* rRNA gene standards for absolute quantification. Due to the higher variances in qPCR results in cycler 2, we used cycler 1 for all *Pneumocystis* quantification experiments. Figure S4. Our qPCR detects and quantifies the mtLSU rRNA gene specific for *P. jirovecii*. With standards developed and used in this qPCR, an absolute quantification of *P. jirovecii mtLSU rRNA* gene copies/mL BALF showed that all HIV-positive patients suffering from PCP had *P. jirovecii* copies above one million copies/mL BALF, while HIV-negative patients with PCP had a much lower copy number, as seen in the single patient’s BALF with a copy number of 39,000 copies/mL BALF that was used for the *P. jirovecii* culture. Figure S5. *P. jirovecii* growth in cultures from the BALF of three patients in DMEM-C medium with A549 cells (left side) and axenic culture (right side). Samples were analyzed separately by qPCR for floating *P. jirovecii* (fPj) and sessile *P. jirovecii* (sPj), and the results were used to calculate the total number of *P. jirovecii*. After day six of culture, *P. jirovecii* clusters were visible in all wells. The dotted lines show the maximum growth achievable with this medium in all culture attempts, which was only surpassed by *P. jirovecii* growth in the cultures from sample 1 (A-1 and A-2). In these cultures, a growth peak can be seen after 6–10 days. Two further cultures showed a decline or steady state in both cultures with and without A549 cells, possibly due to a low *P. jirovecii* inoculum. Figure S6. *P. jirovecii* sub-culture of an isolate from sample 1 on A549 cells. Initially, the *P. jirovecii* isolate was cultured on A549 cells for 14 days with medium exchange and separate sampling of floating and sessile *P. jirovecii* organisms every 2nd day (A). On day ten, 200 µL of culture supernatant containing the scraped A549 cells and the *P. jirovecii* organisms was mixed with 6 mL of fresh DMEM-C culture medium. One mL of this *P. jirovecii*-A549 mix was added to each well of a 24-well plate and then incubated at 37 °C and 5% CO_2_ (B). Sampling and medium exchange of this sub-culture were performed on day 10 (=day 0 of the sub-culture) and then every 2nd day as in the initial culture. In axenic cultures, a sub-culture was formed by splitting the culture volume. Figure S7. Culture supplements and concentrations tested for improving the growth of *P. jirovecii* with DMEM-C medium on A549 cells. *P. jirovecii* was grown in 24-well plates for 10–14 days and quantified by qPCR. Growth was examined microscopically every 2nd day. EAA—essential amino acid mix 50×, NEAA—non-essential amino acid mix; SAM—S-adenosyl-L-methionine, PABA—para aminobenzoic acid. Figure S8. Growth of *P. jirovecii* in 24 axenic long-term cultures with a minimum of 22 and a maximum of 57 days of fed-batch flask culture. Culture conditions except patient samples for inoculum, duration of culture, and final culture volume were not altered. All cultures were grown in DMEM-O2 medium (starting volume 10 mL/flask) and cultured at 37 °C and 5% CO_2_. The medium was not exchanged completely, but samples for qPCR analysis were taken, and increasing volumes of new medium were added on top of the old medium to dilute the growing *P. jirovecii* to appropriate densities in the flask and add fresh supplements (fed-batch culture). As seen after the first five cultures, a start inoculum of 1 to 2 million copies/mL culture medium volume was desirable, as both well plate cultures and flask cultures with higher or lower inoculum showed decreasing *P. jirovecii* copy numbers/mL medium. Nevertheless, due to *P. jirovecii* clumping, some cultures had higher concentrations of *P. jirovecii* inoculum. A medium increase of up to 60% during culture and a duration of 48 days led to the largest increase in *P. jirovecii*, with an optimum doubling time of 6.9 to 8.8 days and a max. 42.6-fold increase. Figure S9a–e. Growth of *P. jirovecii* in DMEM-O3 medium, day 36 after *P. jirovecii* culture started, day 0 of the growth experiment. The chamber slide was incubated at 37 °C and 5% CO_2_, as were all other *P. jirovecii* cultures. Medium fed-batch was not performed in this experiment to avoid moving the *P. jirovecii* clusters. Each of the four upper wells of a chamber slide was filled with 0.5 mL of DMEM-O3 containing *P. jirovecii* organisms harvested from the actual flask culture V40. Well 4 was thoroughly examined with DIC 400× magnification (Cellobserver microscope, Zeiss) on days 0, 5, 10, and 15. Clusters were photographed, counted, and the surface measured. Figure S9a (actual picture) shows the total number of *P. jirovecii* clusters on day 0 of the experiment (*n* = 23); Figure S9b shows the *P. jirovecii* clusters on day 5 (*n* = 36); Figure S9c,d show the *P. jirovecii* clusters on day 10 (*n* = 134), Figure S9e–h show the *P. jirovecii* clusters on day 15 (*n* = 291). Figure S10. A single *P. jirovecii* cluster extracted from a culture in DMEM-O2 medium on day 14 contains thousands of individual organisms. To distinguish between different life stages (spore, haploid trophic form, diploid trophic form, and ascus), the organism diameters on the surface of one single cluster were measured and differentiated by size into spore, haploid trophic form (*n*), and diploid trophic form (2n). In this cluster, spores and haploid trophic forms represented the majority of the organisms; diploid trophic forms that developed into ascii were rare. Table S1. *Pneumocystis* species in humans and animals. Grey—species name suggestions outside taxonomy. Table S2. Most common primers and probes for the detection of the *Pneumocystis MSG* gene. Table S3. (Table 1 continued): Significant animal-derived *Pneumocystis* spp. culture attempts. Table S4. *Optimization* of basic *P. jirovecii* culture conditions in 6-well culture with A549 cells and axenic culture without feeder cells in DMEM-C medium−decline/starvation of *P. jirovecii*; (+) steady state (*P. jirovecii* count stable over the whole culture duration); + slow growth; +++ fast growth with large *P. jirovecii* clusters. References [3,6,7,9,10,13,15,17,18,23,26,29,30,31,34,36,39,41,43,45,46,47,52,54,55,56,59,63,80,85,89,90,91,92,93,94,95,96,97,98,99,100,101,102,103,104,105,106,107,108,109,110,111,112,113,114,115,116,117,118,119,120,121,122,123,124,125,126,127,128,129,130,131,132,133,134,135,136,137,138,139,140,141,142,143,144,145,146,147,148,149,150,151,152,153,154,155,156,157,158,159,160,161,162,163,164,165,166,167,168,169,170,171,172,173,174,175,176,177,178,179,180] are cited in the supplementary materials.
