# Supplementary material for: Axenic Long-Term Cultivation of Pneumocystis jirovecii
Source: J Fungi (Basel). 2023 Sep 1;9(9):903. doi: 10.3390/jof9090903 (PMC10533121; doi:10.3390/jof9090903)
Supplement: Supplementary file 1 [file jof-09-00903-s001.zip › jof-2540415-supplementary.pdf]

**Supplementary Material**

***S1.***

***Pneumocystis* spp. prevalence in wild, domestic and laboratory animals – Careful differentiation of species is necessary when reading *Pneumocystis* culture papers.**

*Pneumocystis* spp. was initially detected by Carlos Chagas in the lungs of guinea pigs *Cavia porcellus* in 1909 [89]. Nevertheless, after that, no other *Pneumocystis* infections were described in these small rodents and only few researchers used guinea pigs for *Pneumocystis* infection experiments.

*Pneumocystis* organisms, or perhaps their DNA, were detected in a huge variety of mammal species, and some birds [89–91]. Due to molecular studies – even if only small DNA fragments of a single gene [92,93] or a so-called "mini-typing" detecting and analysing a set of genes, it is clear, that mostly each animal harbours its own *Pneumocystis* species, which is clearly genetically different from *Pneumocystis* spp. of other host animals [34,46]. As far as known, few cross-infections experiments were done, as described in guinea pigs successfully infected with rat-derived *P. carinii*, instead golden hamsters seem to be non-susceptible to *P. carinii* of rat strain [94]. Cross-infection experiments with cats and mouse-derived *Pneumocystis* spp. as well as mice and rats as recipient failed [62,95–97]. Nevertheless, the data about cross-infection experiments or natural cross-infections are sparse.

Some authors mentioned that animal-derived *Pneumocystis* species can be detected in human samples as contaminants from air when performing NGS analysis [45]. Therefore, we designed a *mitochondrial transcribed large subunit rRNA gene (mtLSU)* PCR [46], which should be theoretically able to detect all known *Pneumocystis* species to verify that our samples for culture does only contain human *P. jirovecii* strains. **Table S1** shows the confusing number of *Pneumocystis* subspecies, unofficial names, and synonyms, which are present in the literature, mainly the NCBI Taxonomy database, due to changing the name of the human *Pneumocystis* species from "*P. carinii*", which was the identical designation for all *Pneumocystis* spp. until 2002, or sometimes "*P. carinii* f. sp. *hominis*" to "*P. jirovecii*". This makes the differentiation in new papers much easier, but is not helpful for the earlier culture papers, where the human species is still named "*P. carinii*" and the source of organisms is often not mentioned. Careful reading is necessary to identify the experiments, which truly used the human *P. jirovecii* species. The table does not claim comprehensiveness, but hopefully honours all researchers who tried to find new insights in this odd organism.

**Table S1.** *Pneumocystis* species in humans and animals. Grey – species name suggestions outside taxonomy.

| *Pneumocystis* subspecies | Host | *Pneumocystis* synonyms in publications | First / important articles | Used for *in vitro* culture | Used for *in vivo* culture |
|---|---|---|---|---|---|
| *Pneumocystis* sp. | Guinea pigs *Cavia porcellus* (First detection of the organism) | *P. carinii* | [89] | - | [98] |
| *Pneumocystis jirovecii* | Humans | Until 2002 named as *P. carinii* or *P. carinii* f. sp. *hominis* | [99], former known as "interstitial pneumonia"[7,9,45] | [13, 15, 17, 18, 30, 63, 100] | - |
| *Pneumocystis carinii*<br>*P. carinii* f. sp. *rattus-secundi*<br>*P. carinii* f. sp. *rattus-tertii*<br>*P. carinii* f. sp. *rattus-quarti* | Wild rats *Rattus norvegicus*,<br>laboratory rats | *P. carinii*,<br>*P. carinii* f. sp. *carinii* | [101] | [80,102] | [52,103–109] |
| *Pneumocystis wakefieldiae* | Wild rats *Rattus norvegicus* | | [10,96,110] | - | - |
| *Pneumocystis murina* | Laboratory mice *Mus musculus*<br>*Wild mice* | *P. carinii*,<br>*P. carinii* f. sp. *muris* | [111]<br>[112] | [36] | [36,103,111,113] |
| *Pneumocystis oryctolagi* | Rabbits *Oryctolagus cuniculus* | *P. carinii* | [10,23] | [26] | [103,114–116] |
| *Pneumocystis sp.* | Hares *Lepus europaeus* | | [117] | - | - |
| *Pneumocystis* sp. *Gerbil* | Mongolian gerbils *Meriones unguiculatus* | | [105] | - | [104] |
| *Pneumocystis canis* | Dogs | | [10,118,119] | - | [[120] |
| *P. carinii* f. sp. *Mustelae* | Ferrets | | [121] | - | - |
| *Pneumocystis sp.*<br>*P. carinii* f. sp. *macacae*<br>*P. carinii* f. sp. *callimico*<br>*P. carinii* f. sp. *callithrix*<br>*P. carinii* f. sp. *midas*<br>*P. carinii* f. sp. *fuscicolis*<br>*P. carinii* f. sp. *sciureus*<br>*P. macacae* | Monkeys<br>Rhesus macaca *Macaca mulatta*<br>Goeldi's monkey *Callimico goeldii*<br>Geoffroy's marmoset *Callithrix jacchus*<br>Red-handed tamarin *Saguinas midas*<br>Brown-handed tamarin *Leontocebus fuscicolli*<br>Squirrel monkey *Saimiri sciureus*<br>Black lemur *Eulemur (Maki) macaco* | | [92,122]<br>[92, 93]<br>[93]<br>[93]<br>[93]<br>[93]<br>[93]<br>[10] | - | - |
| *P. carinii* f. sp. *Suis* | Pigs | | [123] | - | [124] |
| *Pneumocystis* sp. | Horses | | [125] | - | - |
| *Pneumocystis* sp. | Bats, various species<br>Bat *Glossophaga soricina* | | [126-128]<br>[92] | - | - |
| *Pneumocystis* sp. | Greater grison *Galictis vittata* | | [92] | - | - |
| *Pneumocystis sp.* | Squirrel Sciureus estuans | | [92] | - | - |
| *Pneumocystis sp.*<br>*Pneumocystis* sp. 1 PD-2016 | Shrews<br>Valais shrews *Sorex antinorii*<br>*Sorex araneus*<br>*Sorex caecutiens*<br>*Notiosorex crawfordi* | | [129-137] | - | - |
| *Pneumocystis sp.* | Voles: Field vole *Microtus agrestis* | | [132, 138, 139] | - | [139] |
| *Pneumocystis* sp. | Domestic goats | | [140] | - | - |
| *Pneumocystis* sp. | Cats | | [119] | - | [95, 120] |
| *Pneumocystis* sp. | Birds (First occurrence outside mammals)<br>Chickens | | [90, 91, 141]<br>[91] | -<br>- | -<br>- |
| Unclassified *Pneumocystis*<br>*Pneumocystis* sp. *´exulans´*<br>*Pneumocystis* sp. *´fulvescens´*<br>*Pneumocystis* sp. *´ludovicianus´*<br>*Pneumocystis* sp. *´muelleri´*<br>*Pneumocystis* sp. *´tanezumi´* | <br>Polynesian rat *Rattus exulans*<br>Chestnut white-bellied rat *Niviventer fulvescens*<br>Black-tailed prairie dog *Cynomys ludovicianus*<br>Müller's giant Sunda rat *Sundamys muelleri*<br>Tanezumi rat *Rattus tanezumi* | | <br>[85]<br>[85]<br>Ma et al. Nucleotide submission<br>[85]<br>[85] | <br>-<br>-<br>-<br>-<br>- | <br>-<br>-<br>-<br>-<br>- |

## *S2.*
## *Comparison of P. jirovecii specific genes to select the optimal qPCR for quantification of the pathogen in patient samples and cultures.*

For identification of the optimal DNA fragment for specific and sensitive detection of *P. jirovecii* by qPCR, we reviewed the literature and aligned all published DNA sequences, and corresponding primer pairs and probes for *mitochondrial transcribed large subunit rRNA* (*mtLSU* rRNA) gene, *dihydropteroate synthase (DHPS)* gene, *dihydrofolate reductase (DHFR)* gene, *cytochrome B (CytB)* gene, *major surface glycoprotein (MSG)* gene, and the internal transcribed spacers (ITS) 1 and 2 of *P. jirovecii* and all animal-derived *Pneumocystis* species. ITS are highly variable, therefore primer design to detect all *P. jirovecii* strains is difficult and the sensitivity is low. But we, and other authors, use this sequence for strain typing [142-144]. *P. jirovecii* is known to have single nucleotide position changes in the *DHPS*, *DHFR*, and *cytB* genes leading to amino acid changes and resistance of *P. jirovecii* to sulfonamides, trimethoprim, and atovaquone. The *MSG* multicopy gene family has an estimated 30 to 100 copies per genome per organism and are extremely variable depending on the *P. jirovecii* isolate [47, 85, 145-147]. We use them regularly to detect *P. jirovecii* resistance mutations in our

laboratory. For *P. jirovecii* detection, the *MSG* gene was mentioned in 35 publications (references see **Table S2**). Thoroughly reviewing the literature and aligning 295 published *P. jirovecii MSG* gene sequences (with 83.4% of the sequences were 300 to 1,000 bp) and known primer pairs with CLC main software V22.0.2, it has been found that most primer pairs had different names but identical sequences in the papers and did not bind in all *P. jirovecii MSG* sequences (**Table S2, Figure S1**). We decided then to use the primer pair JQ8 + ML40 and MINC primers PCPfor and PCPrev for our analyses because they had the highest specificity to *P. jirovecii* sequences and can be used for qPCR.

**Table S2.** Most common primers and probes for the detection of the *Pneumocystis MSG* gene.

| No. | Primer or probe name | Sequence | Sequence (nearly or completely) identical to no. | References |
|---|---|---|---|---|
| 1 | Primer for | 5′-GAATGCAAATCYTTACAGACAACAG-3′ | 9 | [148][1] |
| 2 | Primer rev | 5′-AAATCATGAACGAAATAACCATTGC-3′ | 7, 10, 22, 30, 32 | |
| 3 | Probe 1 | 5′-CAAAAATAACAYTSACATCAACRAGGCG-FITC-3′ | 6, 11, 13 | |
| 4 | Probe 2 | 5′-Red 640-TGCAAACCAACCAAGTGTACGACAGG-3′ | 8, 12, 14 | |
| 5 | Primer JQ14 | 5′-GCATGCAAGCTGACATTCCGCGCAAAAATAAGCACT-3′ | 17 | [144, |
| 6 | Primer MINC PCPfor | 5′- CAAAAATAACAYTSACATCAACRAGG-3′ | 3 | 149,150] |
| 7 | Primer MINC PCPrev | 5′- AAATCATGAACGAAATAACCATTGC-3′ | 2, 10, 22, 30, 32 | |
| 8 | Probe MINC PCPprobe | 5′- FAM-TGCAAACCAACCAAGTGTACGACAGG-TAMRA-3′ | 4, 12, 14 | |
| 9 | Primer RUNMC JKK114_15 | 5′-GAATGCAAATCYTTACAGACAACA-3′ | 1 | [149, |
| 10 | Primer RUNMC JKK17 | 5′-AAATCATGAACGAAATAACCATTGC-3′ | 2, 7, 22, 30, 32 | 151,152] |
| 11 | Probe RUNMC PCMSGFRET1U | 5′-CAAAAATAACAYTSACATCAACRAGGC-3′ | 3, 6, 13 | |
| 12 | Probe RUNMC PCMSGFRET1D | 5′-TGCAAACCAACCAAGTGTACGACAGG-3′ | 4 | |
| 13 | Primer PCMSGFRET1U | 5′- CAAAAATAACAYTSACATCAACRAGGCG-3′ | 3, 6, 11 | [152-157] |
| 14 | Primer PCMSGFRET1D | 5′- TGCAAACCAACCAAGTGTACGACAGG-3′ | 4, 8, 12 | |
| 15 | Probe PCMIM1U | 5′- GATATCGTCCATTCCGACAGCATC-fluorescein-3′ | | |
| 16 | Probe PCMIM1D | 5′- Red640-CCAGTCACTATGGCGTGCTGCTAG-3′ | | |
| 17 | Primer JQ14 | 5′-GCATGCAAGCTGACATTCCGCGCAAAAATAAGCACT-3′ | 5 | [158-160] |
| 18 | Primer JQ8 | 5′-CTGTGGATTGAGCTATTTCTTGTATCTATGCGCT-3′ | | |
| 19 | Primer ML664 | 5′-CGAGGCTCCCCCAAATG-3′ | | |
| 20 | Primer ML40 | 5′-TTCAGCGCAGGTTGGTTG-3′ | | |
| 21 | Primer MSG-fw | 5′-GAATGCAAATCCTTACAGACAACAG-3′ | | [161] |
| 22 | Primer MSG-rv | 5′-AAATCATGAACGAAATAACCATTGC-3′ | 2, 7, 10, 30, 32 | |
| 23 | Probe MSG-probe | 5′-FAM-AGACATCGACACACACAAGCACGTCT-BHQ1-3′ | | |
| 24 | Primer fwd | 5′-CATCCAGGATCCGATTTCGA-3′ | | [162] |
| 25 | Primer rev | 5′-CAGCCCTATGTCCTCTGCTAGTG-3′ | | |
| 26 | Probe | 5′-FAM-CCAACTAAGCCAGAGCC-MGBNFQ-3′ | | |
| 28 | Primer JKK14 | 5′-AATGCAAATCCTTACAGACAACAG-3′ | | [163-167, |
| 29 | Primer JKK15 | 5′-GAATGCAAATCTTTACAGACAACAG-3′ | | 180] |
| 30 | Primer JKK17 | 5′-AAATCATGAACGAAATAACCATTGC-3′ | 2, 7, 10, 22, 32 | |
| 31 | Primer JK151 | 5‘-TTTCATATGGCGCGGGCGGTCAAGCGGCAG-3′ | | [168, 169] |
| 32 | Primer JK152 | 5′-CTAAATCATGAACGAAATAACCATTGCTAC-3′ | 2, 7, 10, 22, 30 | |
| 33 | Primer JK451 | 5′-GAATTCGATCTGAAGCCTCTGGAG-3′ | | |
| 34 | Primer JK452 | 5′-TTCTAGAAACCCACTCATCTTCAA-3′ | | |

In a comparative study analysing five in-house and five commercial qPCRs for *mitochondrial transcribed small subunit* (*mtSSU*) rRNA gene, *mtLSU*, *MSG*, and *β-tubulin* gene, *mtLSU* qPCR had a higher sensitivity than MSG qPCR [148]. As seen in our studies, both *MSG* qPCRs' sensitivity were nearly comparable to the mt*LSU* qPCR, but regarding the mismatches of primer binding sites and therefore possibly no detection of some *P. jirovecii* strains, we decided to use the very stable, sensitive, and specific *mtLSU* rRNA gene qPCR for all further analyses.

[1] Supplements, assay ID 11

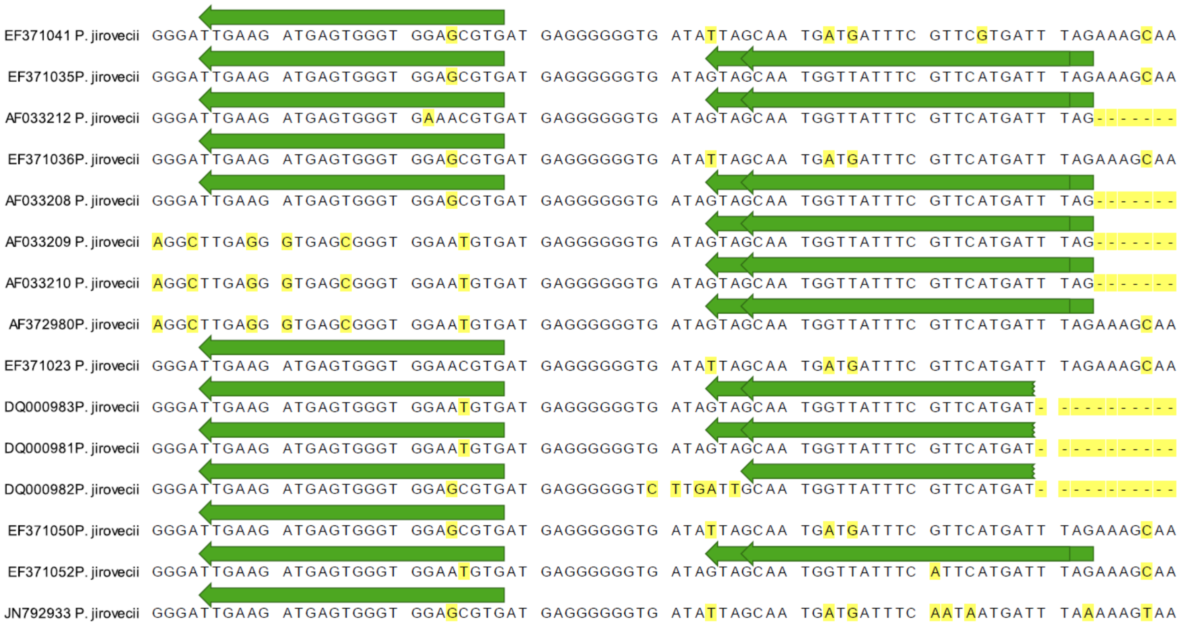


**Figure S1**. *P. jirovecii MSG* gene sequences (short excerpt of 14 DNA fragments with primer binding sites out of 295 *P. jirovecii MSG* gene sequences).

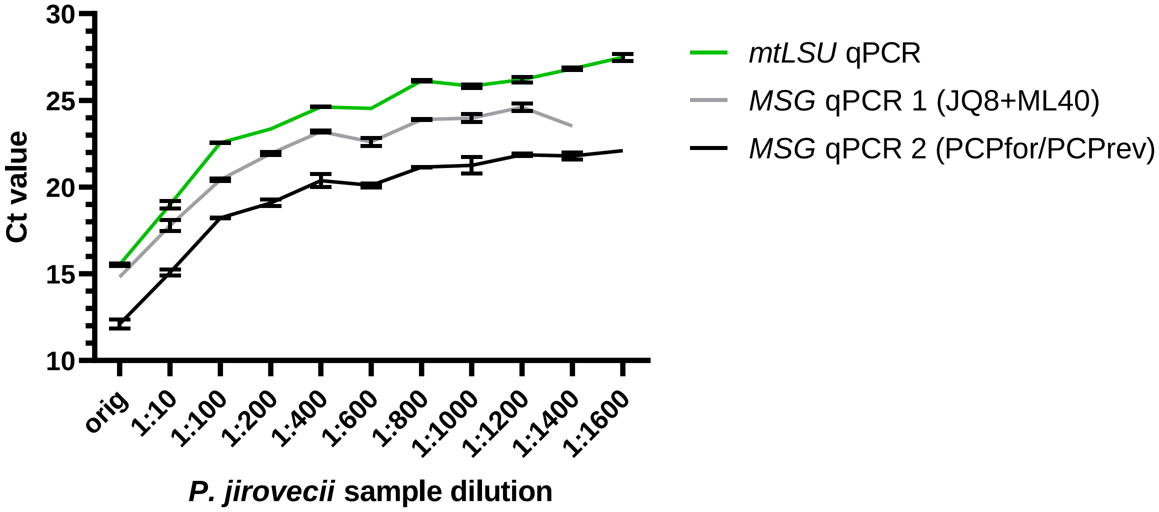


**Figure S2**. Comparison of the sensitivity of the *mtLSU rRNA* gene qPCR with two qPCRs with primers JQ8 and ML40 and well as PCPfor and PCPrev for the detection of the *P. jirovecii MSG* gene by qPCR.

***S3.***

***Production of P. jirovecii standards for absolute quantification of P. jirovecii DNA content in BALF samples by qPCR was successful and allowed direct comparison of different cultures.***

The standards for the *P. jirovecii* (Pj) *mitochondrial transcribed large subunit* (*mtLSU*) rRNA gene qPCR were produced by cloning of a *Pj*-specific *mtLSU* gene PCR product with adenine (A)-overhang into the pGEMT® vector system II plasmids DNA and afterwards transformed in *E. coli* JM109 competent cells (Promega, Mannheim, Germany) as follows:

Primers were designed with CLC main program and unique species detection specific for *P. jirovecii mtLSU* gene after "blastn" (http://www.ncbi.nlm.nih.gov/BLAST/). Primers with

optimal melting temperature (Tm) for fast qPCR protocols, low primer dimer and hairpin structure formation, as analysed with CLC main and oligo analyser version 3.1 program were chosen for further qPCR analysis. Some primers are patent pending; therefore, the sequences are not published here.

For the production of *P. jirovecii mtLSU* DNA fragments with an A-overhang at the 3′ ends, a known *P. jirovecii* -positive DNA sample was amplified with the primers for *mtLSU* rRNA gene single-round PCR, with 10 µL of the *P. jirovecii* DNA in a reaction volume of 55 µL, containing 10 mM Tris-HCl, 50 mM KCl, 2 mM $MgCl_2$, 0.2 mM of each dNTP, 1 mM of each primer (LSU-for and LSU-rev) and 2.5 U Taq polymerase. The *P. jirovecii mtLSU* PCR was performed with following conditions: Initial denaturation at 95°C for 9 min, 40 cycles amplification: denaturation at 94°C for 30 sec, annealing at 55°C for 30 sec and extension at 72°C for 30 sec and a terminal extension at 72°C for 5 min. The amplificates of 350 bp length were visualized with UV light after 1% agarose gel electrophoresis and subsequently excised and purified with the QIAquick gel extraction kit (Qiagen, Hilden, Germany). An aliquot of the purified DNA was diluted to 5 ng/µL and sequenced (Eurofins, Hilden, Germany). Sequence analysis was performed with CLC main software version 6.9 (Qiagen, Hilden, Germany) to confirm the *P. jirovecii mtLSU* gene fragment sequence.

The purified DNA fragment with A-overhang was then used for ligation with the pGEM-T vector (Promega, Germany) as well as sufficient transformation controls according to manufacturer's instructions.

Ligation success was verified by amplifying the insert by PCR with primers, which bind to a specific DNA sequence inside the vector and circumvent the *P. jirovecii*-specific inserts. For this, the plasmid isolates were analysed by pUCM13 single-round PCR with 10 µL of the plasmid DNA in a reaction volume of 55 µL, containing 10 mM Tris-HCl, 50 mM KCl, 0.125 mM $MgCl_2$, 0.5 mM of each dNTP, 0.1 mM of each primer (pUCM13-for 5′- GTT TTC CCA GTC ACG AC-3′ and pUCM13-rev 5′- GTC ATA GCT GTT TCC TG-3′) and 1.25 U Taq polymerase. The pUCM13 PCR was performed as follows: Initial denaturation at 95°C for 9 min, 45 cycles of amplification: denaturation at 94°C for 30 sec, annealing at 53°C for 30 sec and extension at 72°C for 30 sec and a terminal extension at 72°C for 5 min. When the *mtLSU* DNA fragment was inserted successful into the vector, the PCR will produce a 583 bp PCR product. Electrophoresis DNA bands were excised, purified with the QIAquick gel extraction kit, DNA content was measured with the NanoDrop and diluted to 5 ng/µL. The identity of the cloned insert was confirmed by direct sequencing and sequence analysis with CLC main software.

Following sufficient ligation, the ligated plasmids were transformed in JM-109 competent *E. coli* (Promega, Germany) and transferred to 950 µL SOC medium. After incubation at 37°C for 1.5 h, 100 µL of the transformed *E. coli* were plated onto LB/ampicillin/IPTG/X-Gal plates and incubated at 37°C for 24 h. White colonies were picked, the plasmid DNA was extracted with the QIAprep spin miniprep kit (Qiagen, Germany) and screened by *mtLSU* PCR, as described above, in order to identify clones that contain the *P. jirovecii mtLSU* PCR product. *E. coli* clones with successfully Pj DNA fragment insertion were then frozen in cryobank vials (Mast Diagnostica, Germany) at -80°C for further production of *P. jirovecii* PCR positive controls.

For the *P. jirovecii* insert:plasmid ratio and to quantification of the *P. jirovecii* DNA in copies/mL, the plasmid DNA needs to be quantified by concurrent amplification of the beta-lactamase (*bla*) gene and the *LSU* gene fragment by qPCR of plasmid isolates with adjusted

DNA content. For that, the plasmid DNA content was measured with the Qubit® dsDNA high sensitivity kit (Thermo Fisher, Germany) and adjusted to 2 ng/µL.

For analysis of the insert:plasmid ratio, first, the *beta-lactamase* gene (bla) was detected by SYBRgreen qPCR with primers bla-for 5′- CTA CGA TAC GGG AGG GCT TA-3′ and bla-rev 5′- ATA AAT CTG GAG CCG GTG AG-3′. For this PCR, all PCR reagents and DNA samples were pipetted on ice with a CAS-1200 qPCR pipetting robot (Corbett Robotics, now Qiagen, Hilden, Germany) for improved accuracy. For each PCR run, 0.2 mM of each primer, 10 µL SensiMix™ SYBR® No-ROX kit (Bioline meridian bioscience, UK), 4.2 µL DNAse free water and 5 µL of *P. jirovecii* plasmid DNA extracts for a final volume of 20 µL. All samples were run in triplicates. The amplification was carried out in two Rotor-Gene Q dual-plex and Rotor-Gene 6000 six-plex cyclers (Corbett Robotics, now Qiagen, Hilden, Germany) with following PCR conditions: Initial denaturation at 95°C for 10 min and followed by 40 amplification cycles with denaturation at 95°C for 15 sec, annealing at 60°C for 20 sec and extension at 72°C for 20 sec with acquisition on green channel. Subsequently a melting curve with ramp 72-95°C and 1°C/step heating rate and gain optimisation was done. Results were considered positive when a significant fluorescent signal above the baseline (threshold, Ct) was detected.

The second qPCR was performed for detecting the *P. jirovecii LSU* gene fragment by specific qPCR with primers *P. jirovecii* LSU-for4 and PjLSU-rev2 and Taqman probe Pj*LSU*-probe (primer and probe sequences are patent-pending), which were newly designed and optimised for use in combination with the primers and probe for the internal control (IC) of the QuantiFast Pathogen + IC Kit (Qiagen, Hilden Germany). For each PCR run, 0.01 mM of each primer, 5 pM of PjLSU probe, 2,5 µL IC DNA, 2,5 µL IC primers + probe mix, 5 µL pathogen mastermix, 8.75 µL DNAse free water and 5 µL of *P. jirovecii* plasmid DNA extracts for a final volume of 25 µL. All samples were run in triplicates. PCR reagents and DNA samples were pipetted on ice with a CAS-1200 qPCR pipetting robot. The amplification was carried out in the Rotor-Gene Q PCR cycler with following conditions (fast protocol): The initial denaturation was done for 5 min at 95°C and the amplification was performed for 35 cycles of denaturation at 95°C for 15 s, combined annealing and extension at 60°C for 75 s with acquisition on green and yellow channel. Results were considered positive when a significant fluorescent signal above the baseline was detected. This qPCR produces a DNA fragment of 188 bp, which is detected via the FAM channel. The internal control DNA (IC) was detected via the HEX channel.

This qPCR protocol was also used for absolute quantification of *P. jirovecii* in all culture attempts using two to three of the following standards.

The DNA contents of the *bla* gene and *P. jirovecii LSU* gene PCRs were compared by calculation of the copy numbers / µL and gene and the ΔCt and ΔΔCt values as follows:

$$DNA\ copies = \frac{6{,}02 * 10^{23}\ \left(\frac{copies}{mol}\right) * DNA\ amount\ (g)}{DNA\ length\ (dp) x\ 660\ \left(\frac{\frac{g}{mol}}{dp}\right)}$$

$$\Delta Ct = Ct\ target - Ct\ reference = Ct\ \textit{P. jirovecii LSU}\ gene\ PCR - Ct\ \textit{bla}\ gene\ PCR$$

$$\Delta\Delta Ct = \Delta Ct\ sample - \Delta Ct\ calibrator$$

Then, the *P. jirovecii* DNA content was adjusted to 100 million copies/mL and dilution series of were produced. Ct values for standards values ranging from 10 to $10^9$ copy numbers of *bla*

gene and *P. jirovecii mtLSU rRNA* gene fell along a straight semi-log trendline with a R2 value of 0.9902 and 0.9964, respectively (**Figure S3A**).

Comparison of copy numbers of the *bla* gene, which is present in a single copy per plasmid, and the *mtLSU* rRNA gene inserted in these plasmids, showed a ratio of nearly 1:1, indicating that only one *mtLSU* rRNA gene copy is present per plasmid and therefore, a direct absolute quantification was possible. Linearity of the qPCR standards was present when diluting the standards from $10^8$ to $10^5$ copies/mL. The diluted four standards for 100 million, 10 million, 1 million, and 100,000 copies/mL were then used in any LSU qPCR run for calculating the standard curve. The qPCRs for standardisation were performed on all qPCR cyclers in minimum of three runs with triplicates for each standard (**Figure S3 B and C**).

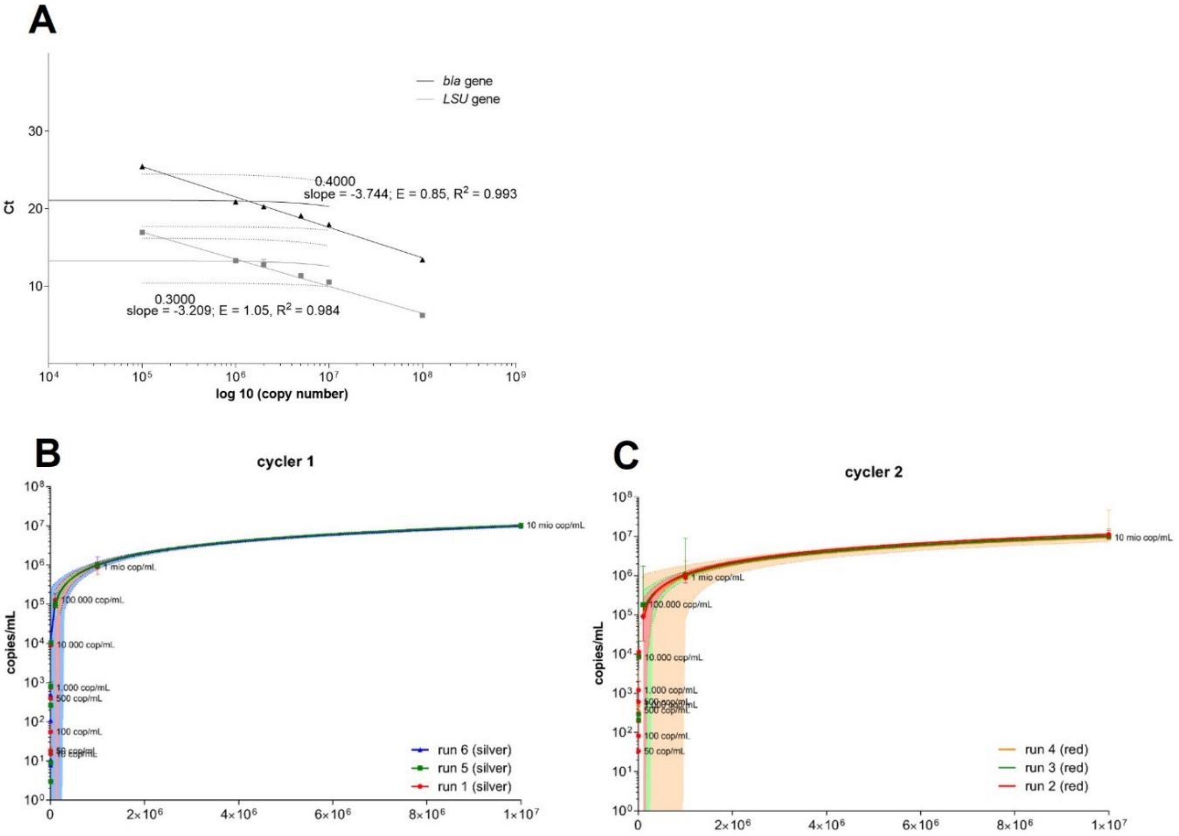


**Figure S3.** Standards ($10^5$–$10^8$) plot for *bla* gene and *P. jirovecii mtLSU* gene from Ct values of standards. A) $R^2$ value of 0.993 and 0.984 for bla gene and *P. jirovecii mtLSU* gene. Theoretical PCR efficiency was 85.0% and 105.0% for bla gene and *P. jirovecii mtLSU* gene. B and C) Variances between qPCR cyclers used. Analysis with dilution series of *P. jirovecii mtLSU* rRNA gene standards for absolute quantification. Due to the higher variances in qPCR results in cycler 2, we used cycler 1 for all *Pneumocystis* quantification experiments.

Specificity of the *P. jirovecii mtLSU* gene qPCR was proven by analysis of DNA-Isolates from the most important lung pathogens (*S. aureus*, *S. pneumonia*, non-typable *H. influenzae*, *K. pneumoniae*, *P. aeruginosa*, *C. cladosporioides*, *M. catarrhalis*, *C. neoformans*, *C. albicans*, and *A. fumigatus*) and organisms present in the normal environment (*P. brevicompactum*, *S. cerevisiae*), which gave no positive qPCR results. Additional, specificity of the desired *P. jirovecii mtLSU* gene primers was also proven with SYBRgreen qPCR with the PCR program 95°C for 10 min followed by 35 cycles of 95°C for 15 sec, 60°C for 20 sec with detection on green channel and 72°C for 20 sec). Subsequent melting curve analysis (ramp 72 to 95°C, 1°C per step, 90 sec pre-melt conditioning and 5 sec after each step with detection on green channel with 95% gain optimisation) and additional sequencing of the PCR products after gel extraction

was done to confirm the specificity of the PCRs. *mtLSU* primer-dimers were not detectable. The *P. jirovecii* specific amplificates had a melting temperature of 76.5°C. Therefore, the primers could also be used for SYBRgreen qPCR detection.

Our *mtLSU* qPCR assay could systematically detect the dilution containing one copy of plasmid per µL, leading to a detection sensitivity of at least five copies per PCR. Quantification was linear over an order of magnitude of 8, and the standard curve was generated with a good coefficient of determination ($R2$ = 0.9918, **Figure S3**). For the *P. jirovecii* DNA positive control of 10 million copies, the mean Ct on 40 runs was 15.21±0.83 (CV = 5.51%). Three different standards were used for all qPCR analyses. For quality control of the standards used for quantification of the cultured *P. jirovecii*, a maximal variation of the 10 Mio copies standard of 15±1 Ct was allowed. Otherwise, the standards were replaced. Specificity of the qPCR could be confirmed by the analysis of DNA-isolates from important lung pathogens (species: see "Confirmation of primer specificity"), none of them was qPCR-positive.

As seen in cultures of animal-derived *Pneumocystis* and our *P. jirovecii* cultures, traditional staining methods, were not useful, because (i) *P. carinii* grows in clumps, what makes enumerating cells by counting nuclei nearly impossible [30], (ii) culture supplements, as FCS, inhibit appropriate staining [170], and (iii) a mixture of sporozoites and cysts was present, and (iv) nearly 90% of all cultured animal-*Pneumocystis* are fragile sporozoites [30, 59]. For qPCR quantification, information about gene copies per haploid or diploid sporozoite or cyst were lacking. Due to these points, a direct quantification in "organisms/mL" was not possible. Therefore, we used the unit "copies/mL" for the quantification of the *P. jirovecii mtLSU* gene by qPCR, which was the primary method in all BALF samples and cultures. In our cultures, the DNA of impaired sporozoites was degraded immediately by DNAses, which were present in the FCS [171], therefore only DNA of living *P. jirovecii* was quantified. *P. jirovecii* grows in those large clumps, so it is difficult to use the traditional method of enumerating cells by counting nuclei in Giemsa-stained smears. Additionally, Giemsa and DiffQuick stain results are altered – the staining is faded nearly completely - possibly due to the high content of proteins from the FCS used in the medium and exact counting from staining methods was not possible. Therefore, absolute quantification of *P. jirovecii* specific DNA by qPCR was used for our studies.

### ***S4.***
### ***Quantification of P. jirovecii in BALF samples by mtLSU rRNA gene qPCR for adjustment of P. jirovecii culture inocula.***

As seen in our culture experiments, the *P. jirovecii* start inoculum is crucial for the success of the cultures. Therefore, we analysed all BALF samples used in this study with the *mtLSU rRNA gene* qPCR (**Figure S4**) and diluted the patient`s samples to the desired *P. jirovecii* amount.

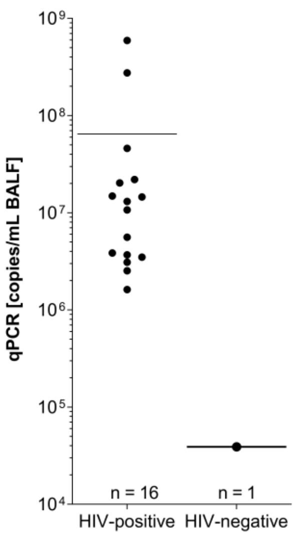


**Figure S4.** Our qPCR detects and quantifies the *mtLSU* rRNA gene specific for *P. jirovecii*. With standards developed and used in this qPCR, an absolute quantification of *P. jirovecii mtLSU* rRNA gene copies/mL BALF showed that all HIV-positive patients suffering from PCP had *P. jirovecii* copies above one million copies/mL BALF, while HIV-negative patients with PCP had a much lower copy number, as seen in the single patient's BALF with a copy number of 39,000 copies/mL BALF which was used for the *P. jirovecii* culture.

**S5.**

***HIV inactivation in BALF samples prior Pneumocystis cultures and control of HIV eradication in running Pneumocystis cultures was efficient.***

Regarding the guidelines for laboratory protection in Thuringia and prior authorisation of working with BALF from HIV-positive patients by the authority for genetic engineering, we had to verify that the HI viruses, which may present in the collected BALF samples [172] from the HIV-positive patients, were inactive or eradicated.

Chamoun-Emanuelli *et al.* published a new substance, PD404,182, which was able to inactivate HIV, Hepatitis C, and Herpes viruses in human samples [173,174]. Based on this substance, we developed a transport- and cell culture-inactivation protocol as follows: Prior shipment, the BALF aliquot for *P. jirovecii* culture was mixed with transport medium containing 30 µM PD404,182. The transport was done at room temperature and then the sample was frozen at -80°C until use for culture. After thawing, 1 mL of BALF was centrifuged at 4,800 g for 10 min at RT, the supernatant was discarded and the pellet was resuspended in 1 mL *Pneumocystis* culture medium (DMEM-C with DMEM low glucose (Sigma Aldrich, Germany), 10% fetal calve serum (FCS; Biochrom/Merck, Germany), 4 mM L-glutamine, 400 U/mL penicillin, 200 µg/mL streptomycin, 5 µg/mL amphotericin B) including 30 µM PD404,182. The BALF sample was then incubated at 37°C for 1 hour and, again, centrifuged at 4,800 g for 10 min at RT. After discarding the supernatant, the pellet was resuspended in 30 mL DMEM-C and washed another 2 times to remove all PD404,182 residues, and possibly the most HI-viruses. After the washing procedure and a final centrifugation, the BALF pellet containing the *P. jirovecii* organisms, was resuspended in the desired volume of *Pneumocystis* culture medium.

For verification of our HIV inactivation protocol, following samples from every patient were frozen at -80°C prior HIV testing: 1.1 mL of initial BALF without additives, 1.1 mL BALF with transport medium (1:2), and 1.1 mL medium derived from the *Pneumocystis* culture on day 0, 3, 7, and 14. The samples were frozen at -80°C until further analysis.

After carefully thawing the samples, RNA isolation and HIV-specific quantitative rtPCR was performed at the Laboratory for Medical Microbiology at the University Jena Hospital (accredited laboratory for HIV and other microbial tests, DAKKS number D-ML-13144-02-00).

As shown in several culture experiments, the inactivated HIV RNA was detectable for a maximum of three days in our *Pneumocystis* cultures, but most cultures were negative after the inactivation procedure. So, using this HIV inactivation protocol prior *Pneumocystis* culture

experiments for ensuring laboratory safety, we were able to use HIV-contaminated BALF samples.
When treated with 30 µM PD404,182 at 37°C for one hour, the HI viral load dropped below the detection limit of the HIV rt qPCR and remained negative. So, the treatment combined with the medium exchange led to a sample which can be used without the risk of a possible HIV infection when having a laboratory accident.
For laboratory safety, we used double inactivation of PD404,182 because during transport, the samples have room temperature. This could be too low for HIV inactivation – Chamouni *et al.* published 37°C at one hour as optimal temperature. So, we repeated the inactivation with modified procedure at 37°C prior *P. jirovecii* culture. But, when testing overnight inactivation at RT alone, it was also sufficient, and all cultures were HIV negative after a maximum of three days. This delay in HIV eradication was due to PD404,182 mainly inactivates the virus, but it is still present in the culture medium until the first complete medium exchange.

**S6.**

***The sensitivity and specificity of BALF staining methods for P. jirovecii was low and cannot detect small numbers of P. jirovecii, as present in HIV-negative BALF samples.***

The staining with Grocott methenamine silver stain (GMS) followed by microscopy was still the gold standard for *Pneumocystis* diagnostics. Nevertheless, all staining methods were often false-negative, especially in beginning PCP or PCP in HIV-negative patients [154, 175]. For comprehensiveness, we analysed 500 µL of our samples with GMS and DiffQuick staining and some samples with a specific DFT, which stains cysts and sporozoites in parallel, but had a lower specificity than GMS. In our samples, the sample volume for staining had to be less than the optimal 2 mL BALF to have enough sample volume for culture but was enough to have positive staining results.
HIV-negative immunosuppressed patients develop more severe symptoms at lower fungal loads than HIV-positive patients, as seen in our but also other studies. Geographic origin and mt85 genotype do also influence the organism burden in those patients [176-178]. Additionally, all PCP patients harbour several different *Pneumocystis* strains or haplotypes with varying replication rates [178, 179]. These factors may, beside sample transport condition effects, explain also the growth variances in our cultures.

**S7.**

***Estimation of growth by measuring either total ATP or lactic acid levels was not sufficient for P. jirovecii cultures with feeder cells, but also less sufficient for axenic cultures.***

Merali *et al.* described the first axenic culture system for rat-derived *P. carinii* and used ATP measurement for detecting *Pneumocystis* growth [30]. They found that the ATP content is greatly altered by the extraction process. But, if using their optimized technique, we only detected alterations of ATP, but no exploitable results. This may be due to the co-factors, like microbial contamination of the *P. jirovecii* culture by bacteria and possibly fungi derived from the original BALF, may alter the ATP values extremely particularly during the first days of *P. jirovecii* culture. After few days of culture, these contaminations will be eradicated by high-dose antibiotic and antimycotic additives in the *P. jirovecii* culture medium.

**S8.**

***Screening for possible bacterial and fungal contamination in Pneumocystis cultures did not detect contamination with other bacteria and fungi.***

For detection of possible bacterial and fungal contaminations present in the initial BALF or growing up during the *Pneumocystis* culture, 100 µL of culture supernatant with floating *P. jirovecii* (fPj) and 100 µL of scraped sessile *P. jirovecii* (sPj) were mixed, and 50 µL each were plated onto Columbia agar (sheep blood agar 5%) and Sabouraud agar plates at day 0, 6, and 14 of *P. jirovecii* culture. The agar plates were incubated at 37°C and 31°C for 14 days.

After 14 days of *P. jirovecii* culture medium incubation on Columbia and Sabouraud agar, no contamination with bacteria or fungi other than *Pneumocystis* was detected. Nevertheless, three of the long-time axenic flask cultures (culture V14, V14, and V18) showed yeast and bacteria contamination during the examination with DIC microscopy and were therefore discontinued.

**S9**

***P. jirovecii culture on A549 lung carcinoma cells was used for the first culture attempt in comparison to axenic P. jirovecii culture, but A549 cells hampered all further analyses.***

For culture on feeder cells, A549 cells (DSMZ No. ACC 107, $10^6$ cells/mL) were cultured in 24-well plates using DMEM-C until 70% confluence.

In general, all analysis methods, like staining, DIC and electron microscopy, qPCR quantification, and optimisation experiments with various growth conditions and medium supplements, were performed as used for the axenic cultures and were described in the main text.

**S9.1. Initial *P. jirovecii* culture of single patients with DMEM-C medium on A549 cells**

*P. jirovecii* cultures on A549 cells were the basis of our experiments, as this were most successful in animal pathogenic *Pneumocystis* (**Table S3**). The axenic culture performed in parallel proved to be more advantageous quite quickly. Co-cultures on feeder cells could also be useful for future studies of fungal-host interactions. As a result, we continued the majority of medium optimisation experiments with A459 co-cultures in parallel with axenic cultures and briefly present them here. DMEM-culture medium (DMEM-C, **main Text Table 2**) was the initial medium for both, axenic and A549 feeder cell *P. jirovecii* cultures. Methodology, sampling, and medium exchange was identical to the axenic experiments (see **main text 2.6.2**).

Prior to the culture attempt, the patient's BALF was analysed and *P. jirovecii* was quantified according to the axenic culture attempts (see main text). The concentration of the *P. jirovecii* culture inoculum was then adjusted to 1-2x$10^6$ *mtLSU rRNA* copies/mL DMEM-C medium.

In five initial cultures with BALF from single patients, *P. jirovecii* inoculum concentration was comparable in the culture with A549 feeder cells and the culture without feeder cells (1.79x$10^6$ ± 1.54x$10^6$ copies/mL and 1.85x$10^6$ ± 1.56x$10^6$ copies/mL).

In *P. jirovecii* cultures on A549 cells, an assessment of *P. jirovecii* clusters was not possible by DIC microscopy during the first week. The cells covered all potential *P. jirovecii* clusters (**main text Figure 3**). In cultures older than 14 days, larger clusters of trophic forms were present and were loosely attached to the A549 cells (**main text Figure 4**) and to each other, partially with membrane blurring between the spores (**main text Figure 4C-D**) as visible by electron microscopy in axenic cultures. This initial culture attempt showed a maximum 27.8-fold increase in *P. jirovecii* in cultures with A549 cells.

**Table S3. (Table 1 continued):** Significant animal-derived *Pneumocystis* spp. culture attempts.

| Year pub-lished | *Pneumocystis* strain and host species | Feeder cells | Medium | Supplements and concentration | Culture conditions | Duration | Start inoculum and *Pneumocystis* growth | Non-successful experiments / cell lines | References |
|---|---|---|---|---|---|---|---|---|---|
| | **Rat-derived *P. carinii*** | | | | | | | | |
| 1977 | *P. carinii* (rat) | Vero<br>Chang liver<br>MRC-5 | Eagle essential medium | 10% FCS<br>Penicillin<br>Streptomycin | 37°C | 4 months (with sub-cultures, up to three passages) | Inoculum: 1 mL of lung preparation (Pc concentration not mentioned)<br>Growth: no Pc concentration mentioned | LLC-MK-2<br>FL<br>McCoy | [63] |
| 1978 | *P. carinii* (rat) | VERO | MEM<br>Medium 199 | 2% FBS | 37°C | 7 days<br>Passage every 24 hrs | Inoculum: $1.3x10^5$ to $8.5x10^5$ cysts/culture of 1-2 mL<br><br>Growth: Max. 11-fold increase in cysts 72hrs post inoculation | Little growth (3-fold increase) in owl monkey kidney, baby hamster kidney, and AV-3 cell cultures, and no growth in WI-38 cells and secondary chicken fibroblast cultures<br>Maximum 3-4 passages, then decline | [54] |
| 1979 | *P. carinii* (rat) | WI-38<br>MRC-5 | Eagle medium | 2 – 10% FCS<br>50 µg/mL streptomycin<br>100 U/mL penicillin G potassium<br>10 U/mL nystatin | 35°C<br>No $CO_2$ | 10 days<br>Subcultures from 4-5 days old cultures | Inoculum: 1 mL supernatant derived from $1cm^3$ ground rat lung; number of Pneumocystis in inoculum not measured<br>Growth: $117.9x10^6$ | High variances between growth of rat isolates – even if the rat lung used for culture had high organism loads | [55] |
| 1985 | *P. carinii* (rat) | A549<br>WI-38 | A549: DMEM<br>WI-38: HMEM | 25 mM HEPES<br>20% HyClone FBS / serum (rat, chicken, swine, human, horse) from several companies<br>1000 U penicillin<br>1000 µg streptomycin<br>0.5 µg amphotericin B<br>NCTC vitamin mixture 107 formula no. 78-0776 or no vitamins | Room temp – 30 – 35 – 37 – 41°C (optimal: 30-37°C for Pc on A549 cells);<br>Stationary – rocking 6 rpm;<br>With/without 5%$CO_2$ | 20 days | Inoculum:<br>Optimal: $1x10^6$ organisms/mL<br>Maximal: $1x10^8$ organisms/mL<br>Minimum: $1x10^5$ organisms/mL<br><br>Growth: in both cell lines<br><br>Subculture/passaging: 3 successful passages 7 days after start with 12-fold organism increase, or 14 days after start resulting in a steady-state or 21 days after start with 4-fold organism increase | Inhibitory effect (in both cell lines):<br>- temperature 41°C<br>- 0.05% saponin<br>- rocking / moving the cultures<br><br>Source of serum (rat, swine, human) or temperature led to a decrease in growth in VA13 cells | [29] |

| Year pub-lished | *Pneumocystis* strain and host species | Feeder cells | Medium | Supplements and concentration | Culture conditions | Duration | Start inoculum and *Pneumocystis* growth | Non-successful experiments / cell lines | References |
|---|---|---|---|---|---|---|---|---|---|
| 1985 | *P. carinii* (rat) | WI-38<br>MRC-5 | Eagle medium | 2 – 10% FCS<br>50 µg/mL streptomycin<br>100 U/mL penicillin G potassium<br>10 U/mL nystatin | 5% $O_2$<br>5-10% $CO_2$<br>35°C | 10 days | Inoculum:<br>1 mL supernatant derived from 1 $cm^3$ ground rat lung; number of Pneumocystis inoculum not measured<br><br>Growth:<br>peak at day 6<br>max organism number (peak day) 1.8 to $17.5x10^6$ in Pc on WI-38 cells and 0.7 to $16.1x10^6$ in Pc on MRC-5 cells<br>more constant growth on WI-38 cells | 2 to 3 passages / harvests possible<br>Growth decreased in subcultures | [56] |
| 1993 | *P. carinii* (rat) | HEL (A549 and L-132 gave no good results) | Eagle MEM (DMEM, RPMI-1640, medium 199 gave no good results) | 10% FCS<br>Antibiotics (penicillin, streptomycin, amphotericin B, no concentration mentioned)<br>Medium exchange every 3rd day<br>Passaging every 3-5 days | 5% $CO_2$<br>37°C | 42 days | Inoculum: 1 to $3x10^7$ *P. carinii*/mL in 24-well tissue culture plates<br><br>Growth: peak at day 6 to 9 or 10, but after that decline | Cell-bound and supernatant Pc were counted separately (30% were adherent to cells)<br>Infection of rats after 41 days of in vitro culture was successful | [39] |
| 2009<br>2011 | *P. carinii* (rat) | axenic | RPMI-1640 | 20% calf serum<br>penicillin (200 U/ml)<br>streptomycin (200 µg/ml)<br>amphotericin B (0.5 µg/ml) vancomycin (5 µg/ml)<br>S-adenosyl-L-methionine<br>farnesol<br>vitamins and amino acids as described before<br><br>PET track-etched membrane cell culture inserts<br>Transwell inserts<br>Millicell-CM hydrophilic PTFE membranes<br>Millicell-HA insert | 36°C<br>5% $CO_2$ | 7 – 14 (21) days | Inoculum: $1x10^6$ to $1x10^8$<br><br>Growth:<br>Less extensive biofilm formation than *P. murina* from mice (thickness of <15 µm) | S-adenosyl-L-methionine led to a dramatic decrease of viability within 24 hrs in *P. carinii* and *P. murina*<br><br>Farnesol led to decreased viability in *P. carinii* | [31, 43] |
| 2006 | *P. carinii* (rat) | axenic | RPMI-1640 | 20% calf serum<br>1x MEM vitamins<br>non-essential amino acids (conc. not mentioned), L-glutamine (conc. not mentioned), 100 IU penicillin, and 100 mg/mL streptomycin<br>Start inoculum: $5x10^7$ organisms/mL | Standard: 21% $O_2$ and 5% $CO_2$<br>microaerophilic:10-15% $O_2$, 7-15% $CO_2$<br>anaerobic: <1% $O_2$, 10% $CO_2$ | 7 days | Inoculum: $5x10^7$ *P. carinii*/mL<br><br>Growth: ATP measurement only: 15,000 RLU under microaerophilic conditions vs 3,000 RLU under standard conditions after 7 days | Anaerobic conditions: decline after the first day | [57] |
| 2013 | *P. carinii* (rat) | axenic | DMEM | 10% FBS<br>100 U/mL penicillin<br>100 µg/mL streptomycin | 37°C<br>5% $CO_2$ | 2-4 days | Inoculum: $0.75x10^4$ *P. carinii*/mL or $2.5x10^5$ *P. carinii*/mL<br><br>Growth: from 6x105 to 10x105 organisms within 40 hours, then decline | Decline of *Pneumocystis* life stages after 2 days in all cultures | [42] |

| Year published | *Pneumocystis* strain and host species | Feeder cells | Medium | Supplements and concentration | Culture conditions | Duration | Start inoculum and *Pneumocystis* growth | Non-successful experiments / cell lines | References |
|---|---|---|---|---|---|---|---|---|---|
| | **Mouse-derived *P. murina*** | | | | | | | | |
| 1989 | *P. carinii* from nude mouse (= *P. murina*?) | Axenic | DMEM<br>L-15 | 10% FBS<br>2-mercaptoethanol 100 µM<br>bathocuproine sulphonate (BCS) 50 µM<br>penicillin 1000 U/mL<br>streptomycin 1000 µg/mL<br>amphotericin B 0.5 µg/mL<br>cysteine (or cystine?) | 37°C<br>5% $CO_2$ | 7 days<br>Sub-cultures until day 15 | Inoculum: 1 mL pellet from minced and cell-lysed mouse lung isolate per 24 well plate, ca. $2.8x10^6$ (DMEM) to $5.5x10^6$ (DMEM) sporozoites<br><br>Growth: L-15 slightly better than DMEM,<br>3-fold increase after day 2, then stagnation (at ca. $1x10^7$ sporozoites in both cultures) until day 7 | Gradually decreasing Pc when cultured with serially passages | [41] |
| 1989 | *P. carinii* from nude mouse (= *P. murina*?) | Axenic | DMEM<br>L-15 | 10% FBS<br>2-mercaptoethanol 100 µM<br>bathocuproine sulphonate (BCS) 50 µM<br>penicillin 1000 U/mL<br>streptomycin 1000 µg/mL<br>amphotericin B 0.5 µg/mL<br>cysteine (or cystine?) | 37°C<br>5% $CO_2$ | 7 days<br>Sub-cultures until day 15 | Inoculum: 1 mL pellet from minced and cell-lysed mouse lung isolate per 24-well plate, ca. $2.8x10^6$ (DMEM) to $5.5x10^6$ (DMEM) sporozoites<br><br>Growth: L-15 is slightly better than DMEM,<br>3-fold increase after day 2, then stagnation (at ca. $1x10^7$ sporozoites in both cultures) until day 7 | Gradually decreasing Pc when cultured with serial passages | [41] |
| 2009<br>2011 | *P. murina* | Axenic | RPMI-1640 | 1-20% calf serum<br>penicillin (200 U/ml)<br>streptomycin (200 µg/ml)<br>amphotericin B (0.5 µg/ml) vancomycin (5 µg/ml)<br>S-adenosyl-L-methionine<br>vitamins and amino acids as described before<br><br>PET track-etched membrane cell culture inserts<br>Transwell inserts<br>Millicell-CM hydrophilic PTFE membranes<br>Millicell-HA insert | 36°C and 37°C<br>5% $CO_2$ | 7 – 14 (21) days | Inoculum: $1x10^6$ to $1x10^8$<br><br>Growth:<br>More extensive biofilm formation than P. carinii from rats (thickness of 15 µm) | S-adenosyl-L-methionine led to a dramatic decrease of viability within 24 hrs in P. carinii and P. murina | [31, 43] |
| | **Rabbit-derived *P. oryctolagi*** | | | | | | | | |
| 1989 | *P. oryctolagi* (rabbit) | Vero cell line<br>Axenic | CMRL-1066<br>RPMI-1640 | 10% FBS<br>2-mercaptoethanol (dosage?)<br>Bathrocuprine sulphonate (dosage?)<br>Antibiotics (dosage?)<br>Cysteine (dosage?) | 37°C<br>50% $O_2$, 5% $CO_2$, 45% $N_2$ | 41 days<br>Medium exchange every 2nd day | Inoculum: not mentioned<br><br>Growth: 4-fold increase after 41 days in axenic culture | No growth on Vero cells | [26] |

*P. jirovecii* clusters increased in cultures with and without feeder cells, as seen by *P. jirovecii* specific antibody staining and microscopy, as well as qPCR **(Figure S2)**. Culture on feeder cells revealed faster and more stable growth than without feeder cells, however, the A549 feeder cells hampered all further analyses of the organism, as next generation sequencing. Nevertheless, qPCR copy numbers did not increase consistently due to clustering of the *P. jirovecii*.

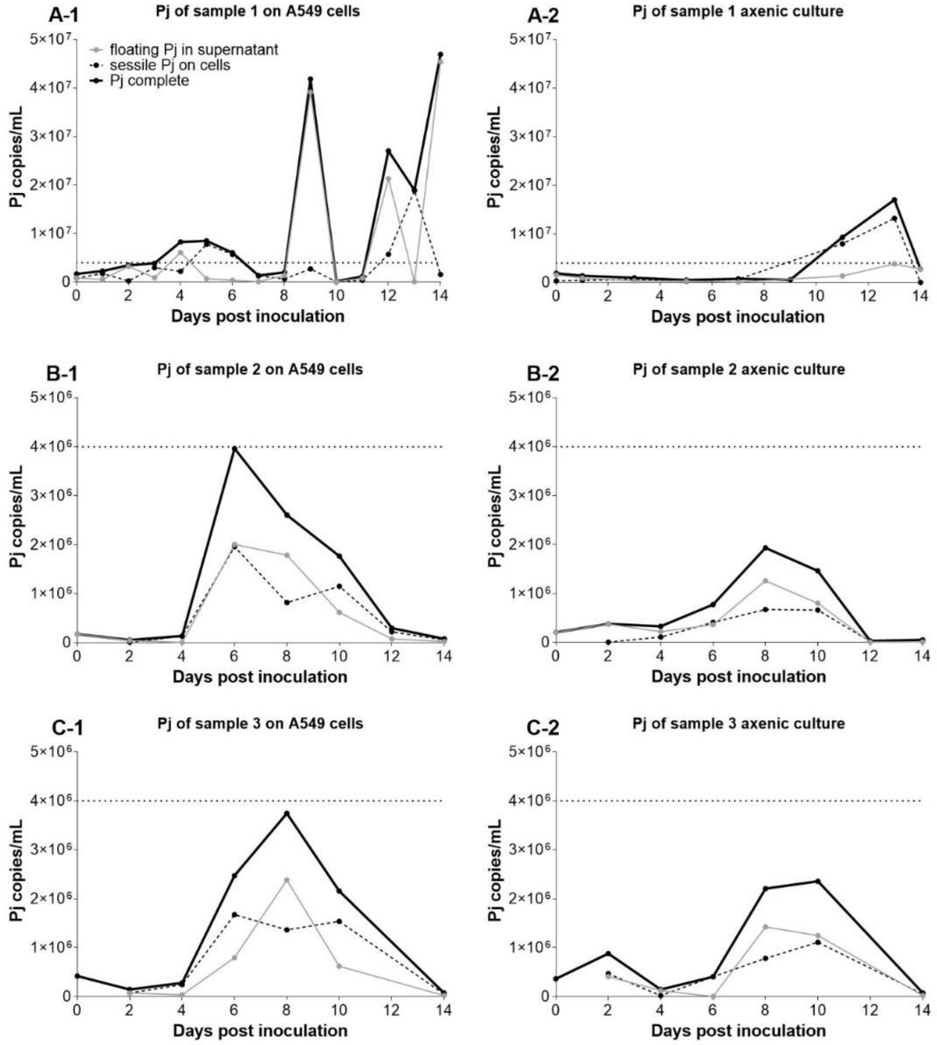


**Figure S5.** *P. jirovecii* growth in cultures from BALF of three patients in DMEM-C medium with A549 cells (left side) and axenic culture (right side). Samples were analysed separately by qPCR for floating *P. jirovecii* (fPj) and sessile *P. jirovecii* (sPj) and the results used to calculate the total number of *P. jirovecii*. After day six of culture, *P. jirovecii* clusters were visible in all wells. The dotted lines show the maximum growth achievable with this medium in all culture attempts, which was only surpassed by *P. jirovecii* growth in the cultures from sample 1 (A-1 and A-2). In these cultures, a growth peak can be seen after 6-10 days. Two further cultures showed a decline or steady-state in both with and without A549 cells, possibly due to a low *P. jirovecii* inoculum.

Three of five cultures with A549 showed an increase in *P. jirovecii* clusters, but growth was not constant, but with peaks. This was also seen in *P. carinii* studies before, where the organisms were counted microscopically. The maximum copy number counted was $5x10^7$ copies/mL in the culture from BALF sample of patient 1 on A549 cells (**Figure S5**).

A differentiation of floating and sessile *P. jirovecii* was not beneficial for all further analyses, therefore we did not examine them separately in all further experiments. As seen by two of the five cultures, not in all *P. jirovecii* strains an increase of clusters and qPCR copies/mL culture medium was visible (**Figure S5** and main text **Figure 5**). This might be due to the transport of these two strains, or culture conditions. Therefore, we added *P. jirovecii* from two to four patients to the culture medium for all future experiments to ensure viable strains in the culture. As every patient harbours more than one *P. jirovecii* strain at the same time, a culture of one single strain might be impossible either.

### S9.2. *S*ub-cultures were successful in *P. jirovecii* A549 cultures with isolates from single PCP patients.

To verify the viability of the *P. jirovecii* during culture, subcultures were produced as follows: When harvesting *P. jirovecii* by scraping of the appropriate well at day 10, 200 µL *P. jirovecii* culture supernatant were resuspended through a 21G needle and mixed with 5 mL of new DMEM-C. Five wells of a 24-well plate were seeded with 1 mL of this *P. jirovecii* mix per well and cultured until day 41. In case of the co-culture with A549 cells, the 24-well plate was incubated with new A549 cells as described before prior *P. jirovecii* sub-culture.

Our data showed, that *P. jirovecii* can also be successfully sub-cultured, as seen in the experiment with sample 1 on A549 cells (**Figure S6**), where constant growth could be observed for the complete nine day sub-culture resulting in $1.96x10^7$ *P. jirovecii* copies/mL, which corresponds to a 48.8-fold increase.

For our long-term axenic flask cultures with fed-batch medium addition, a sub-culture was not necessary. Due to optimized growth conditions, the *P. jirovecii* could be cultured continuously.

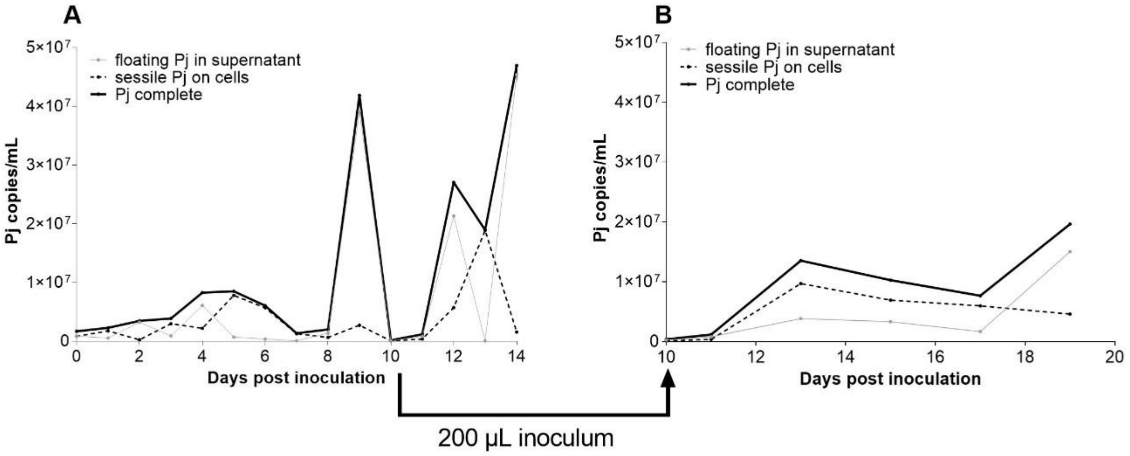


**Figure S6**. *P. jirovecii* sub-culture of an isolate from sample 1 on A549 cells. Initially, the *P. jirovecii* isolate was cultured on A549 cells for 14 days with medium exchange and separate sampling of floating and sessile *P. jirovecii* organisms every 2nd day (A). On day ten, 200 µL culture supernatant containing the scraped A549 cells and the *P. jirovecii* organisms was mixed with 6 mL fresh DMEM-C culture medium. One mL of this *P. jirovecii*-A549 mix was added to each well of a 24-well-plate and then incubated at 37°C and 5% CO2 (B). Sampling and medium exchange of this sub-culture was performed on day 10 (= day 0 of sub-culture) and then every 2nd day as in the initial culture. In axenic cultures, a sub-culture was performed by splitting the culture volume.

**S9.3. DMEM-C Medium optimisation of A549 cultures with mixed *P. jirovecii* strains.**
As in the axenic culture experiments performed in parallel on 6-well plates with DMEM-C medium (see **main text 2.6.2., 3.3.**), a combination of *P. jirovecii* strains from up to four different BALF samples led to more stable growth than using BALF of a single patient. A pH of 8.0 was optimal and no pH alteration was seen during the cultures. In the literature, temperatures between 31 and 40°C were used for animal *Pneumocystis* cultures [29, 56]. In our experiments, optimal *P. jirovecii* growth was observed at 37°C (**Table S4**).

***Table S4.*** *Optimisation* of basic *P. jirovecii* culture conditions in 6-well culture with A549 cells and axenic culture without feeder cells in DMEM-C medium. - decline/starvation of *P. jirovecii*, (+) steady state (*P. jirovecii* count stable over the whole culture duration), + slow growth, +++ fast growth with large *P. jirovecii* clusters.

| Conditions and additives | Conditions tested | *P. jirovecii* growth on A-459 cells |
|---|---|---|
| | 7.0 | - |
| | 7.5 | + |
| **pH** | 8.0 | +++ |
| | 8.5 | + |
| | 9.0 | - |
| **Temperature** | 31°C | - |
| | 35°C | (+) |
| | 37°C | +++ |
| **Coated plates** | Gelatine | - |
| | Poly-L-lysin | - |
| **Transwell plates** | | - |
| **Medium exchange** | every 2 days | +++ |
| | every 6 days | + |
| | None | - |

The addition of mono- and disaccharides to the DMEM-C medium increased the growth of *P. jirovecii* under cell-free conditions (see **main text Figure 6**) but only few when co-cultured with A549 cells (**Figure S7**).
Increasing the FCS concentration from 10% to 20 and 30% revealed no positive effect for *P. jirovecii* cultures on A549 cells. Culture at pH 9, or 31°C temperature, brought about a strong decline in *P. jirovecii* organisms. Ferric pyrophosphate (80 µg/mL) had no effect on *P. jirovecii* cultures on A549 cells.

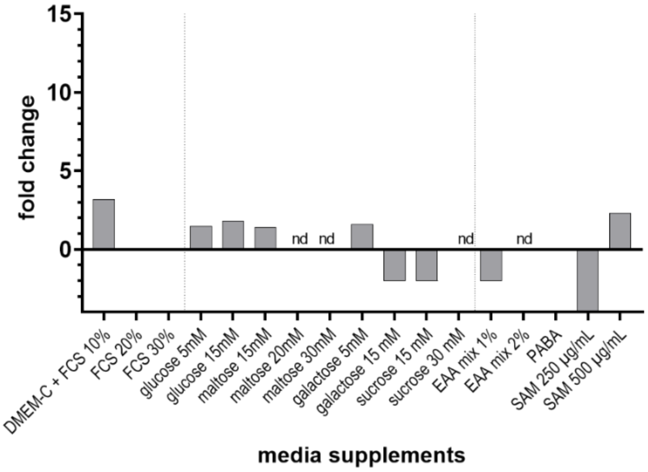


**Figure S7.** Culture supplements and concentrations tested for improving the growth of *P. jirovecii* with DMEM-C medium on A549 cells. *P. jirovecii* were grown in 24-well plates for

10-14 days and quantified by qPCR. Growth was examined microscopically every 2nd day. EAA – essential amino acid mix 50x, NEAA - non-essential amino acid mix, SAM – S-adenosyl-L-methionine, PABA – para aminobenzoic acid.

***S10.***

***P. jirovecii electron TEM and REM microscopy protocol for analysis of BALF and culture samples.***

Pellets made from BALF samples taken from PCP patients, and for comparison, *P. jirovecii* cultures that had been grown on A549 cells were fixed at least overnight with a solution of 1% paraformaldehyde and 2% glutaraldehyde in 0.1M phosphate buffer at pH 7.3. After washing in 0.1M phosphate buffer and centrifugation (5000 g / 10 min), the pellets were resuspended in 100 µL of the same buffer.

For scanning EM, the resuspended sample was adhered for one hour to a glass coverslip pre-coated overnight with an 0.01% poly-L-lysine solution (Sigma-Aldrich, Germany). The coverslip was dehydrated with an ascending acetone series (70%, 90% and twice pure acetone) for 10 min each prior to critical point drying using $CO_2$ as an intermedium with three washes (Emitech K850 critical point dryer, UK). Specimens were mounted on aluminium stubs using adhesive carbon tape, sputter-coated with a layer of gold (approximately 10-15 nm) using a Bal-Tec SCD004 sputter coater (Balzers, Liechtenstein) and viewed with a field-emission scanning EM operated at 5 kV (Zeiss Merlin VP compact, Zeiss, Germany).

For transmission EM, the resuspended samples were mixed with 0.3% low melting agarose (Fluka, Germany) and, after centrifugation and hardening of the agarose, processed en bloc for embedding starting with a post-fixation step in 1% osmium tetroxide (Roth, Germany) followed by washing in water and subsequent dehydration in a graded acetone series. Epon resin (Serva, Germany) was infiltrated starting with a 1:1 acetone to resin mixture overnight, followed by pure resin for 4 hours. The samples were then transferred to rubber moulds and cured at 60°C for 2 days. After trimming, semi thin sections (0.5 µm) and thin sections (50-70 nm) were cut with a diamond knife (Diatome, Switzerland). Thin sections were collected on copper mesh grids and were stained with uranyl acetate and lead citrate for ultrastructural inspection with a Zeiss EM 902 or a Zeiss Libra120 transmission EM (Zeiss, Germany) operated at 80kV or 120kV. Digital images were acquired with CCD cameras (Proscan / TRS, Germany) using iTEM camera control and imaging software (Olympus, Germany).

***S11.***

***P. jirovecii axenic flask cultures in DMEM-O2 medium with mixed P. jirovecii strains showed increased growth measured by qPCR.***

Using DMEM-O2 medium, we analysed the optimal *P. jirovecii* organism inoculum, culture length, and medium supplementation time points in 22 axenic flask cultures to produce larger numbers of *P. jirovecii* (most important cultures: see main text, **Figure 9**, summary of all 22 cultures: **Figure S8**).

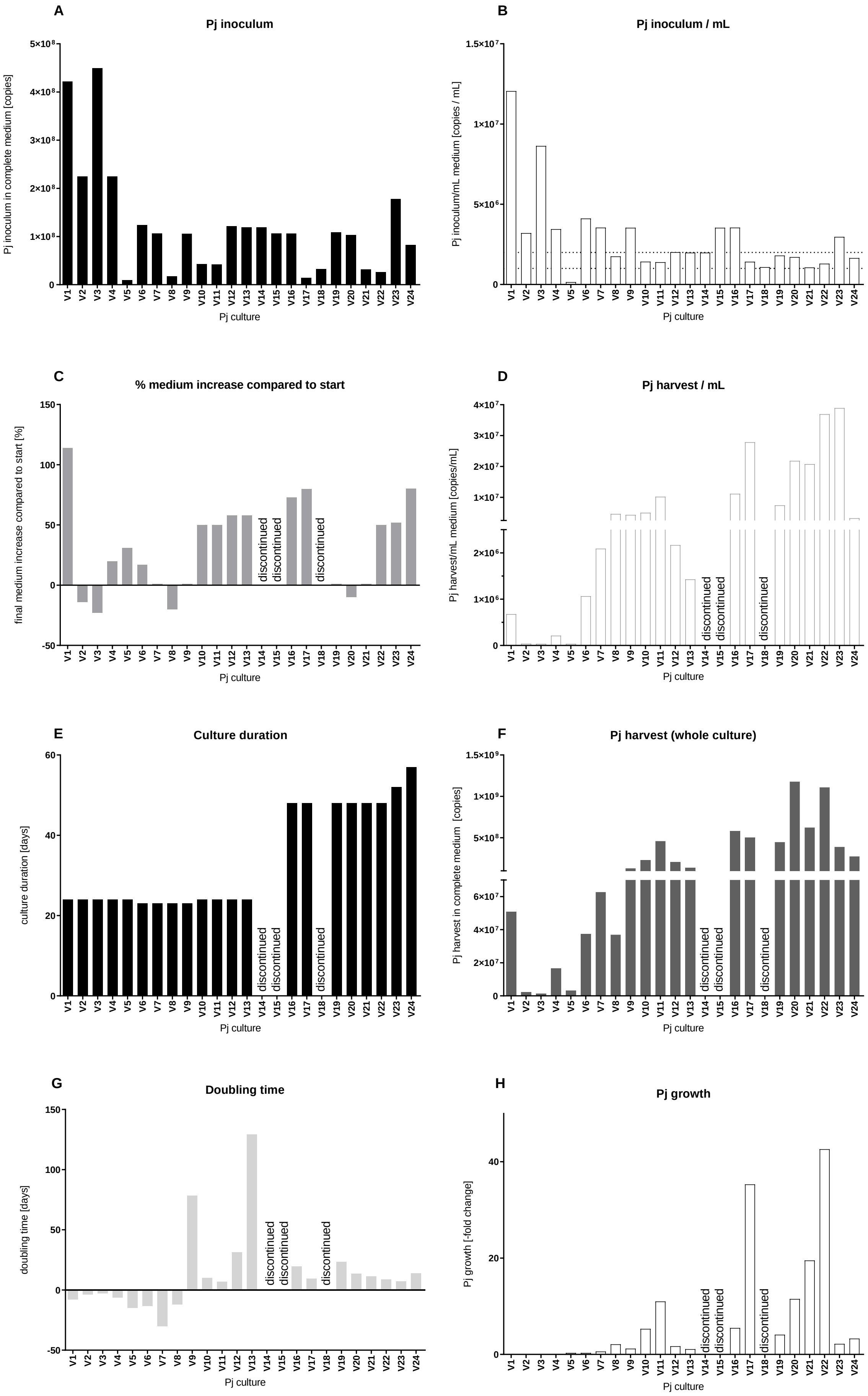
A
Pj inoculum
Pj inoculum in complete medium [copies]
Pj culture
B
Pj inoculum / mL
Pj inoculum/mL medium [copies / mL]
Pj culture
C
% medium increase compared to start
final medium increase compared to start [%]
discontinued
discontinued
discontinued
Pj culture
D
Pj harvest / mL
Pj harvest/mL medium [copies/mL]
discontinued
discontinued
discontinued
Pj culture
E
Culture duration
culture duration [days]
discontinued
discontinued
discontinued
Pj culture
F
Pj harvest (whole culture)
Pj harvest in complete medium [copies]
discontinued
discontinued
discontinued
Pj culture
G
Doubling time
doubling time [days]
discontinued
discontinued
discontinued
Pj culture
H
Pj growth
Pj growth [-fold change]
discontinued
discontinued
discontinued
Pj culture

**Figure S8.** Growth of *P. jirovecii* in 24 axenic long-term cultures with a minimum of 22 and a maximum of 57 days of fed-batch flask culture. Culture conditions except patient samples for inoculum, duration of culture, and final culture volume were not altered. All cultures were grown in DMEM-O2 medium (starting volume 10 mL/flask) and were cultured at 37°C and 5% $CO_2$. The medium was not exchanged completely, but samples for qPCR analysis were taken and increasing volumes of new medium were added on top of the old medium to dilute the growing *P. jirovecii* to appropriate densities in the flask and add fresh supplements (fed-batch culture). As seen after the first five cultures, a start inoculum of 1 to 2 million copies/mL culture medium volume was desirable, as both well plate cultures and flask cultures with higher or lower inoculum showed decreasing *P. jirovecii* copy numbers/mL medium. Nevertheless, due to *P. jirovecii* clumping, some cultures had higher concentrations of *P. jirovecii* inoculum. Medium increase of up to 60% during culture and a duration of 48 days led to the largest increase in *P. jirovecii* with an optimum doubling time of 6.9 to 8.8 days and a max. 42.6-fold increase.

**S12.**

***P. jirovecii growth was enhanced in axenic flask cultures with DMEM-O3 medium. Cluster number and cluster surface were increasing during a 15-day culture starting on day 36 of a running culture.***

For measuring *P. jirovecii* cluster growth, 7.5 mL of the V40 flask culture were centrifuged, dissolved in 4.5 mL DMEM-O3 medium and 0.5 mL was pipetted into four wells of an 8well LabTek chamber slide (Nunc), while the leftover was quantified by *mtLSU* qPCR. On day 0, 5, 10, and 15 of this experiment, well 4 was examined completely by meandering through the well with the CellObserver.Z1 microscope (Zeiss) with 100 to 400fold magnification, photography and counting of all *P. jirovecii* clusters, and measuring the visible surface of each cluster in $mm^2$. For each microscopic examination, well 4 was used, so that recognition of specific clusters was potentially possible.

Growth analysis at day 36 to 41 of culture V40 in DMEM-O3 medium showed constantly increasing *mtLSU* qPCR copy numbers, and both, microscopically increasing numbers and surfaces of *P. jirovecii* clusters during the 15 day-experiment (**Figure 11, Supplements Figure S9**).

Counting all organisms, even in a small culture sample with a few clusters, is impossible even in extremely small media volumes; moreover, due to the 3D shape of the clusters, measurement in Z-stacks would be necessary. Again, this would lead to a high error rate, as many trophic forms would be counted twice.

In order to have an impression of the sizes of the individual organisms in a cluster, the diameter and area of all clearly recognizable structures on the surface of a single cluster were determined using the measuring function of the Zeiss ZEN blue software V3.2 (**Figure 10**) and compared with the current sizes described in the literature for *P. jirovecii* as follows: Thin-walled haploid trophic forms, previously known as trophozoites, are 2 - 4 μm, diploid trophic forms are 2 - 10 μm and will develop to an ascus (previously: cyst) with 5 – 8 μm size [3, 6]. Trophic forms are the predominant form in the lungs [6], but also in our *P. jirovecii* cultures.

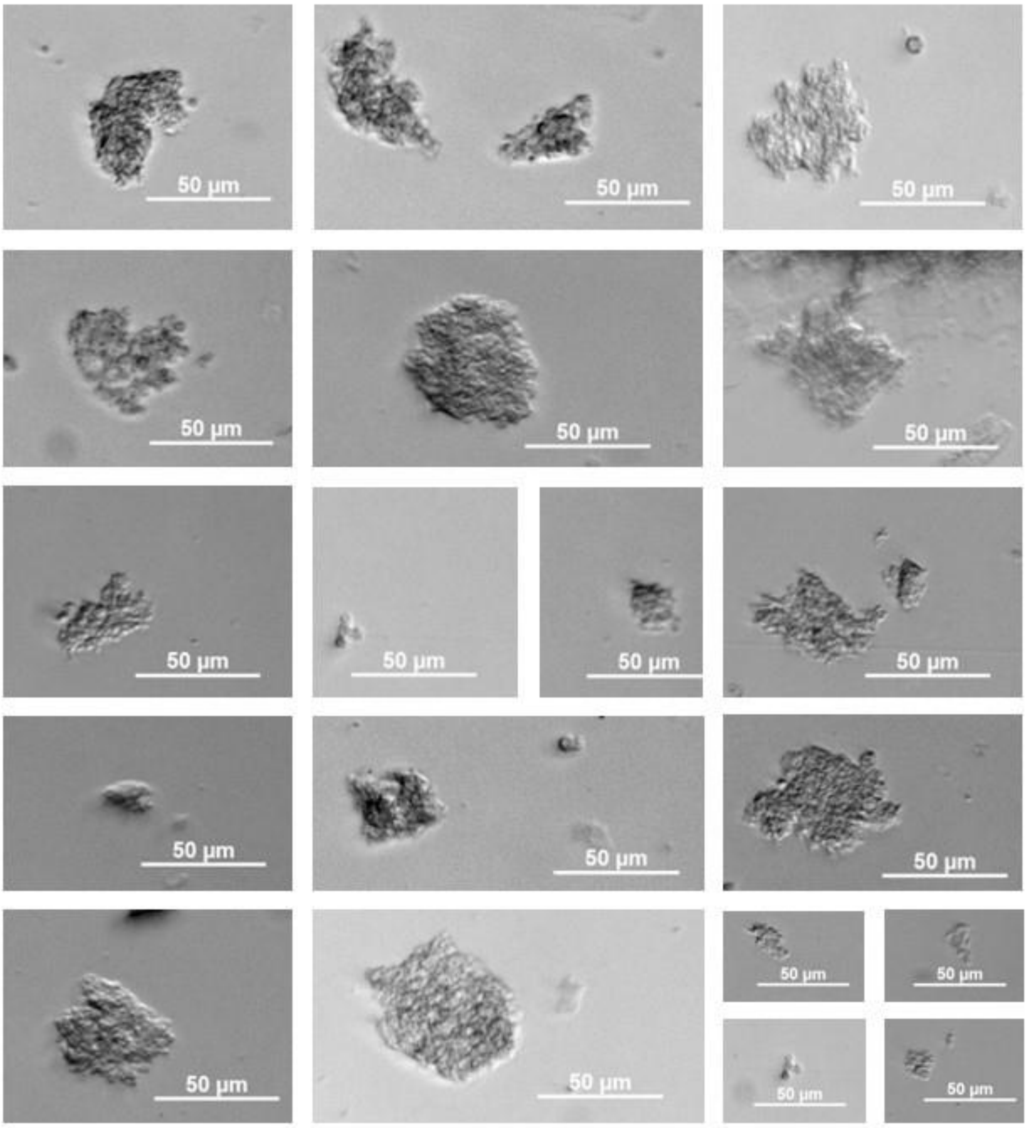


**Figure S9a-e.** Growth of *P. jirovecii* in DMEM-O3 medium, day 36 after *P. jirovecii* culture start, day 0 of the growth experiment. The chamber slide was incubated at 37°C and 5% $CO_2$ as were all other *P. jirovecii* cultures. Medium fed-batch was not performed in this experiment to avoid moving the *P. jirovecii* clusters. Each of the four upper wells of a chamber slide was filled with 0.5 mL DMEM-O3 containing *P. jirovecii* organisms harvested from the actual flask culture V40. Well 4 was examined thoroughly with DIC 400x magnification (Cellobserver microscope, Zeiss) on days 0, 5, 10, and 15. Clusters were photographed, counted and the surface was measured. **Figure S9a (actual picture)** shows the total number of *P. jirovecii* clusters on day 0 of the experiment (n=23), **Figure S9b** shows the *P. jirovecii* clusters on day 5 (n=36), **Figures S9c-d** show the *P. jirovecii* clusters on day 10 (n=134), **Figures S9e-h** show the *P. jirovecii* clusters on day 15 (n=291).

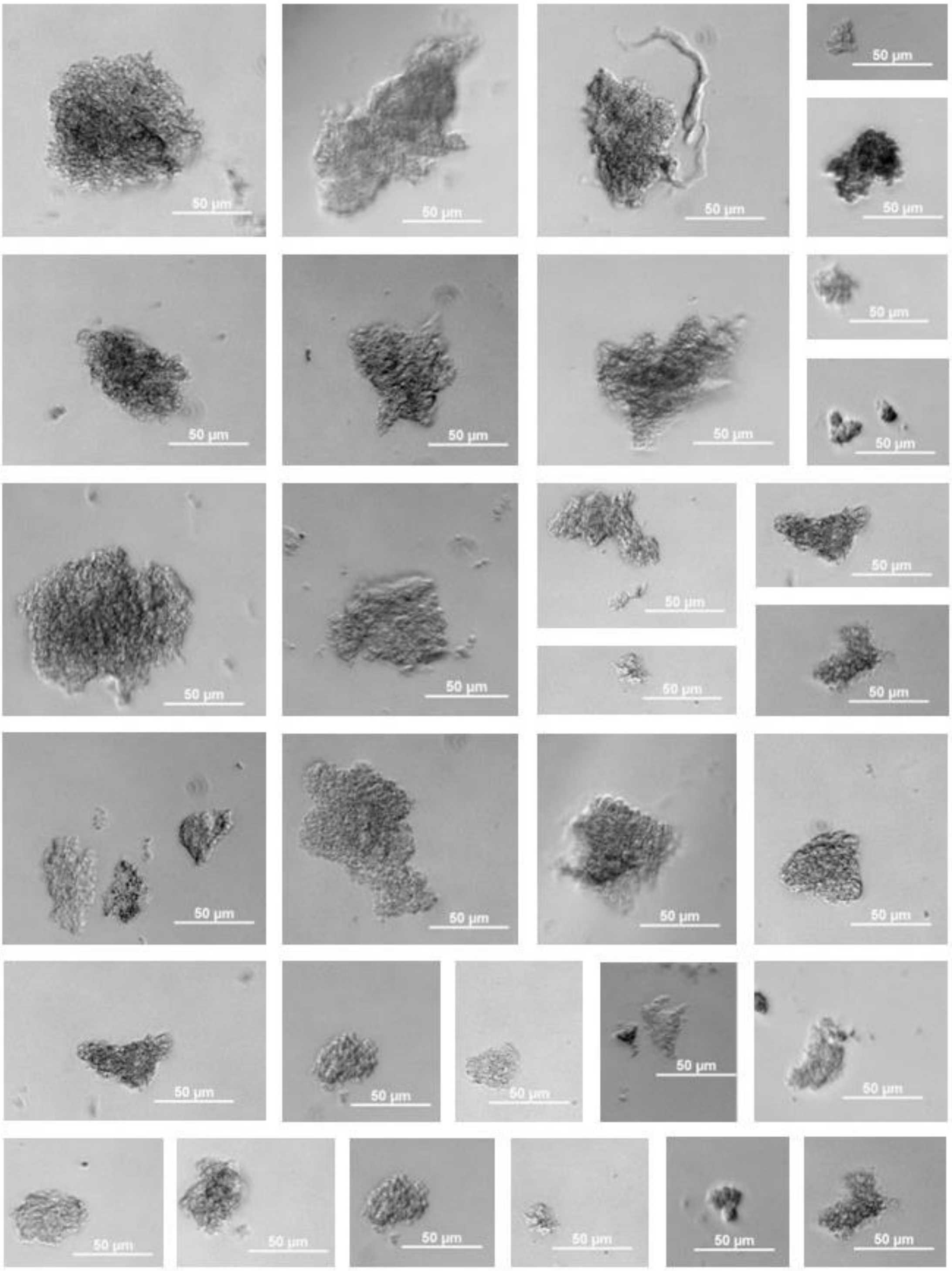


**Figure S9b.** *P. jirovecii* clusters at day 5 (n=36).

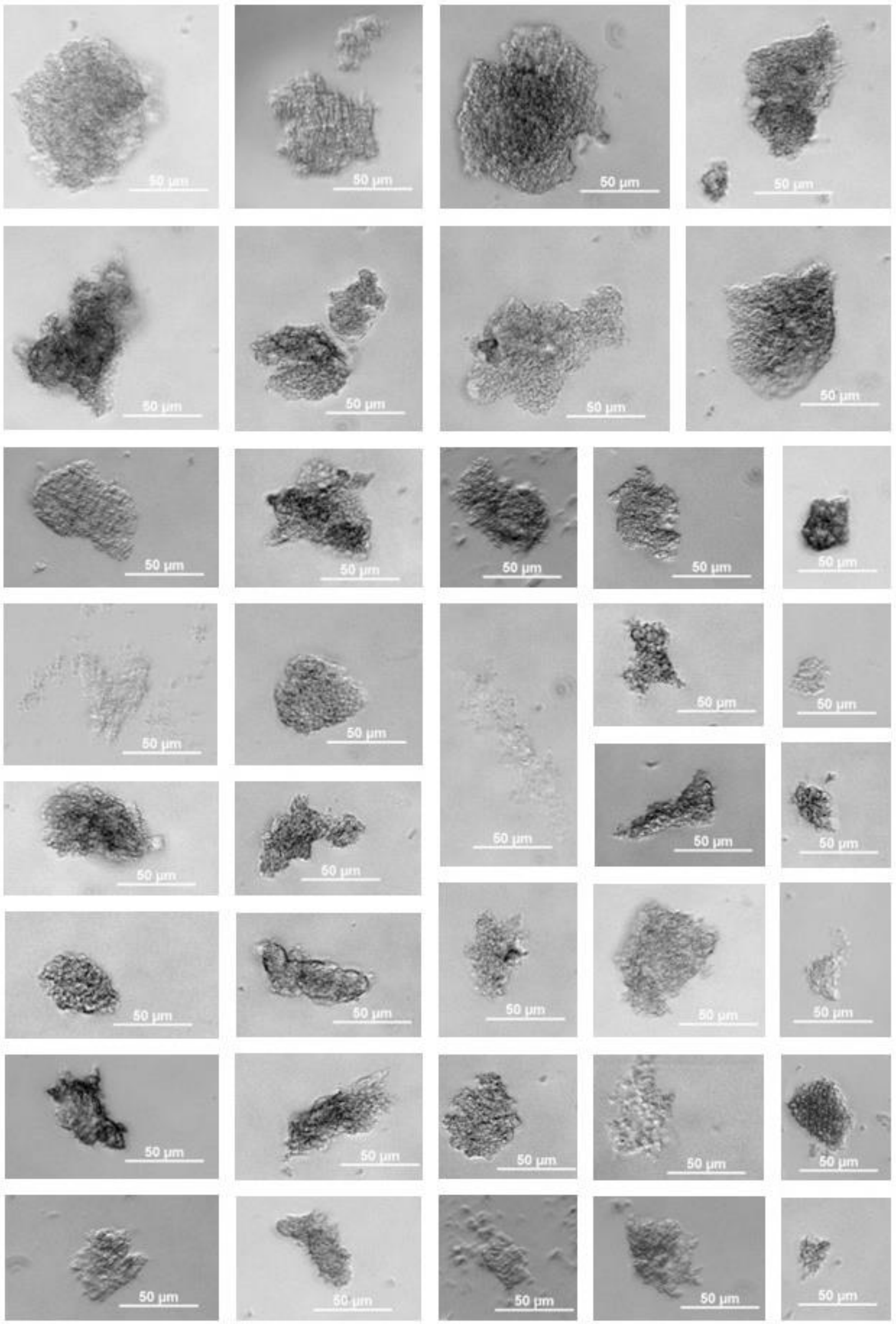


**Figure S9c.** *P. jirovecii* clusters at day 10 (n=134, continued at **Figure S9d**).

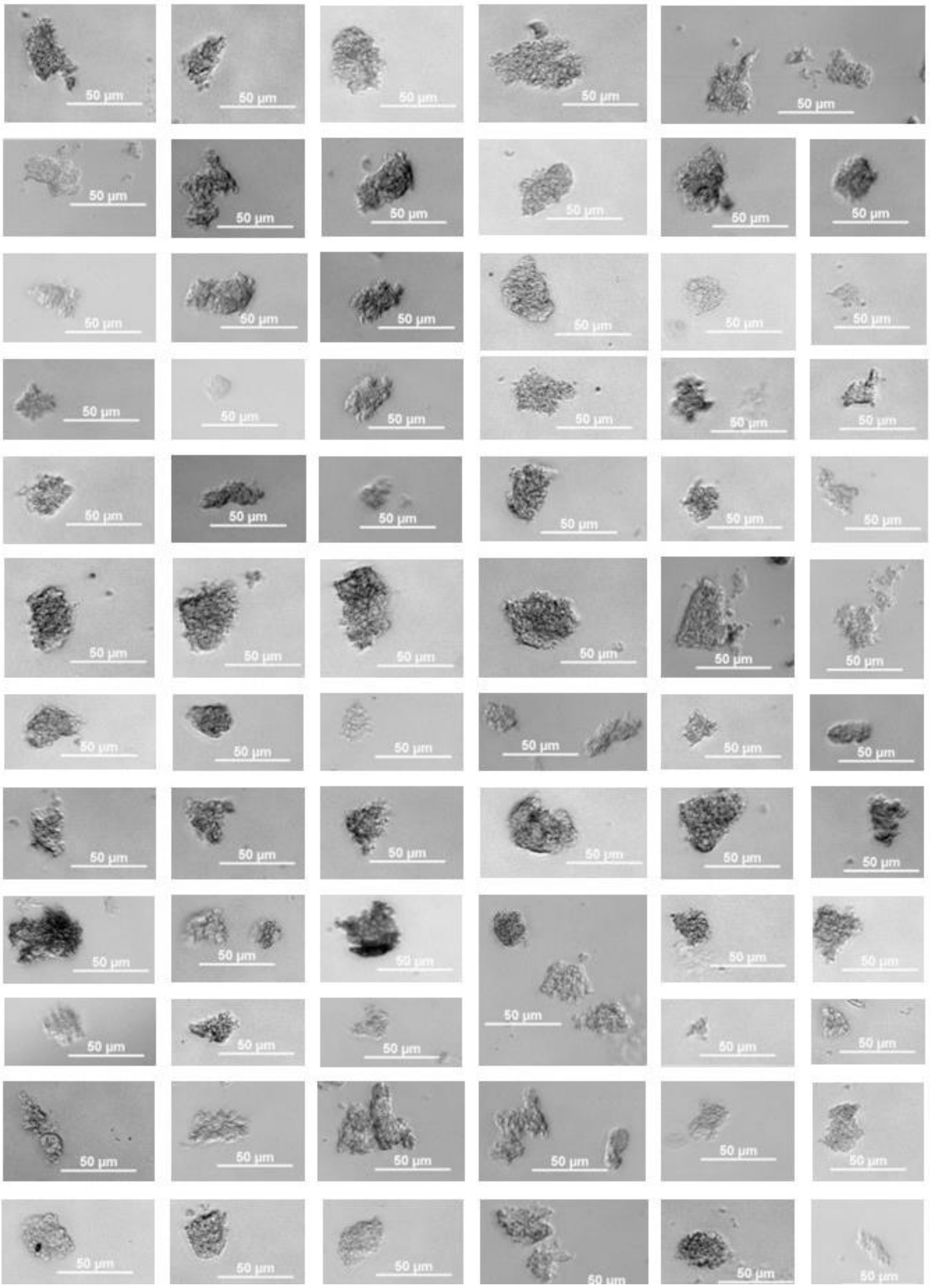

**Figure S9d.** *P. jirovecii* clusters at day 10 (n=134).

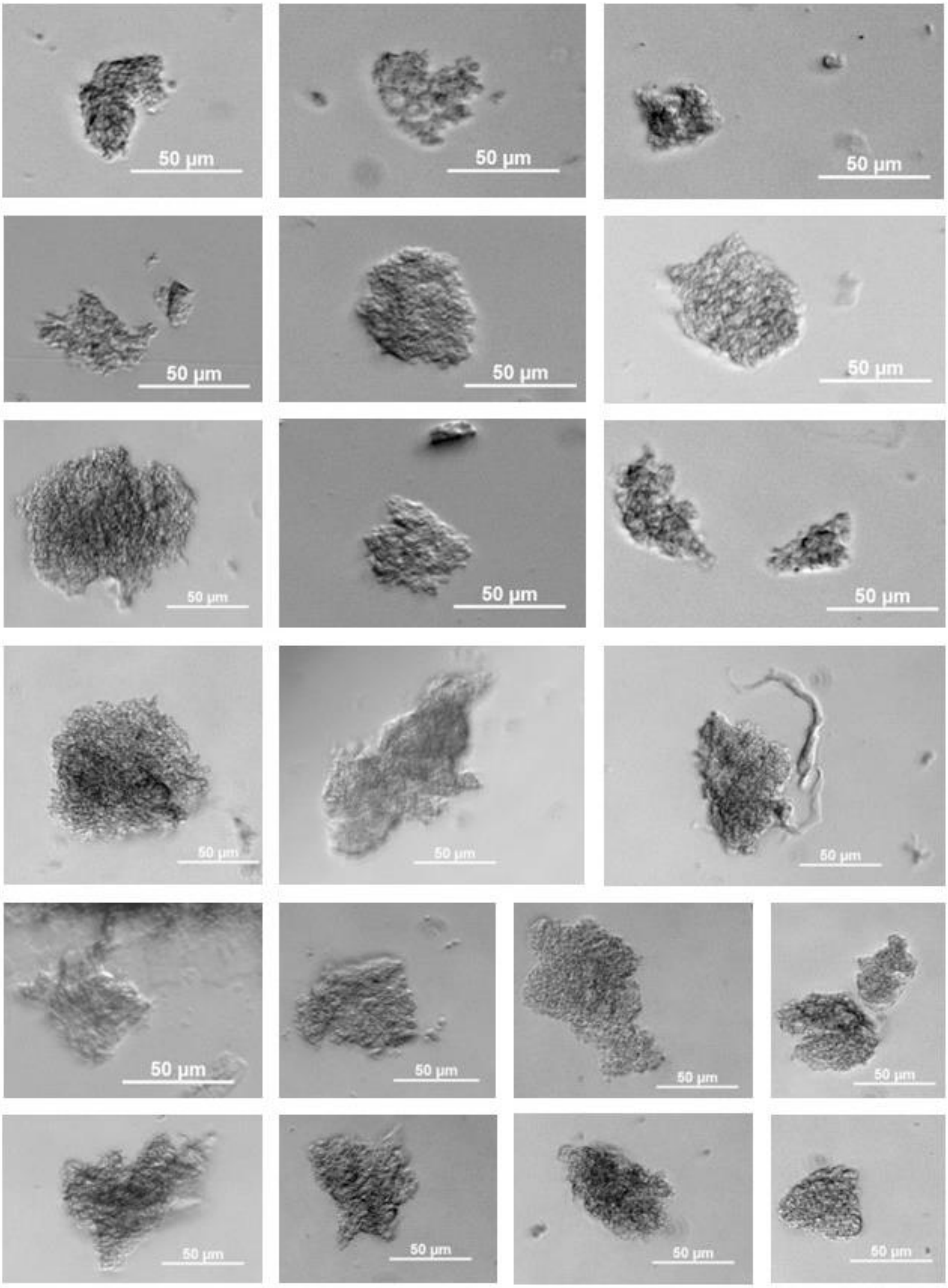


**Figure S9e.** *P. jirovecii* clusters at day 15 (n=291, continued at **Figure S9f-h**).

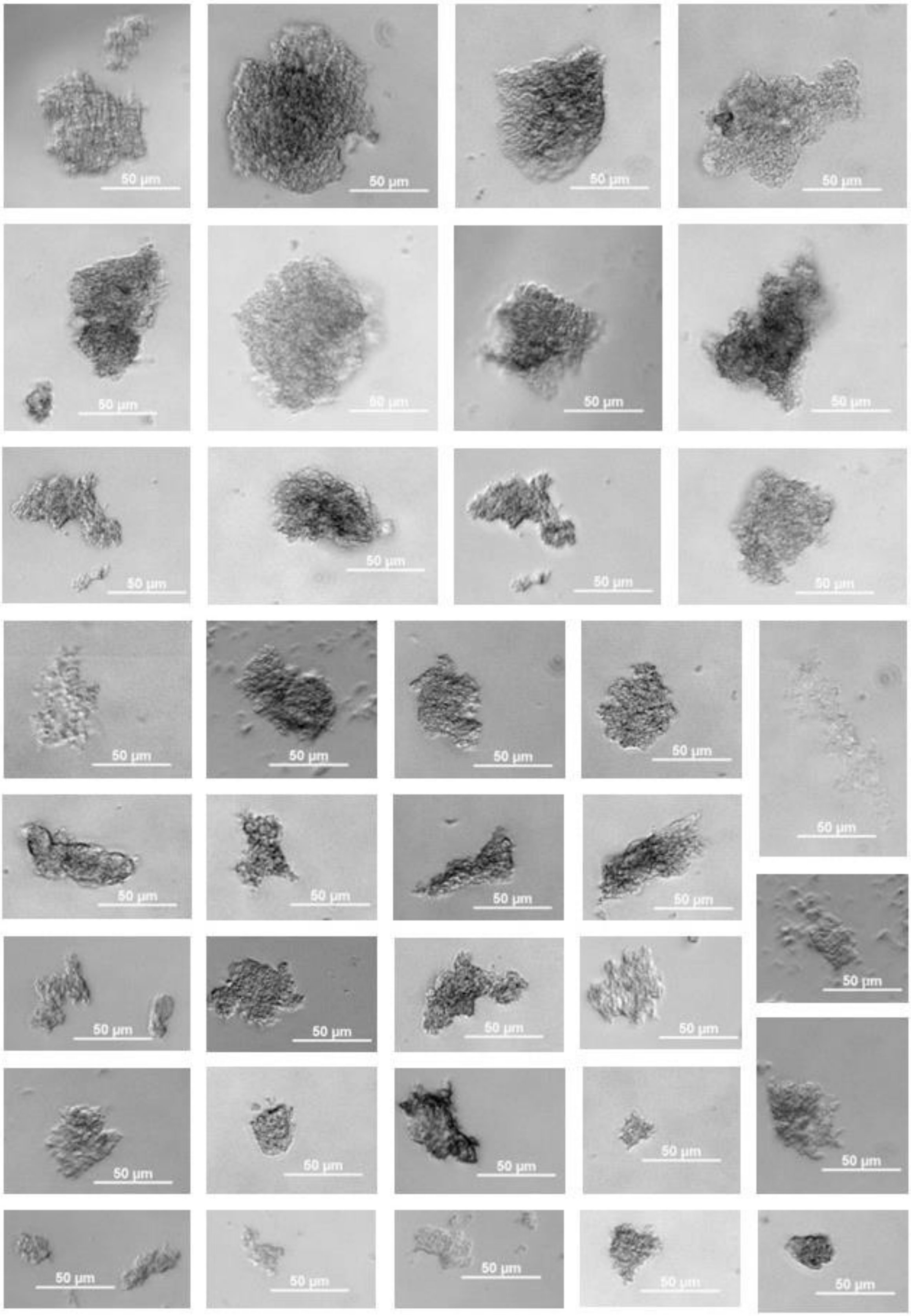


**Figure S9f.** *P. jirovecii* clusters at day 15 (n=291, continued at **Figure S9g-h**).

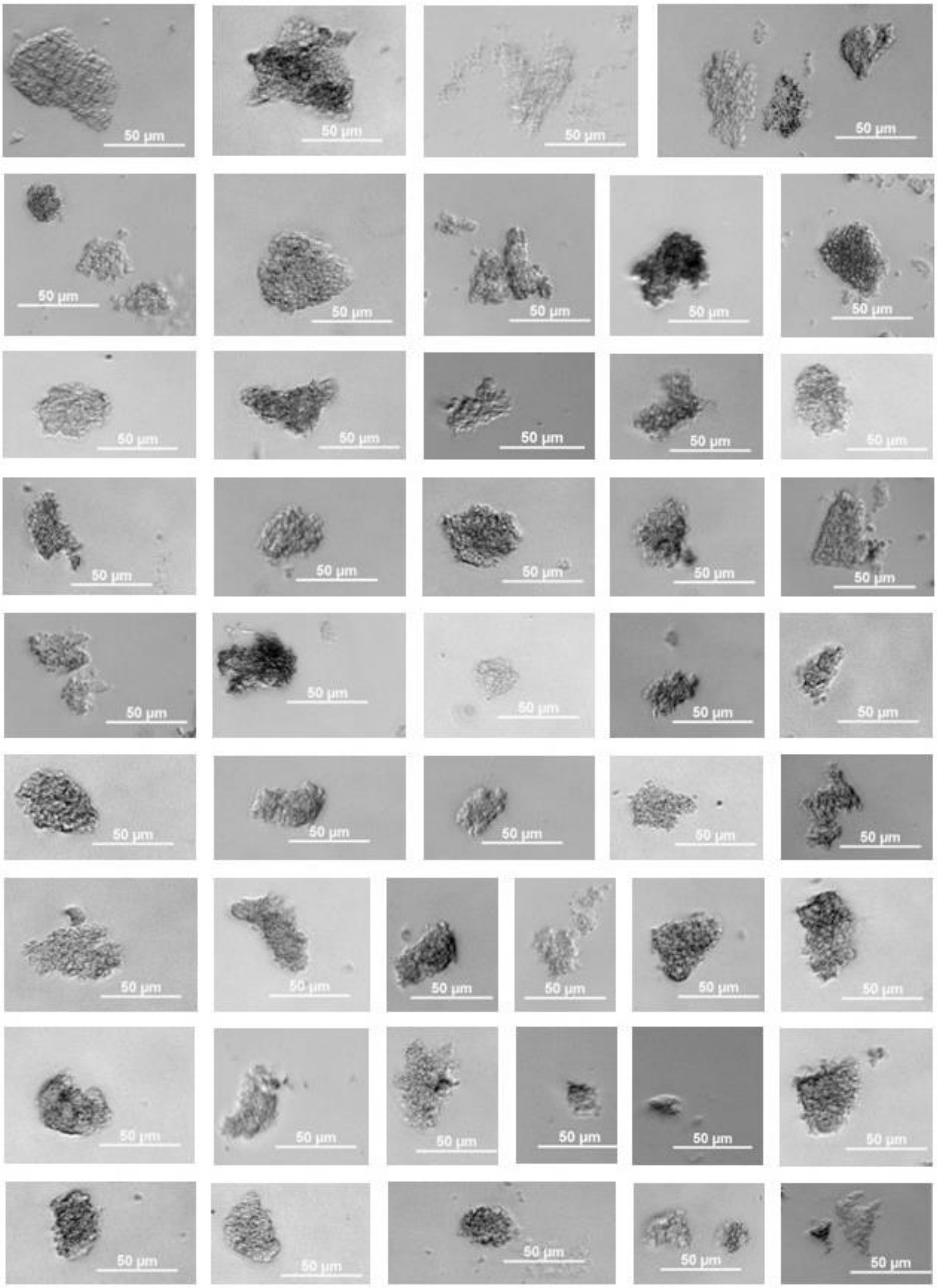


**Figure S9g.** *P. jirovecii* clusters at day 15 (n=291, continued at **Figure S9h**).

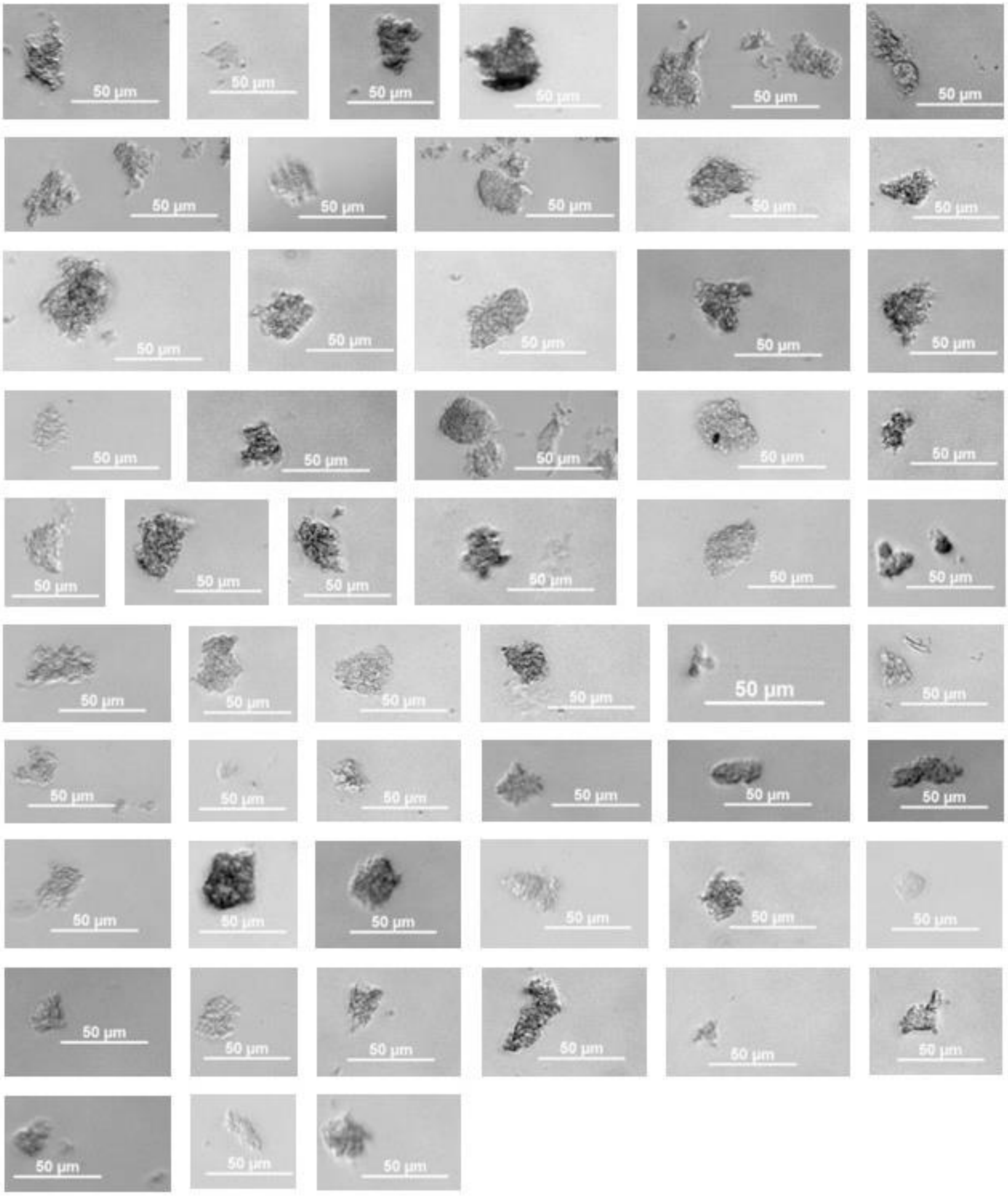

**Figure S9h.** *P. jirovecii* clusters at day 15 (n=291).

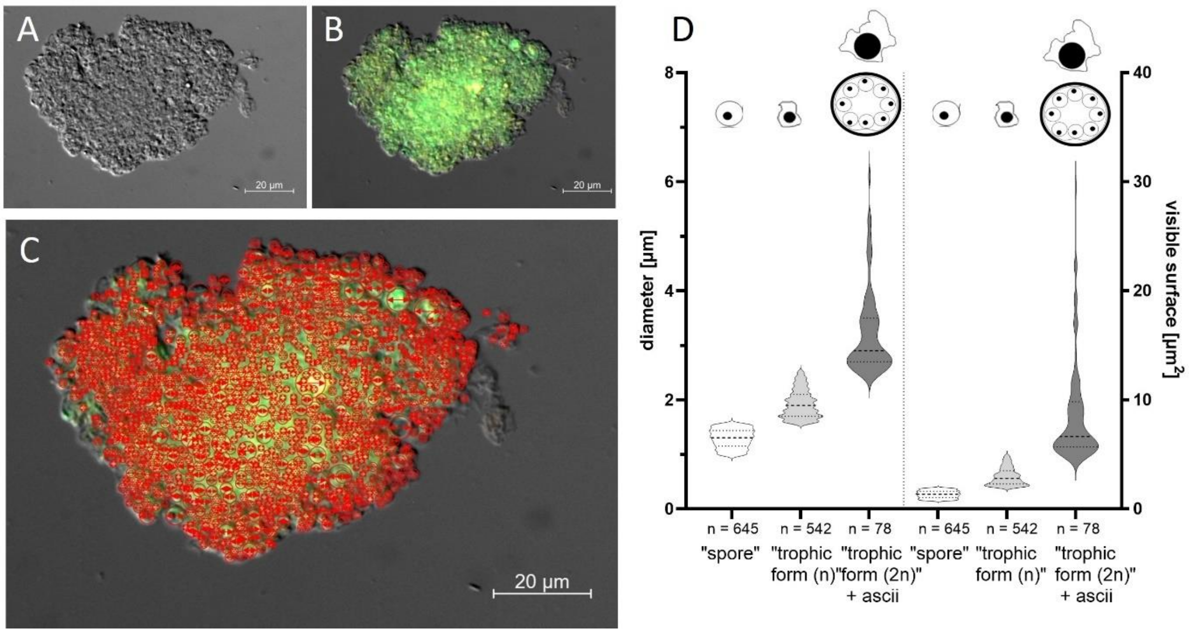


**Figure S10.** A single *P. jirovecii* cluster extracted from a culture in DMEM-O2 medium on day 14 contains thousands of individual organisms. To distinguish between different life stages (spore, haploid trophic form, diploid trophic form and ascus), the organism diameters on the surface of one single cluster were measured and differentiated by size into spore, haploid trophic form (n), and diploid trophic form (2n). In this cluster, spores and haploid trophic forms represented the majority of the organisms, diploid trophic forms that developed into ascii were rare.
